# Supplementary material for: In Vivo Hepatoprotective and Nephroprotective Activity of Acylated Iridoid Glycosides from Scrophularia hepericifolia
Source: Biology (Basel). 2021 Feb 12;10(2):145. doi: 10.3390/biology10020145 (PMC7918105; doi:10.3390/biology10020145)
Supplement: Supplementary file 1 [file biology-10-00145-s001.pdf]

*Supplementary Materials*

# In Vivo Hepatoprotective and Nephroprotective Activity of Acylated Iridoid Glycosides from *Scrophularia Hepericifolia*

**Maged S. Abdel-Kader and Saleh I. Alqasoumi**

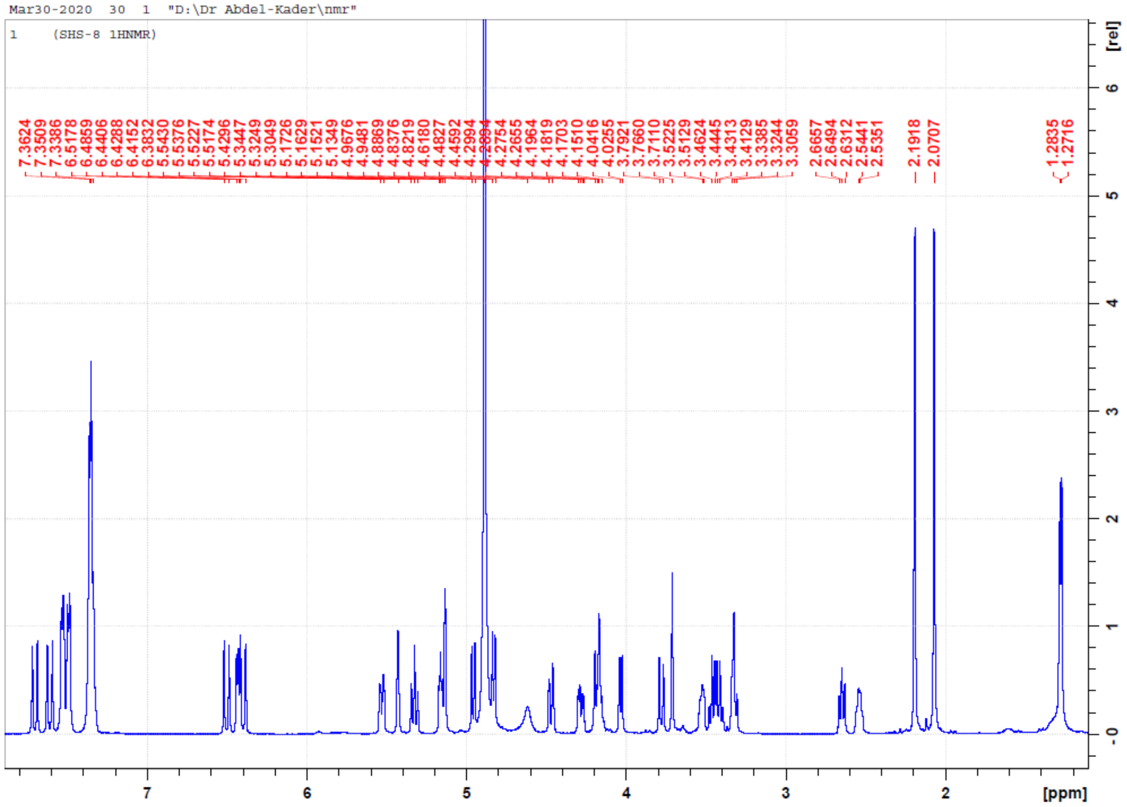

**Figure S1.** $^{1}$HNMR spectrum of compound 1.

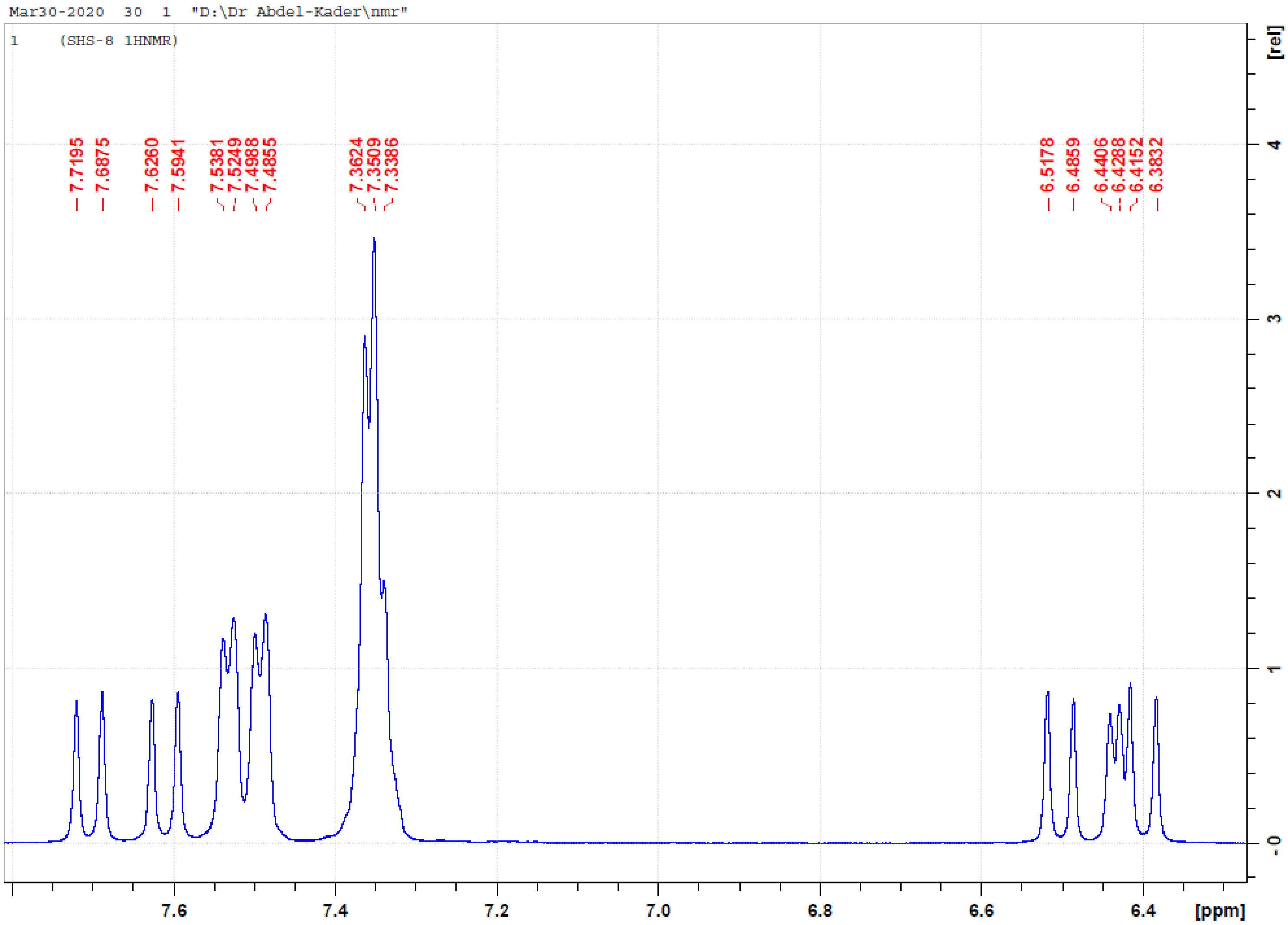

**Figure S2.** $^{1}$HNMR spectrum of compound 1 (Expansion).

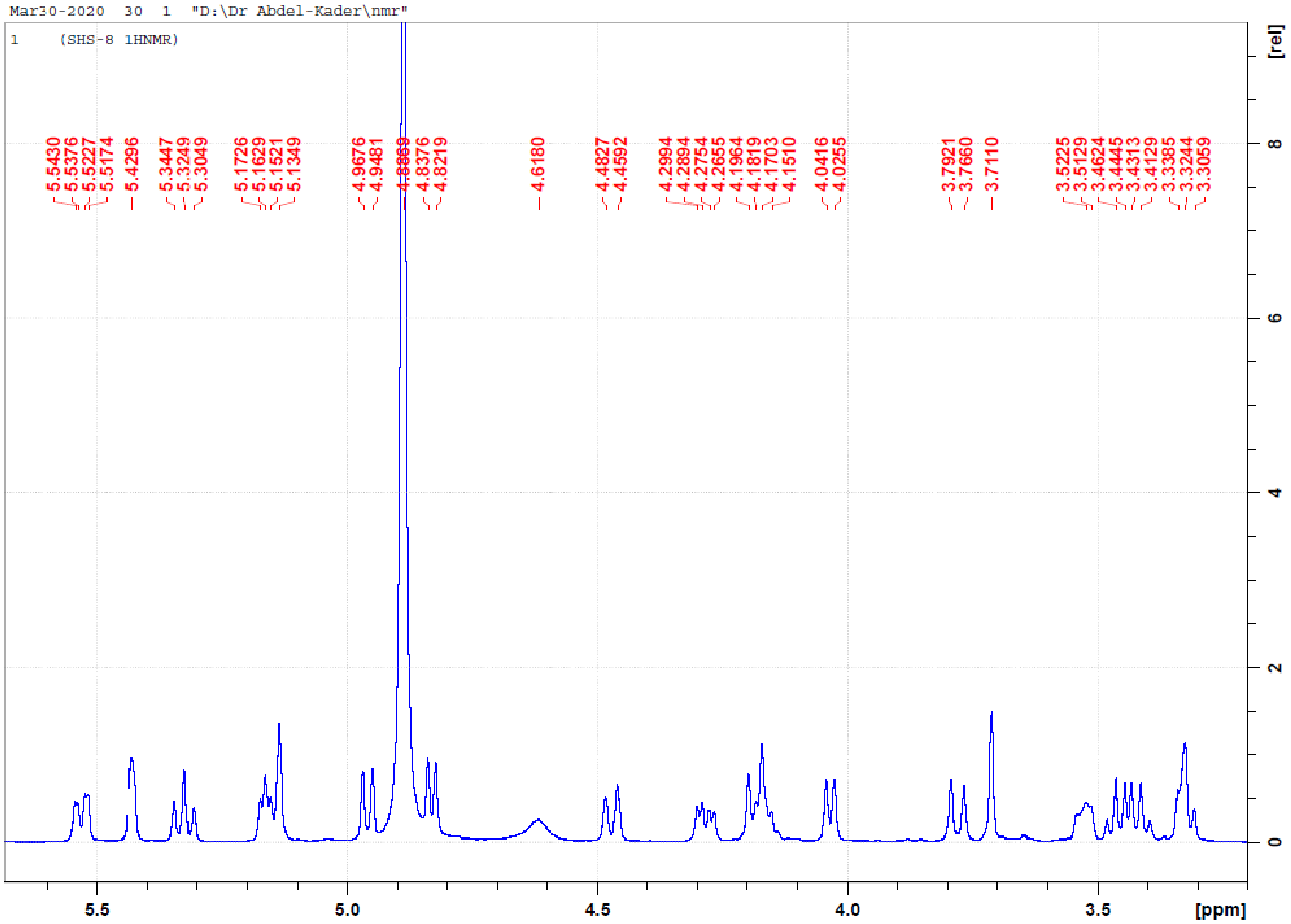


**Figure S3.** $^{1}$HNMR spectru of compound 1 (Expansion).

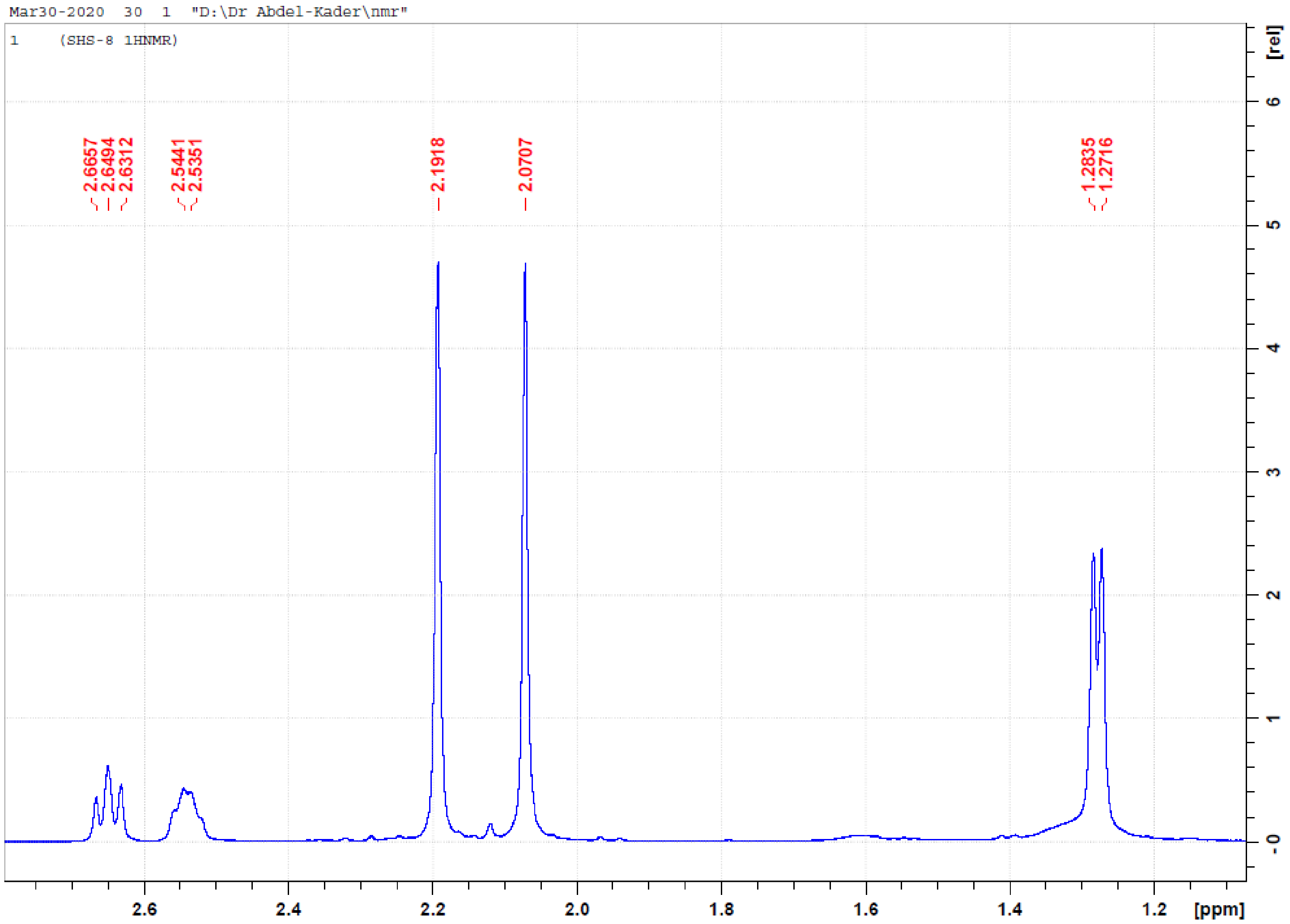


**Figure S4.** $^{1}$HNMR spectrum of compound 1 (Expansion).

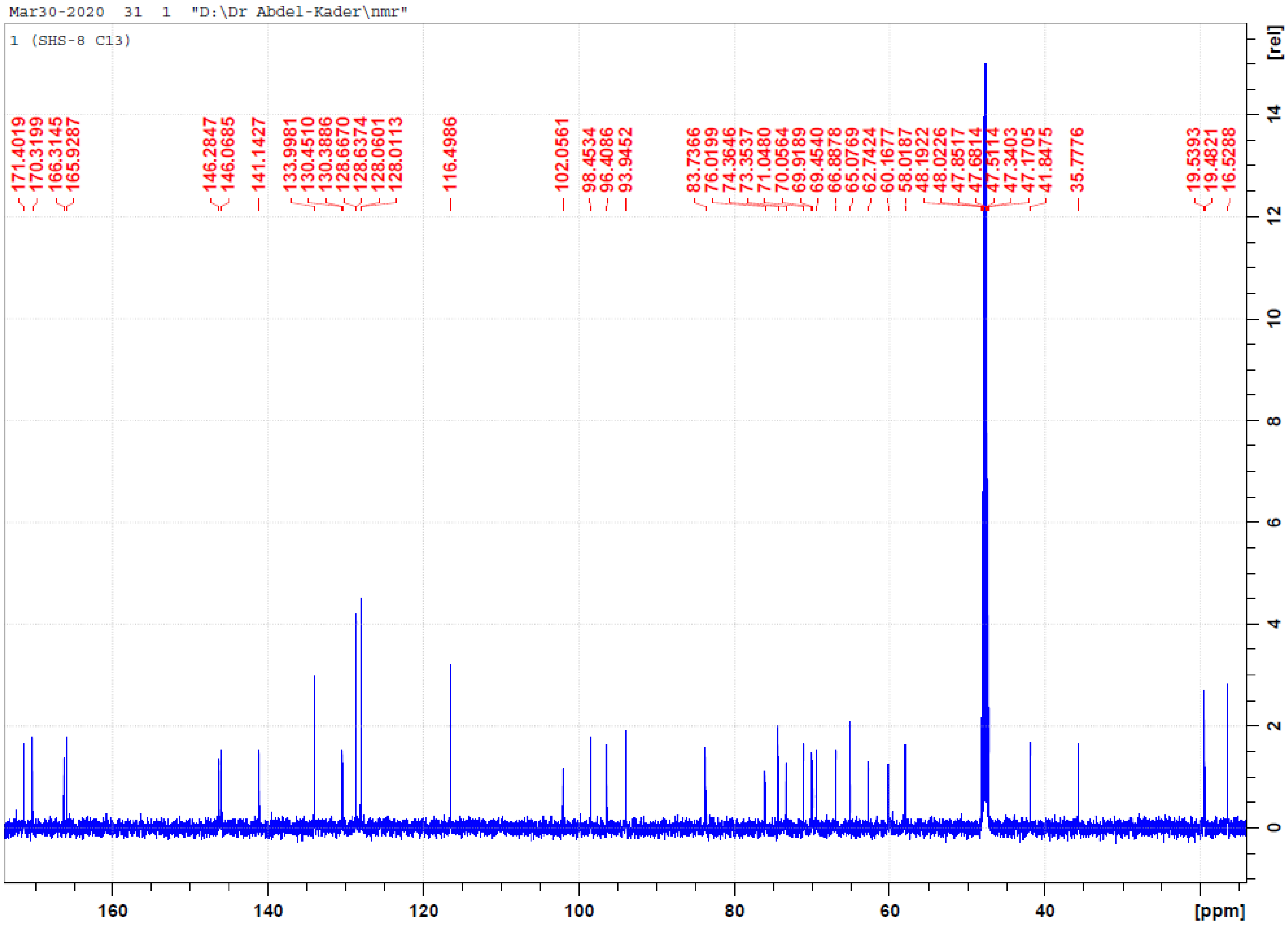


**Figure S5.** $^{13}$CNMR spectrum of compound 1.

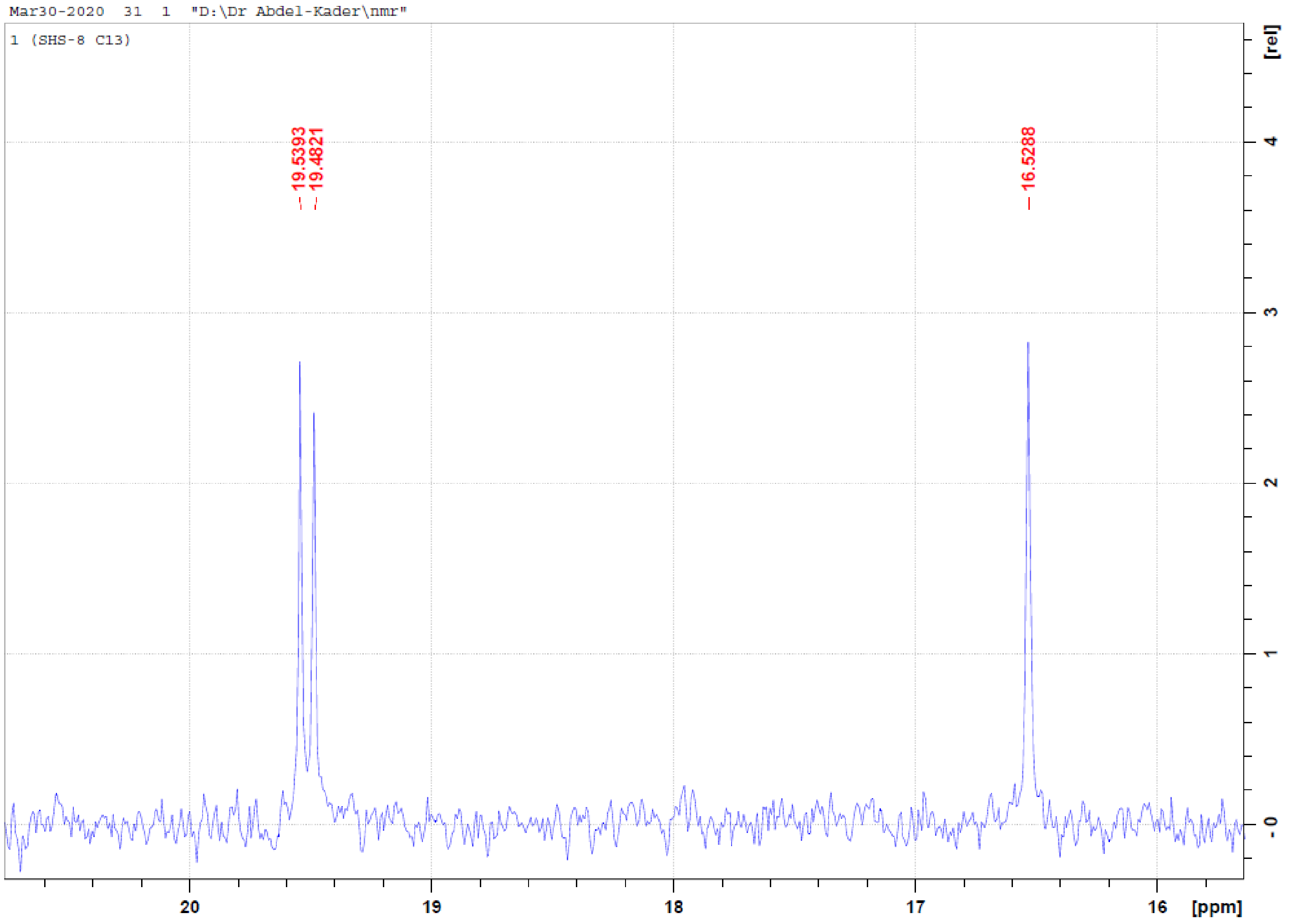


**Figure S6.** $^{13}$CNMR spectrum of compound 1 (Expansion).

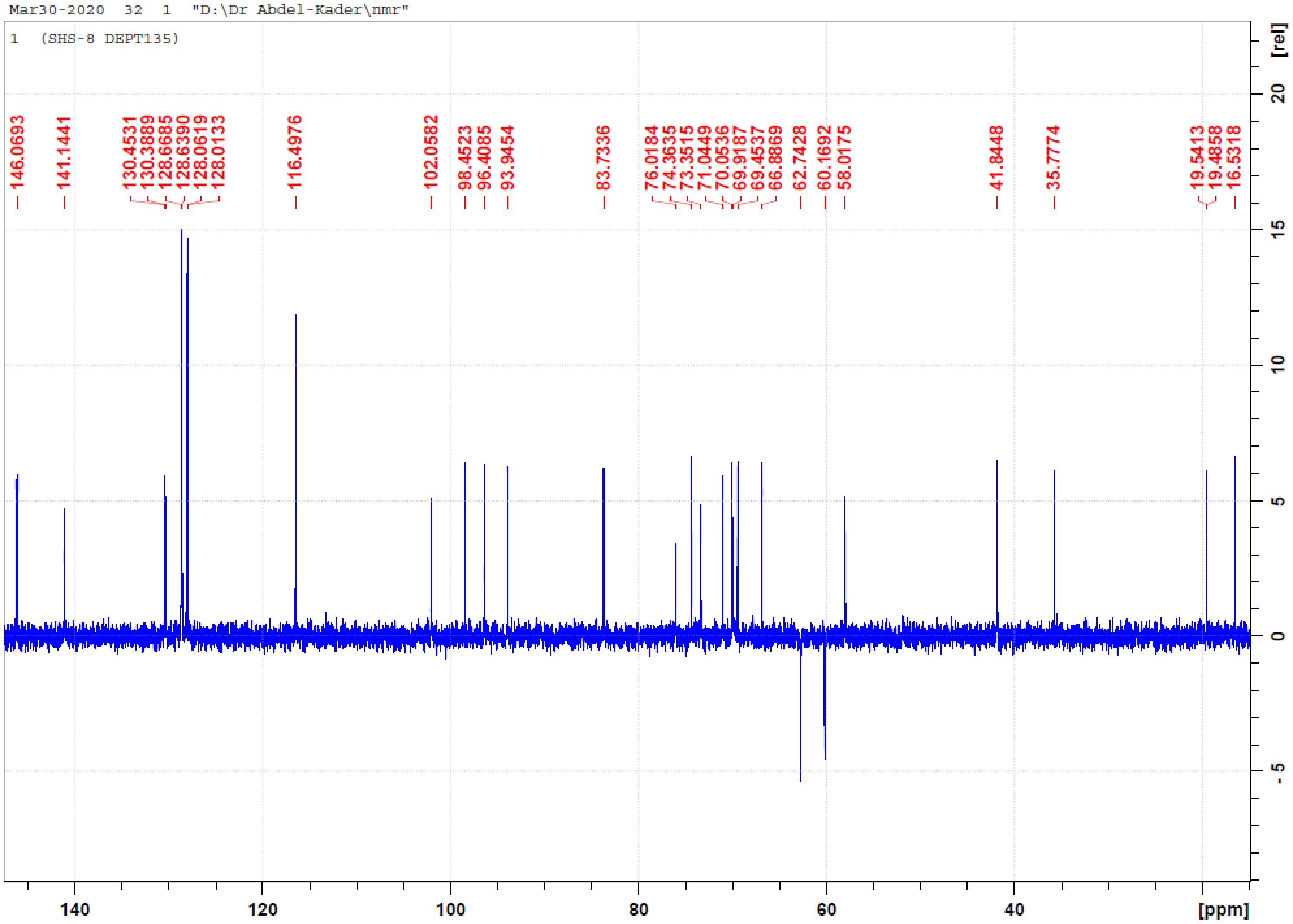


**Figure S7.** DEPT135 spectrum of compound 1.

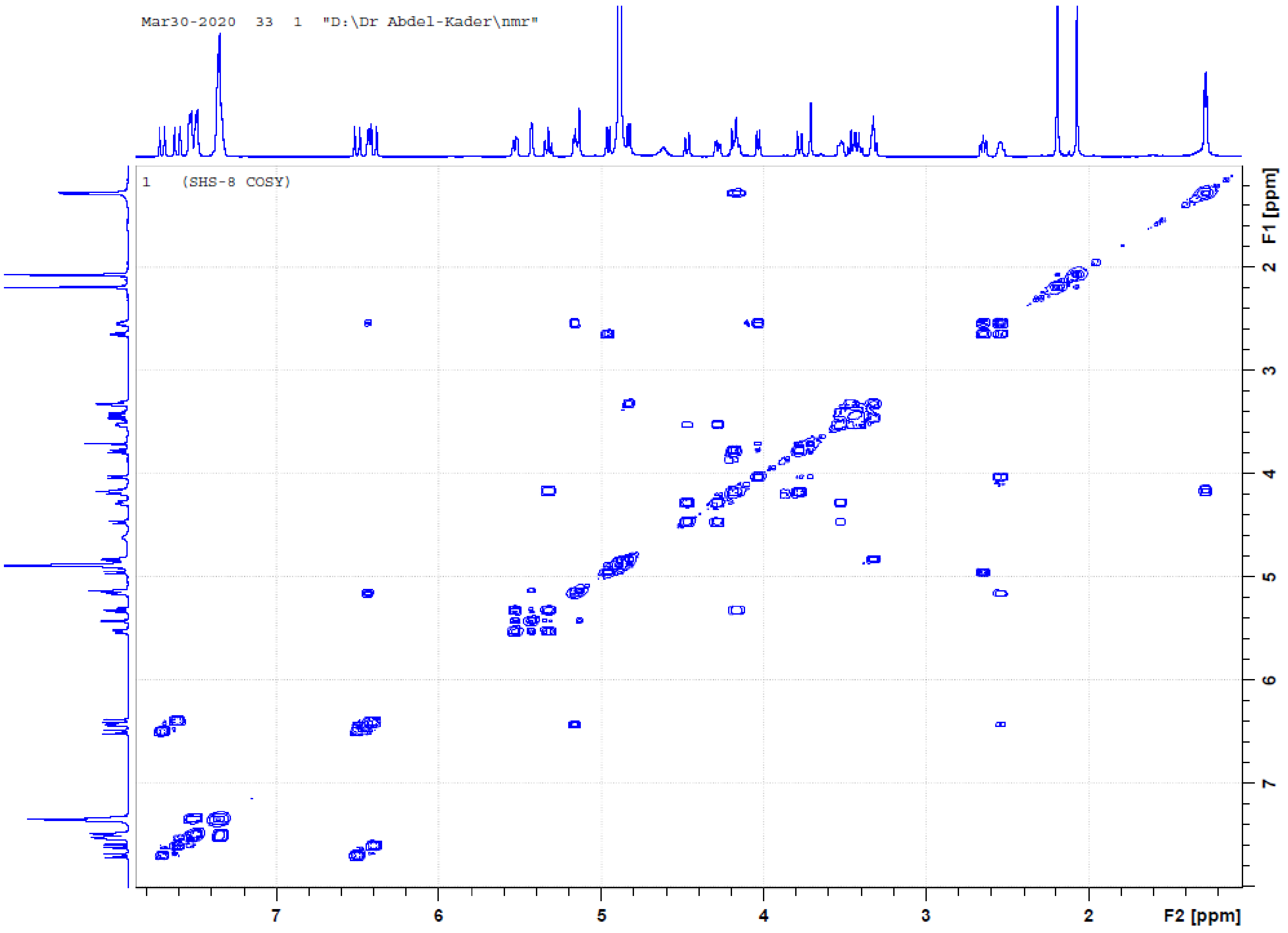


**Figure S8.** COSY spectrum of compound 1.

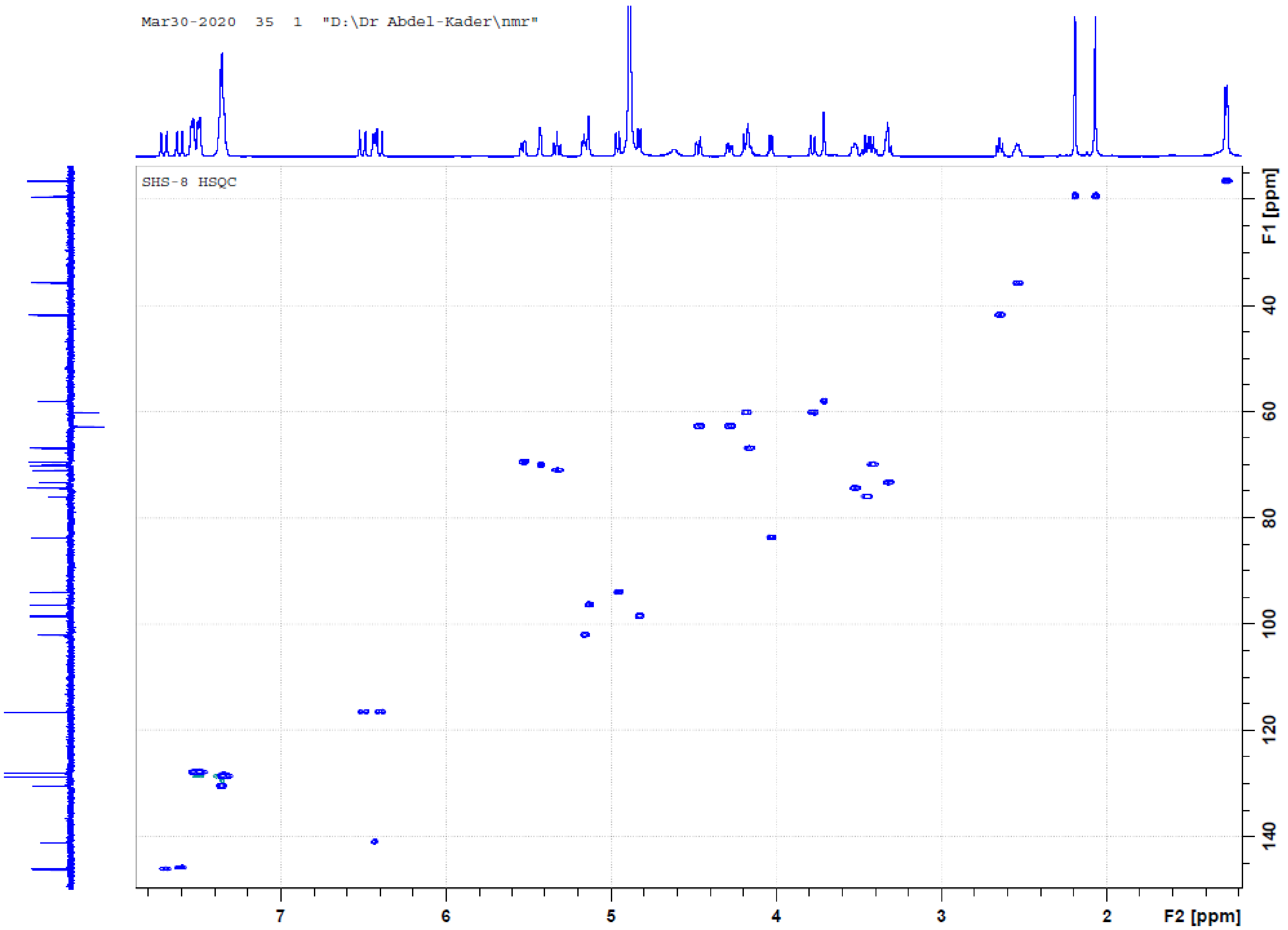


**Figure S9.** HSQC spectrum of compound 1.

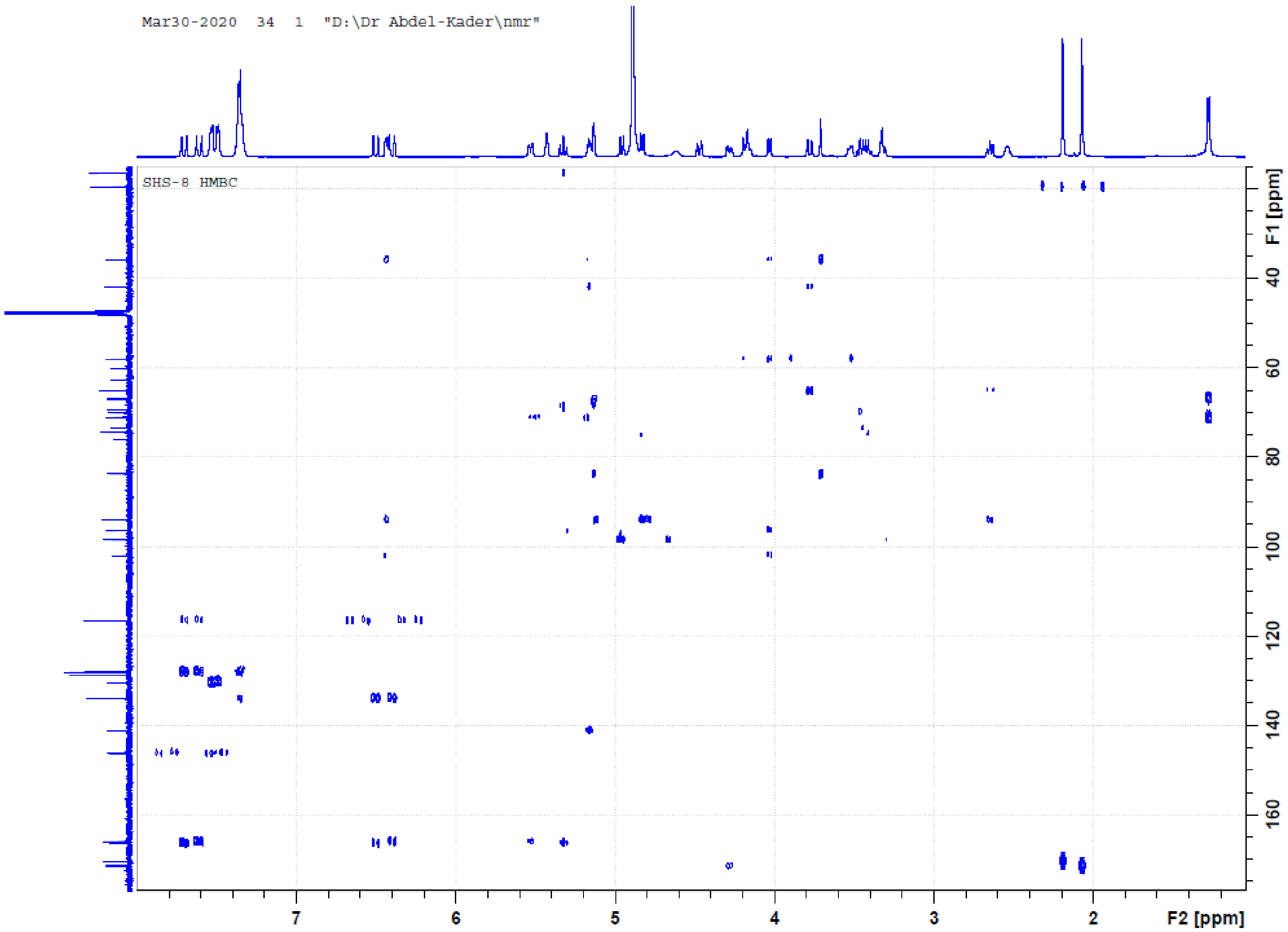


**Figure S10.** HMBC spectrum of compound 1.

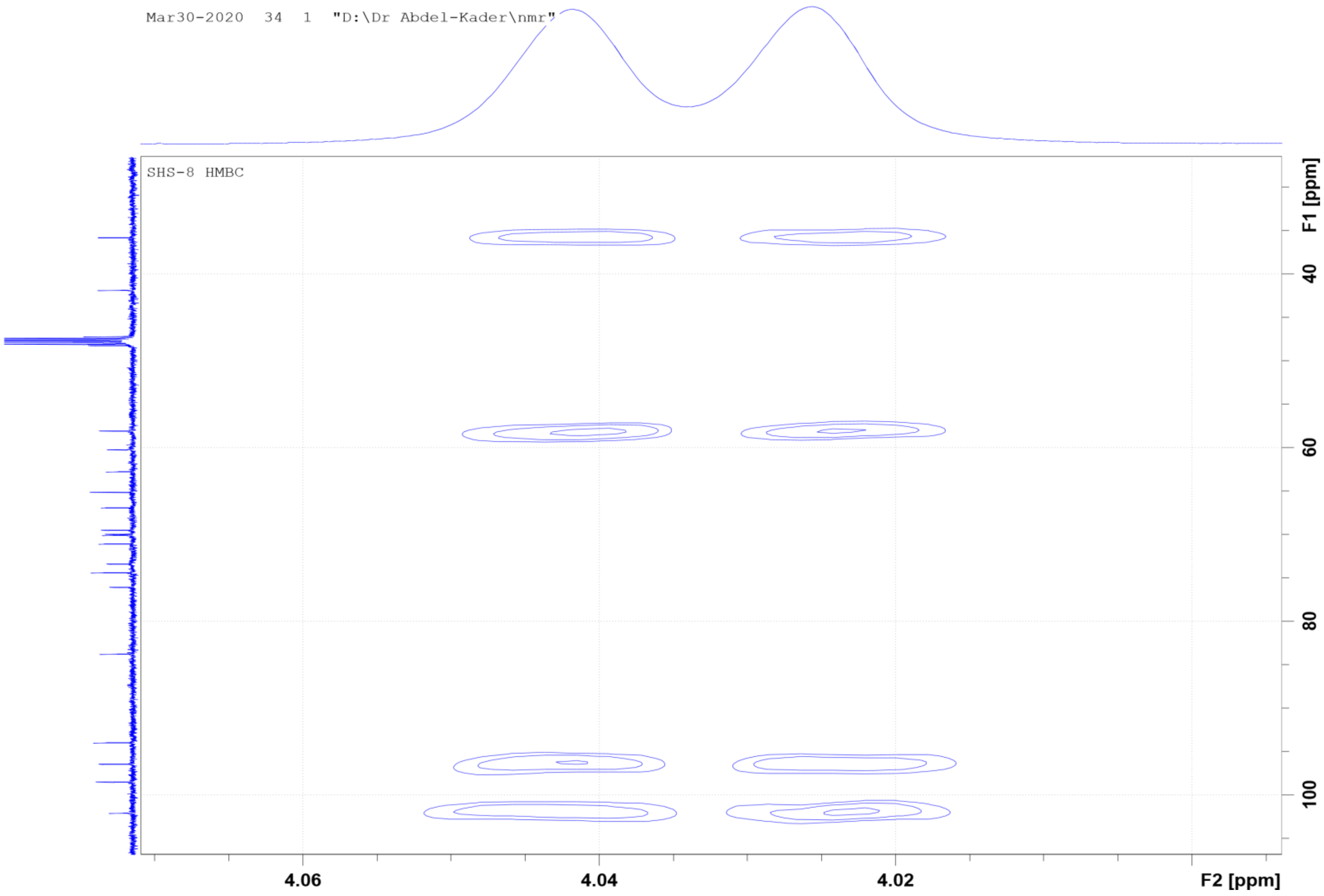


**Figure S11.** HMBC spectrum of compound 1 (Expansion).

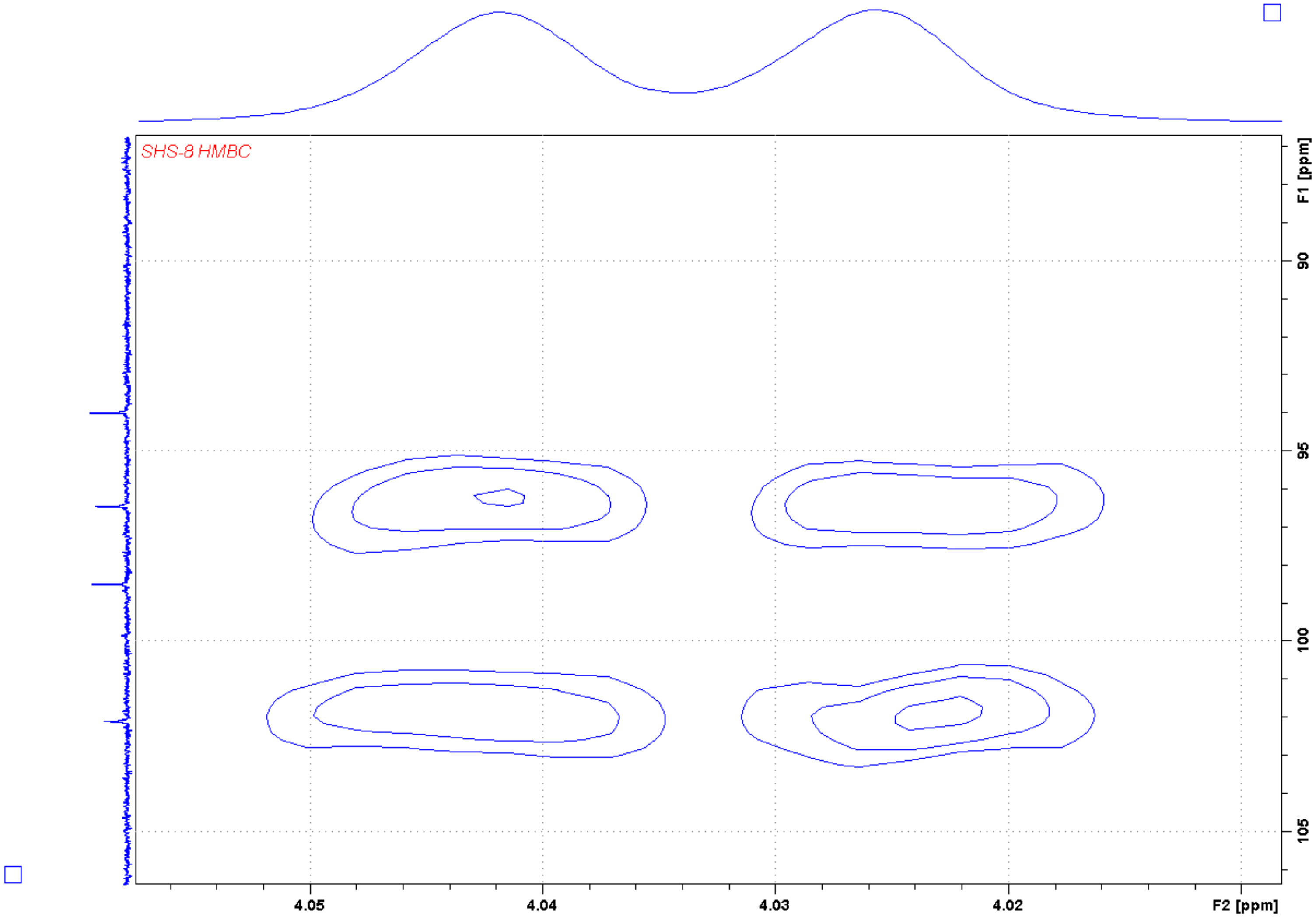


**Figure S12.** HMBC spectrum of compound 1 (Expansion).

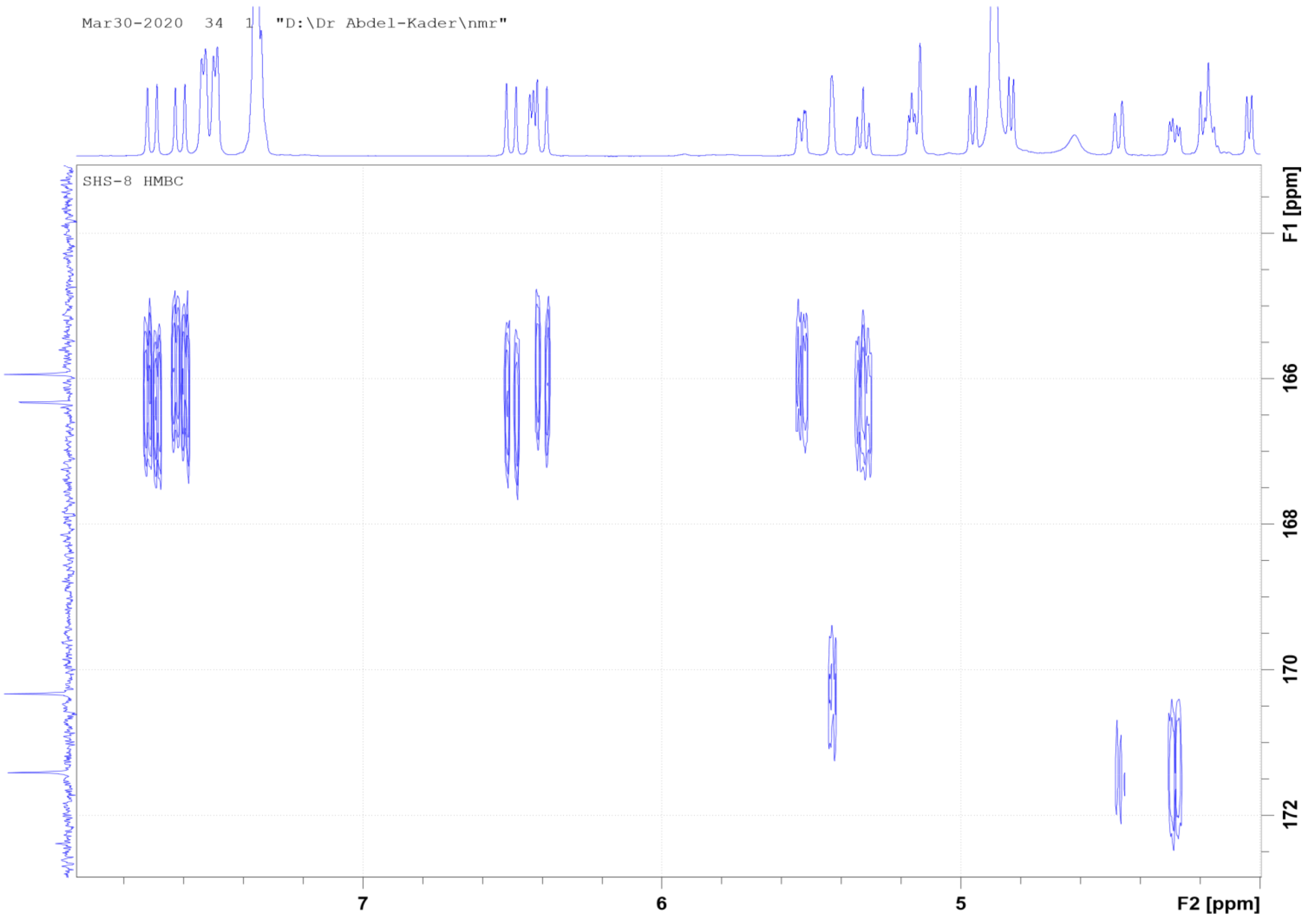


**Figure S13.** HMBC spectrum of compound 1 (Expansion).

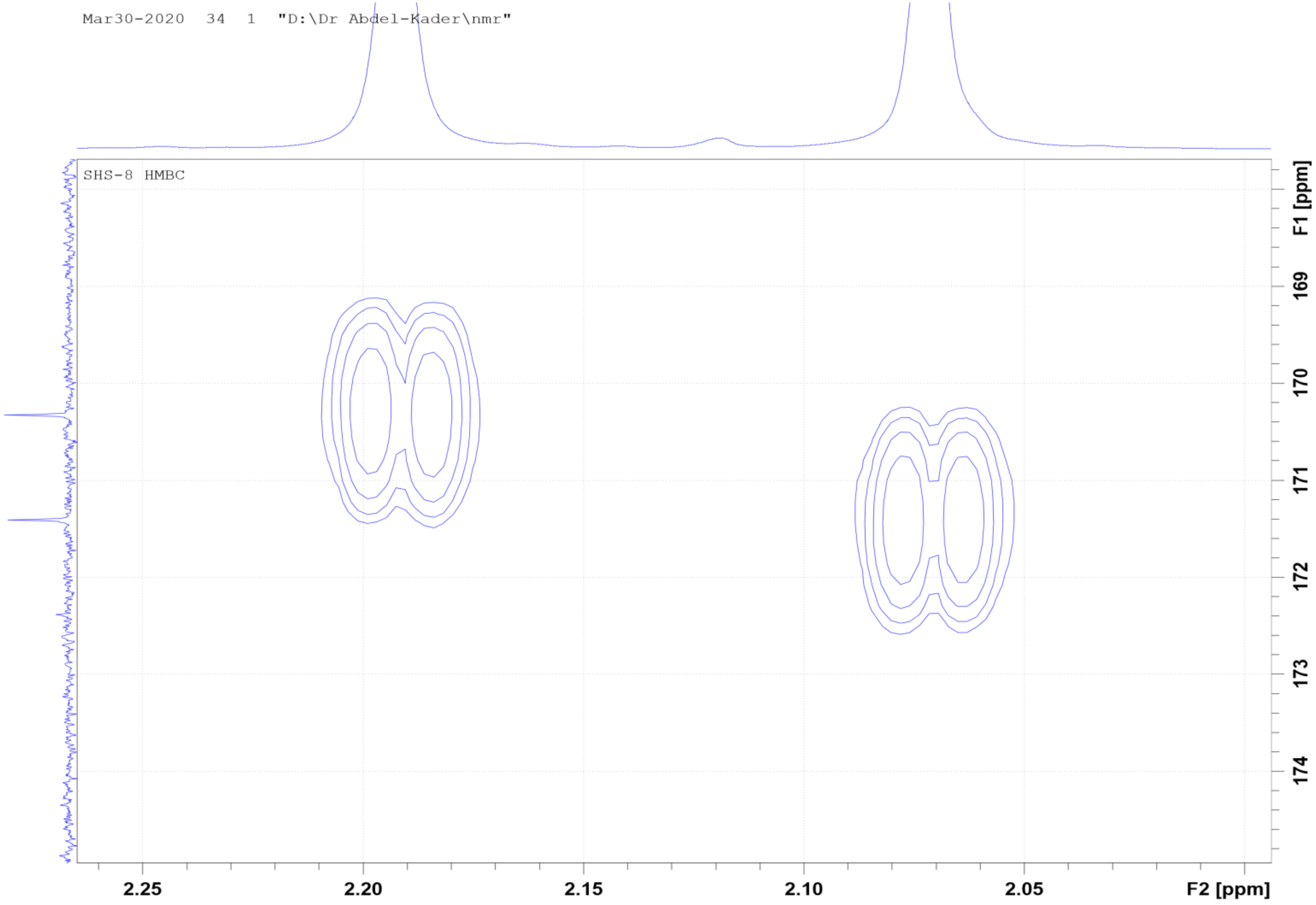


**Figure S14.** HMBC spectrum of compound 1 (Expansion).

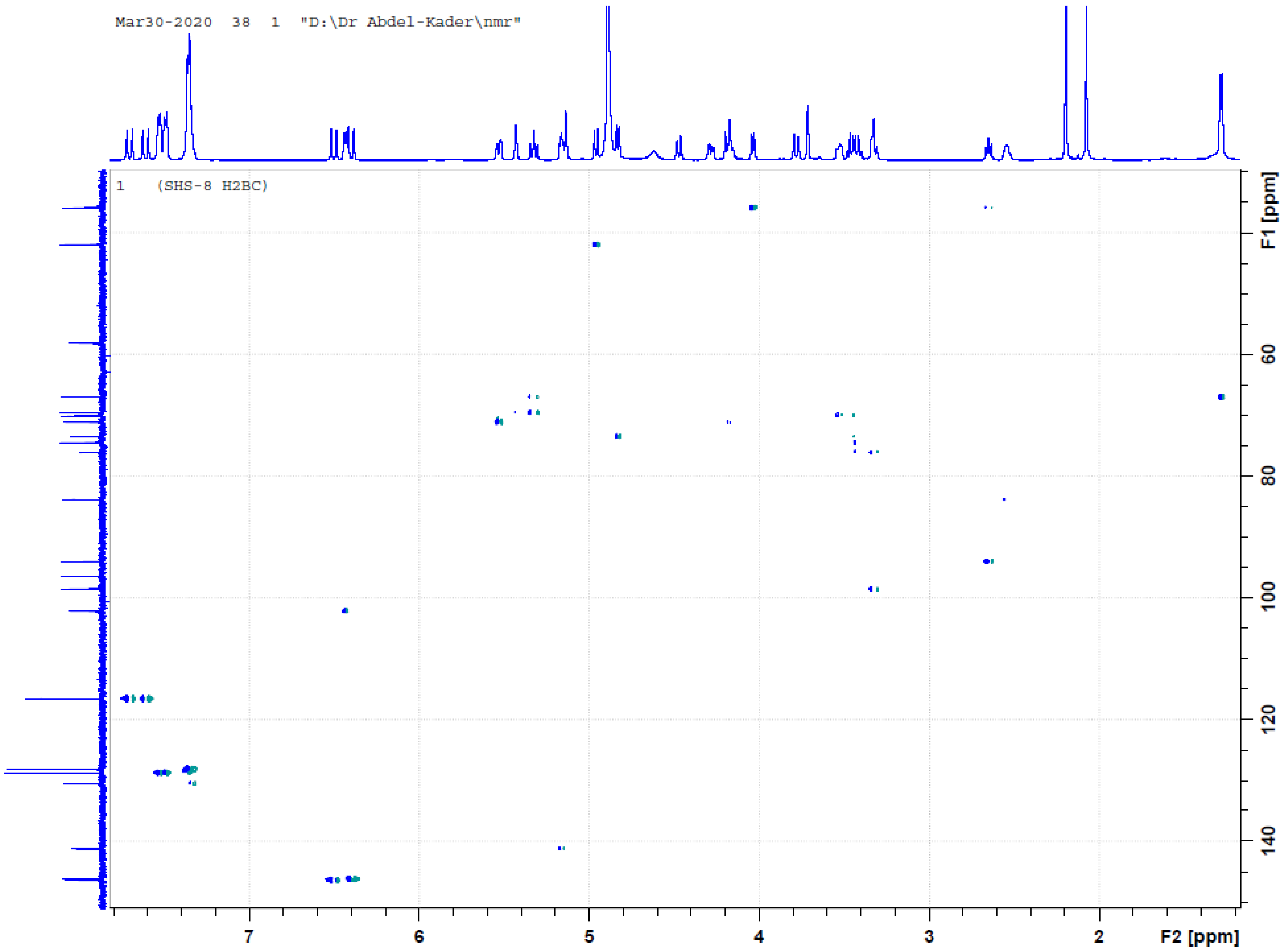


**Figure S15.** H2BC spectrum of compound 1.

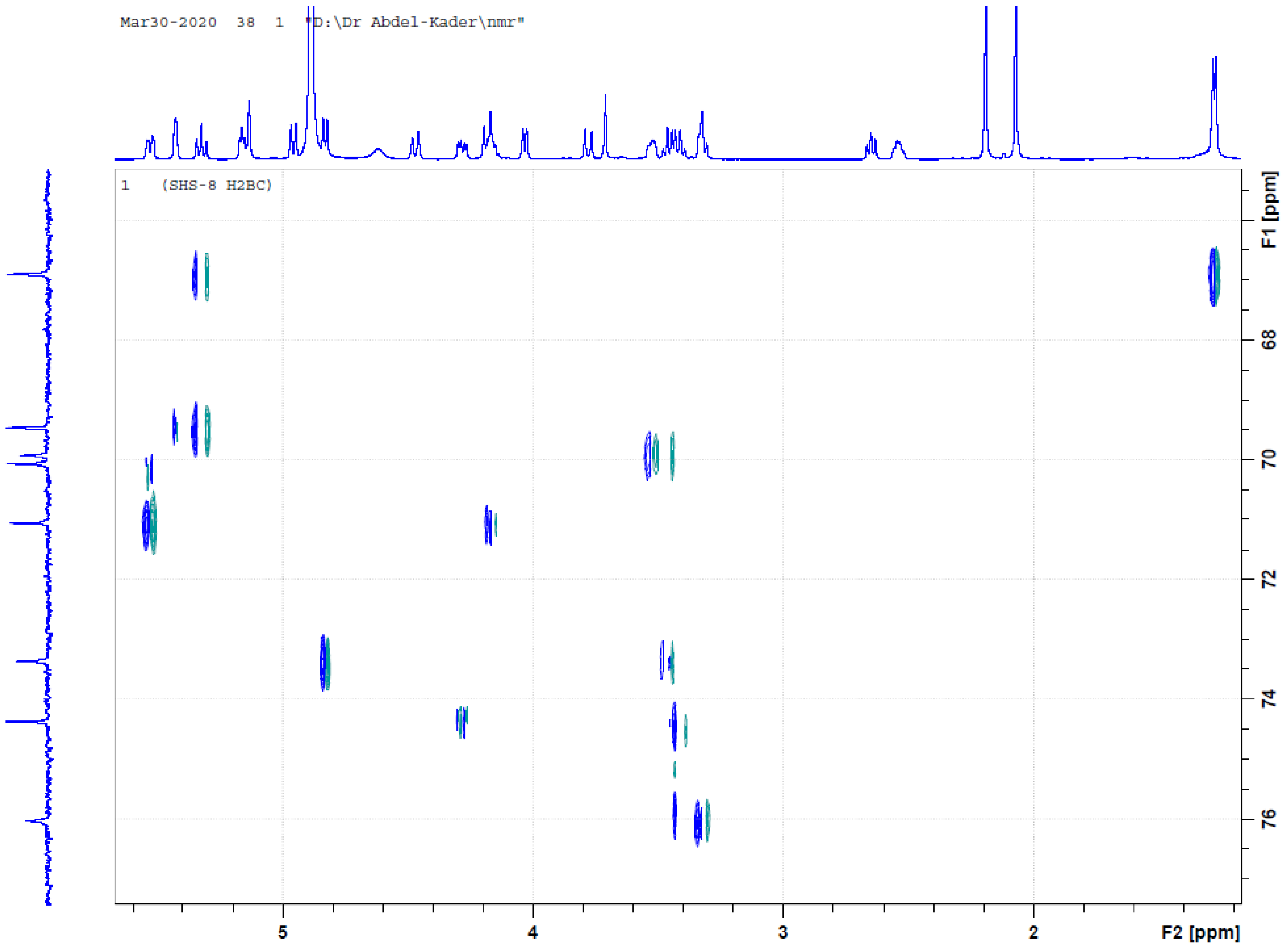


**Figure S16.** H2BC spectrum of compound 1 (Expansion).

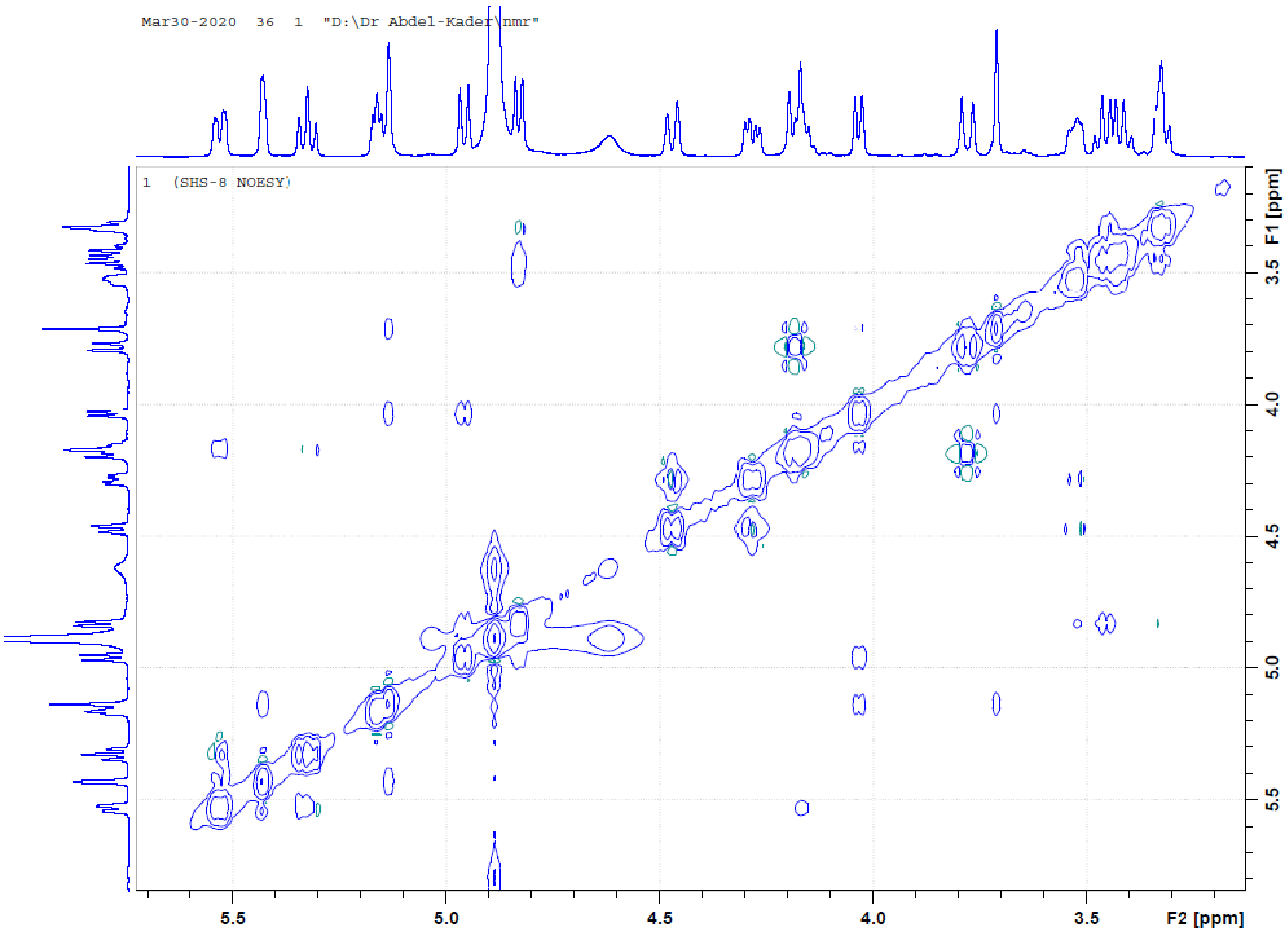


**Figure S17.** NOESY spectrum of compound 1 (Expansion).

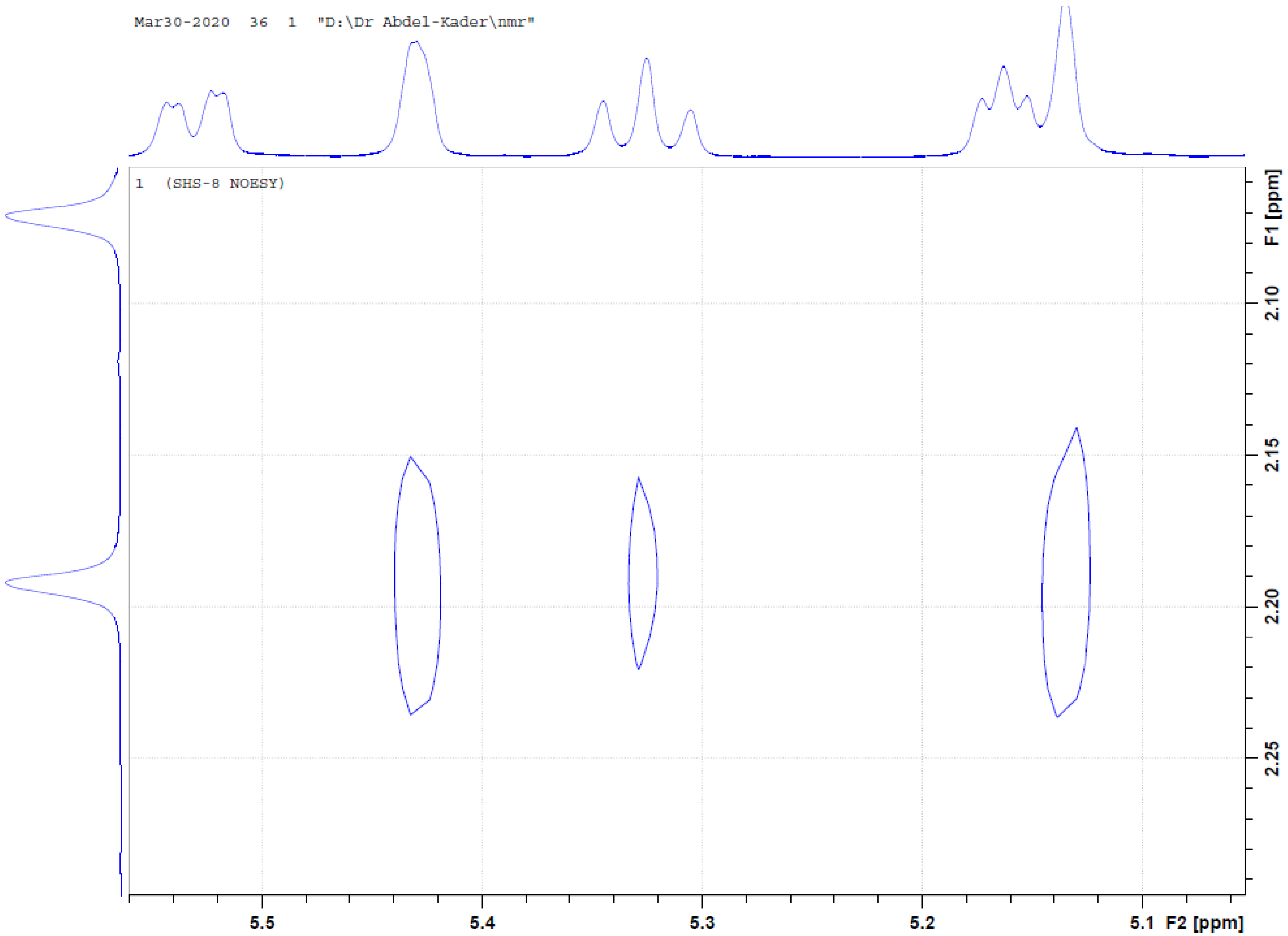


**Figure S18.** NOESY spectrum of compound 1 (Expansion).

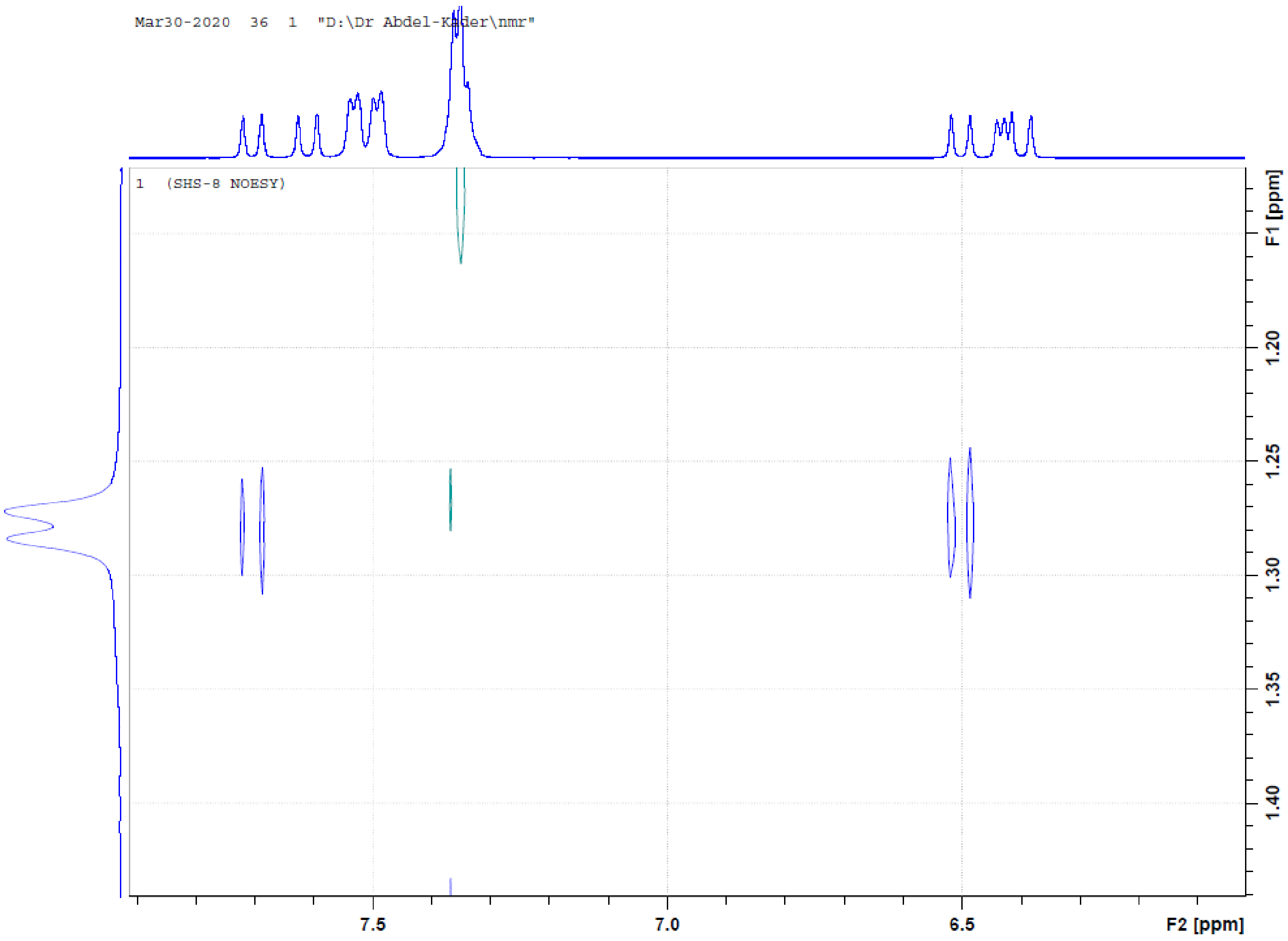


**Figure S19.** NOESY spectrum of compound 1 (Expansion).

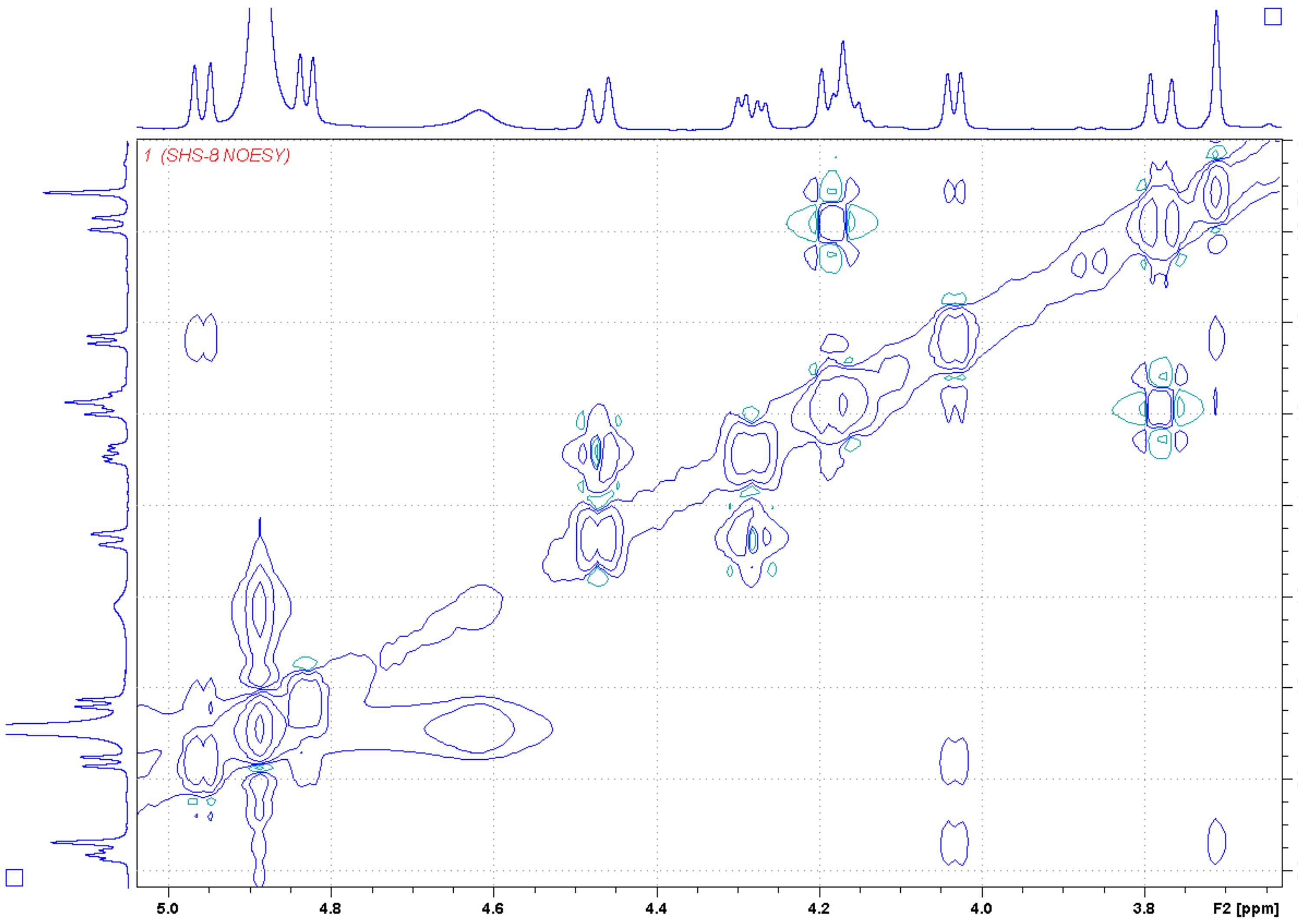


**Figure S20.** NOESY spectrum of compound 1 (Expansion).

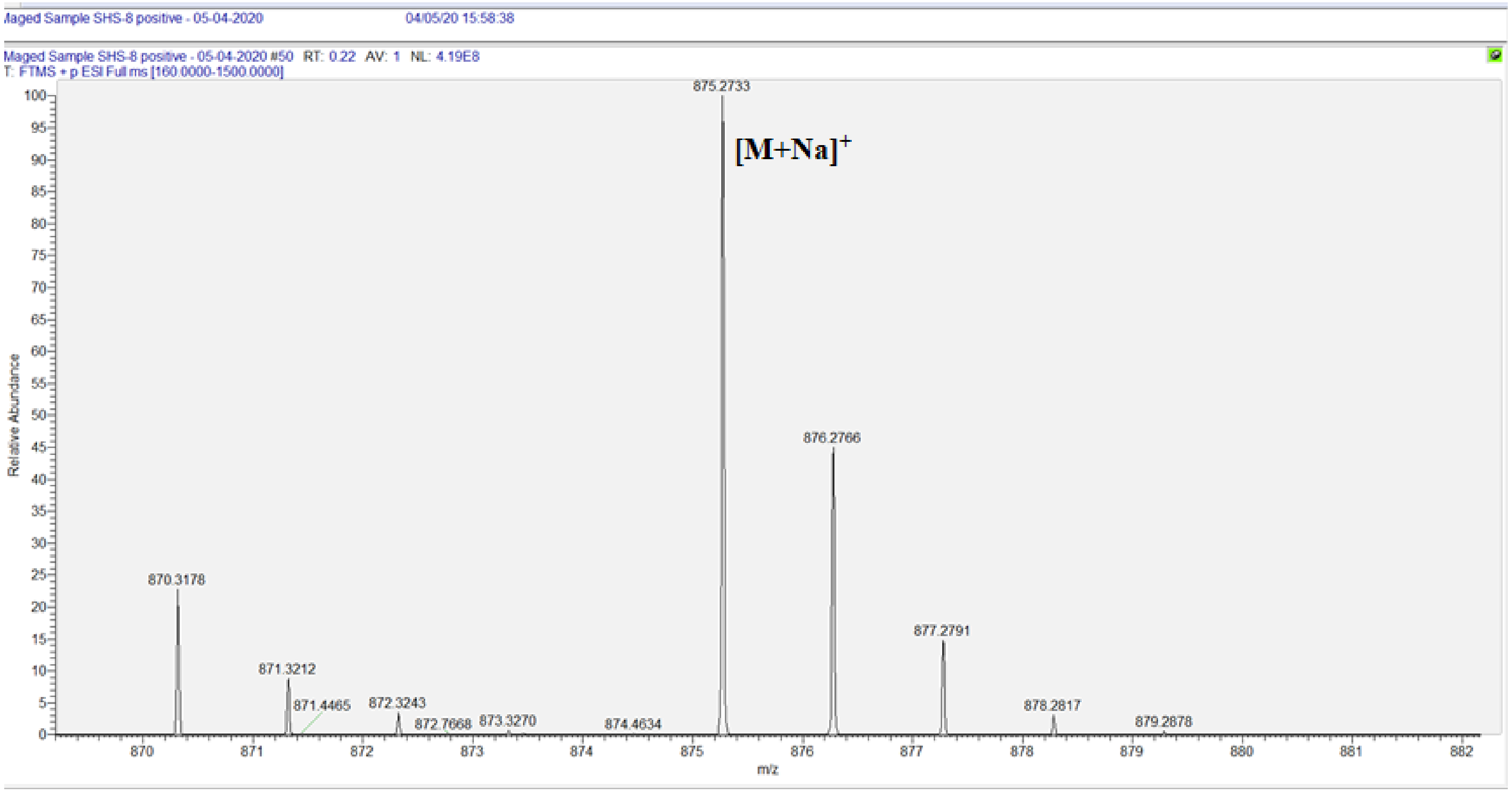


**Figure S21.** HRESIMS of compound 1 (positive mode).

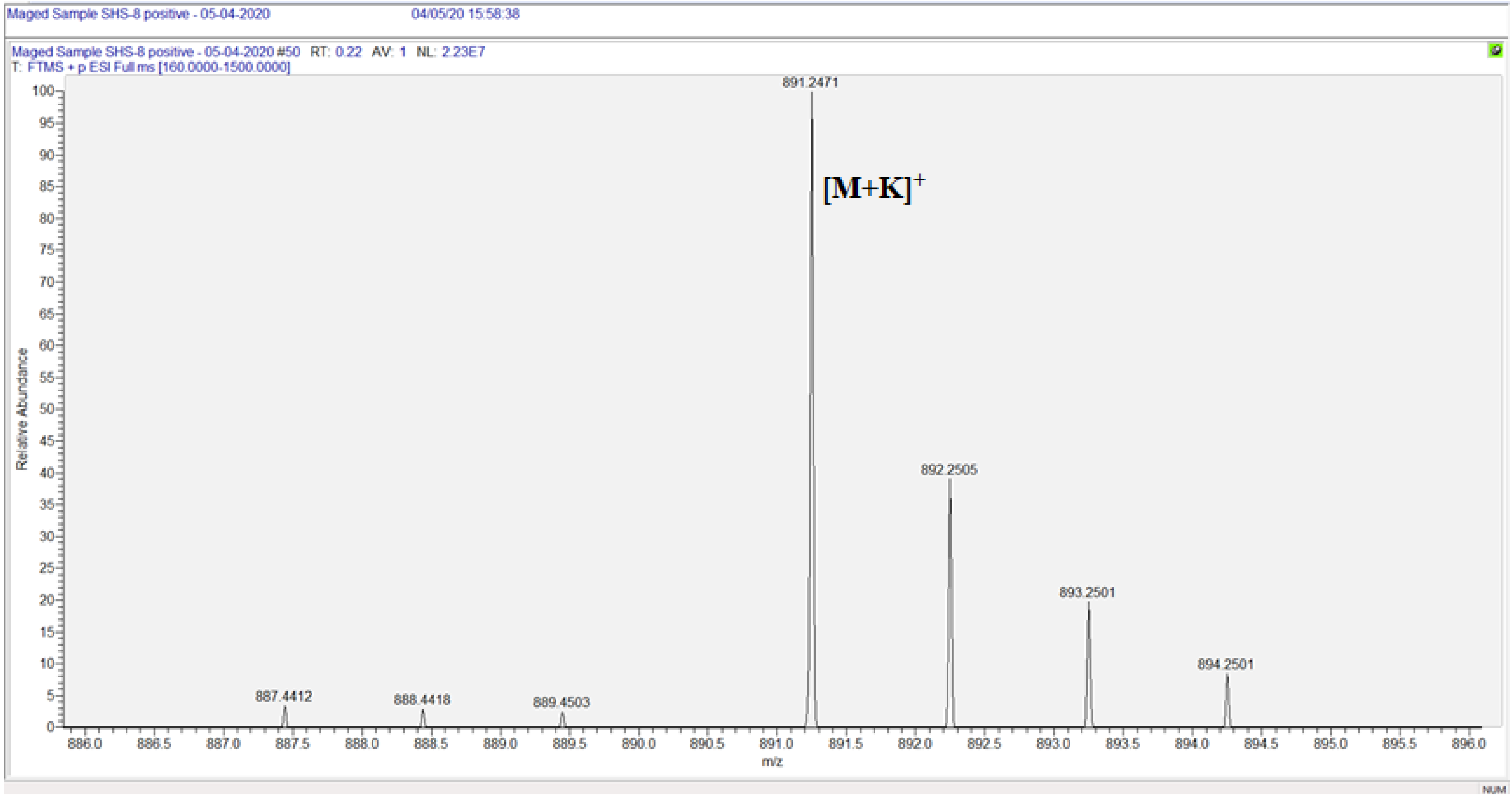


**Figure S22.** HRESIMS of compound 1 (positive mode).

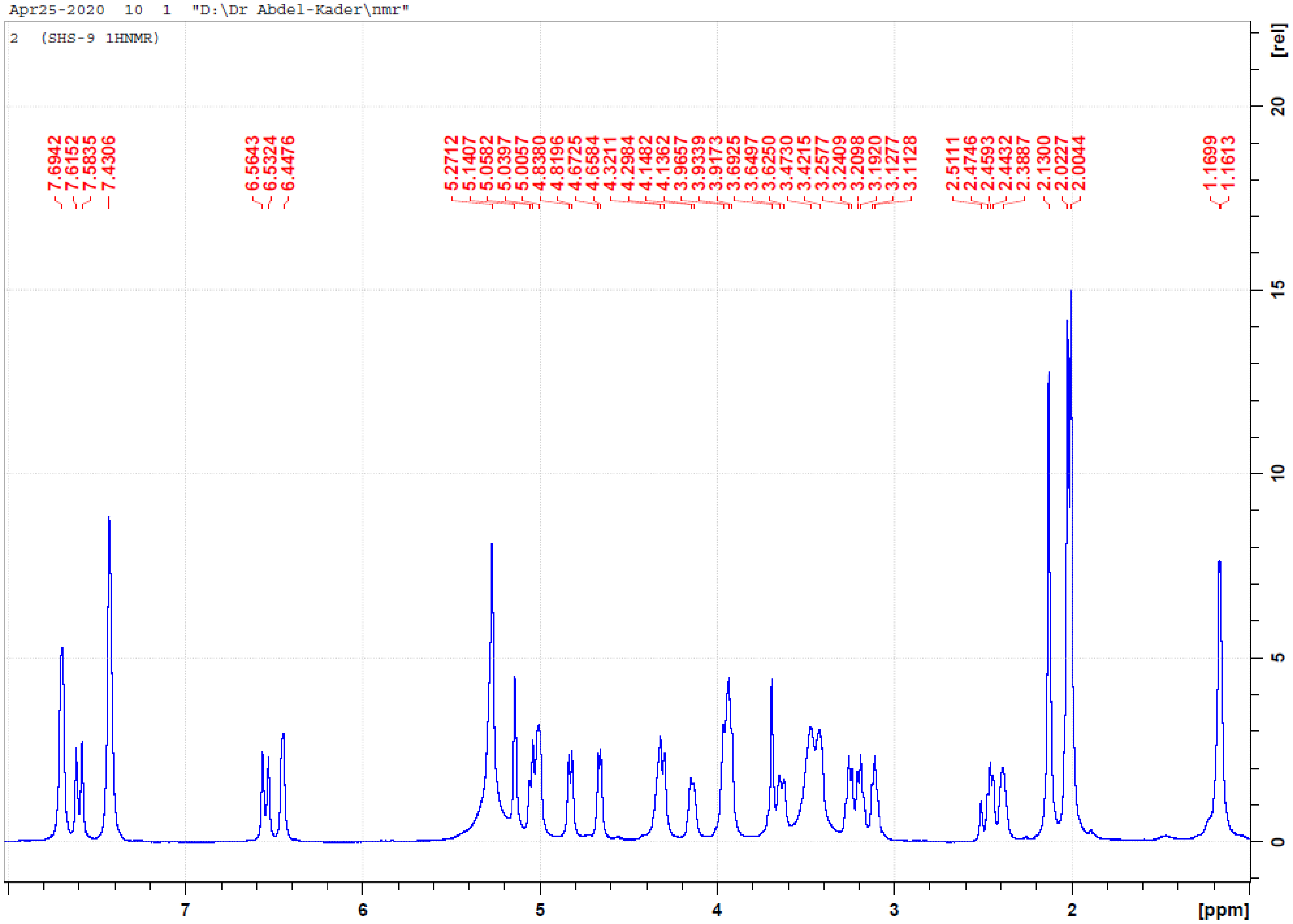


**Figure S23.** $^{1}$HNMR spectrum of compound 2.

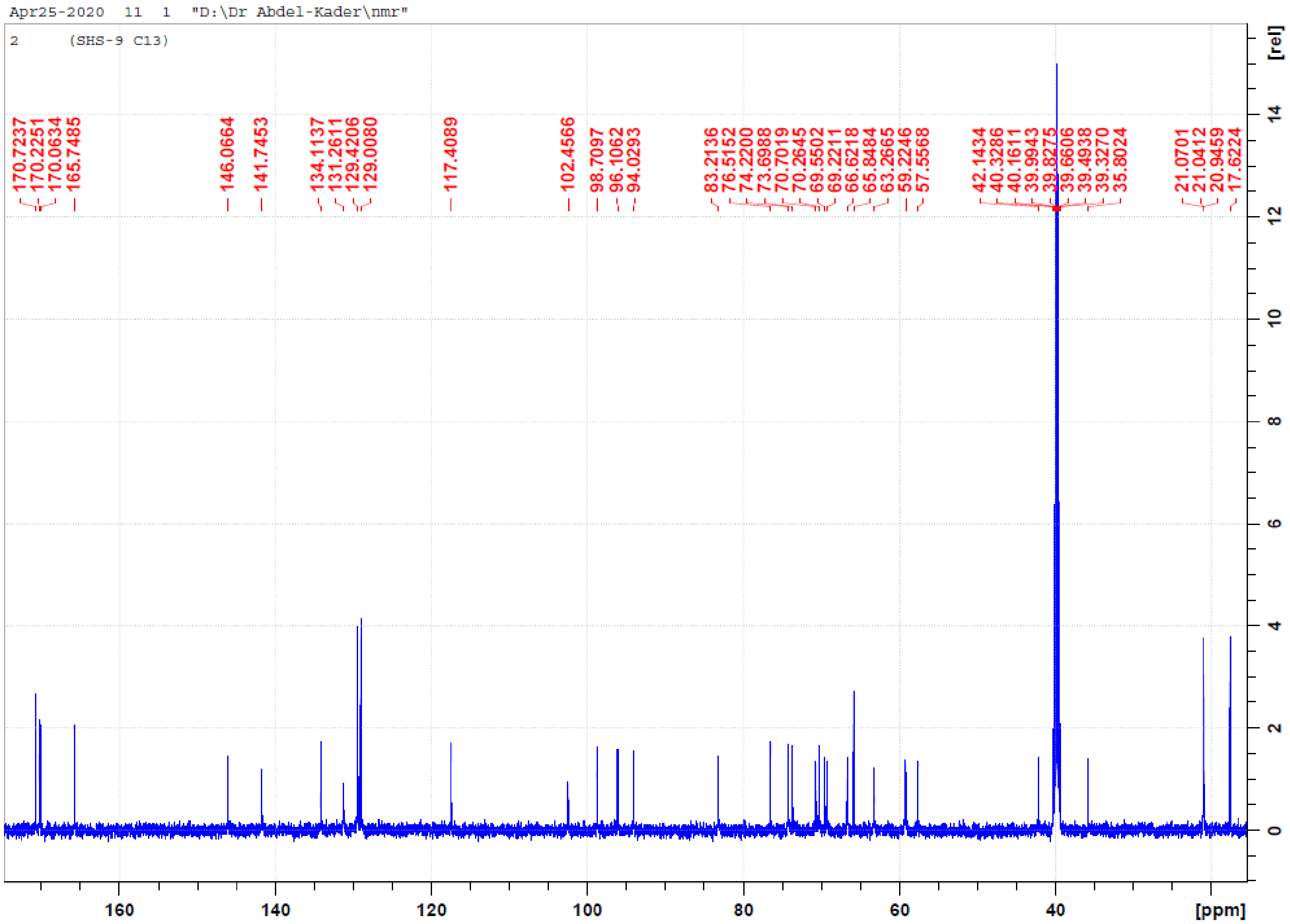


**Figure S24.** $^{13}$CNMR spectrum of compound 2.

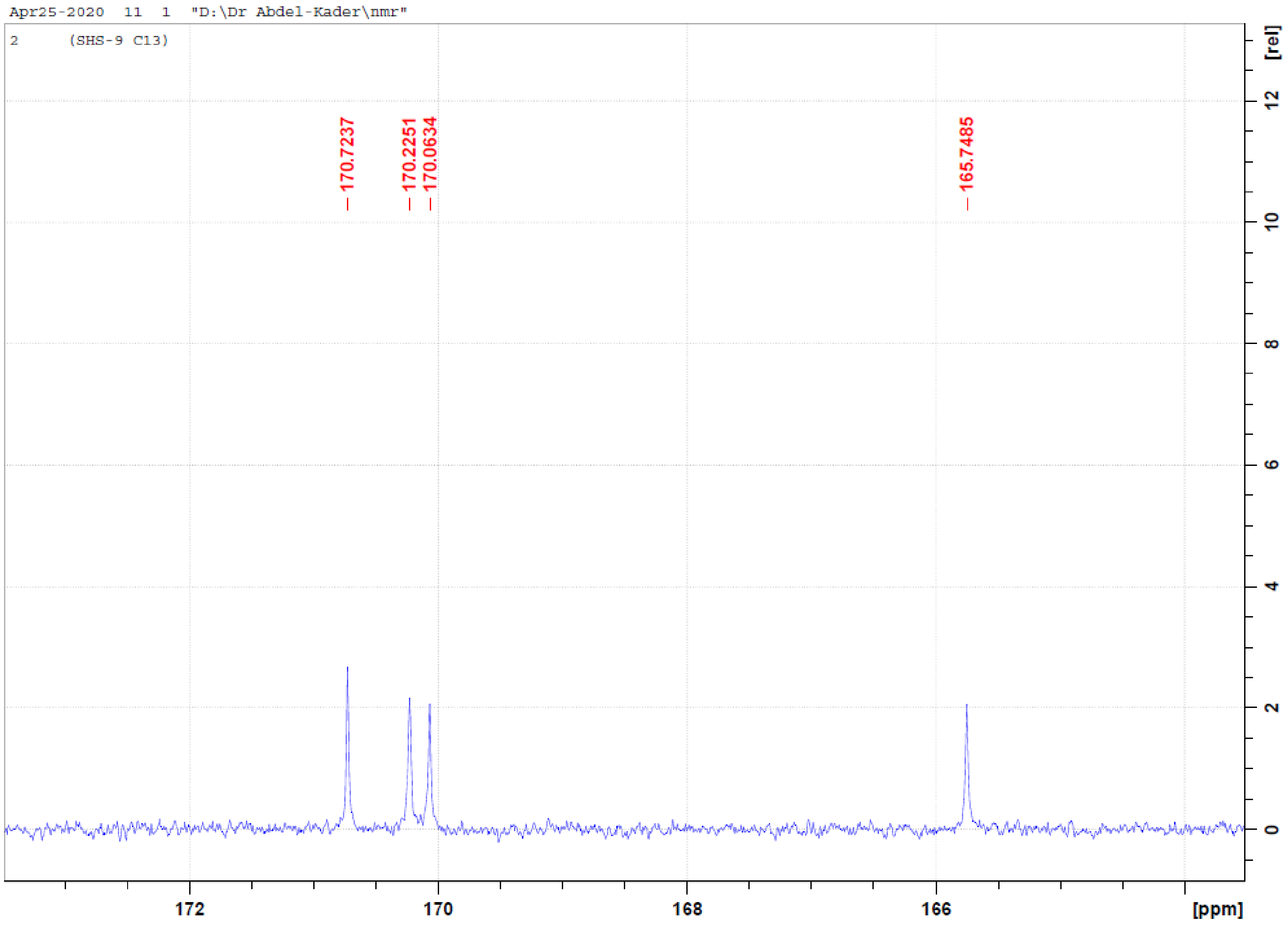


**Figure S25.** $^{13}$CNMR spectrum of compound 2 (Expansion).

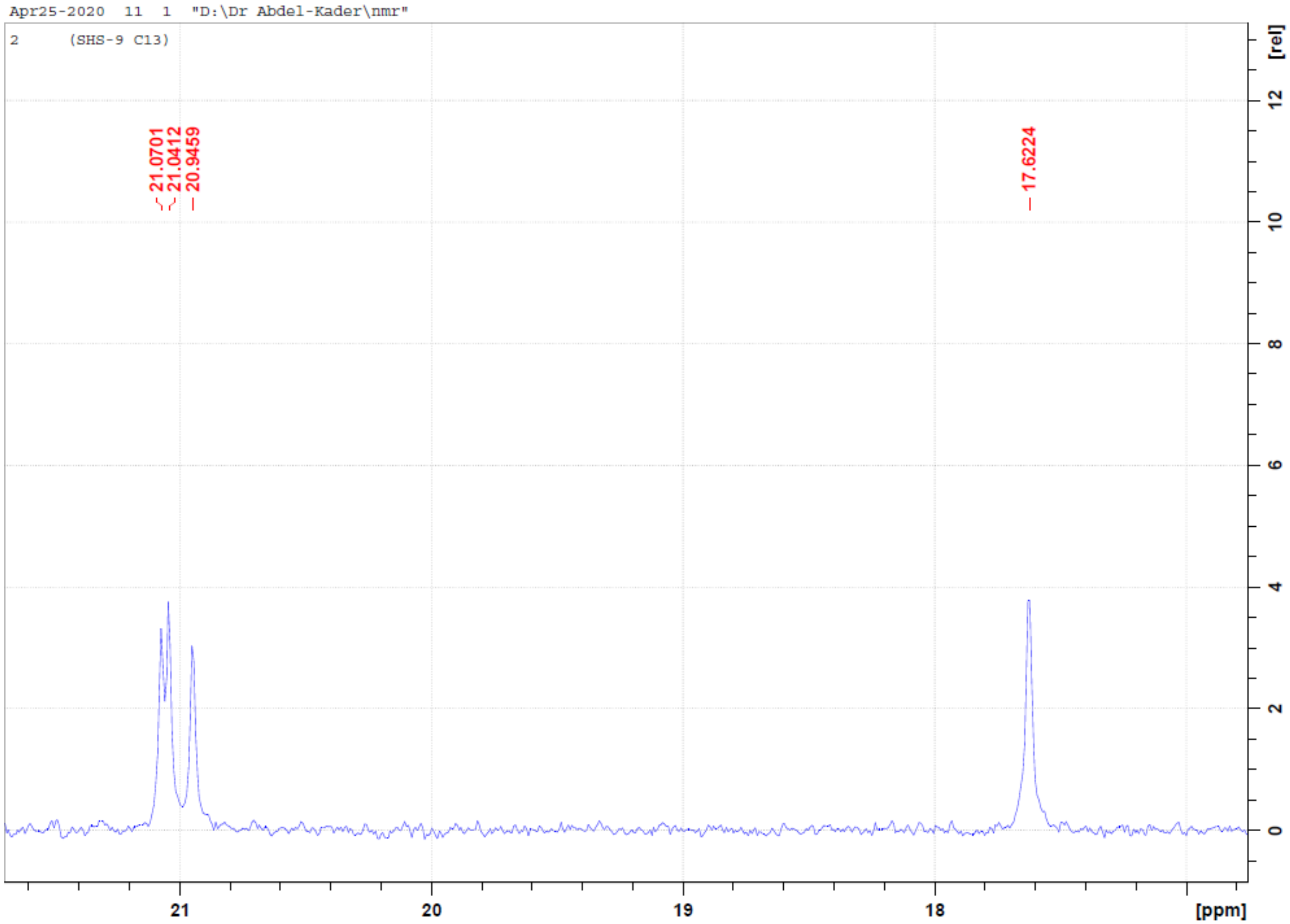


**Figure S26.** $^{13}$CNMR spectrum of compound 2 (Expansion).

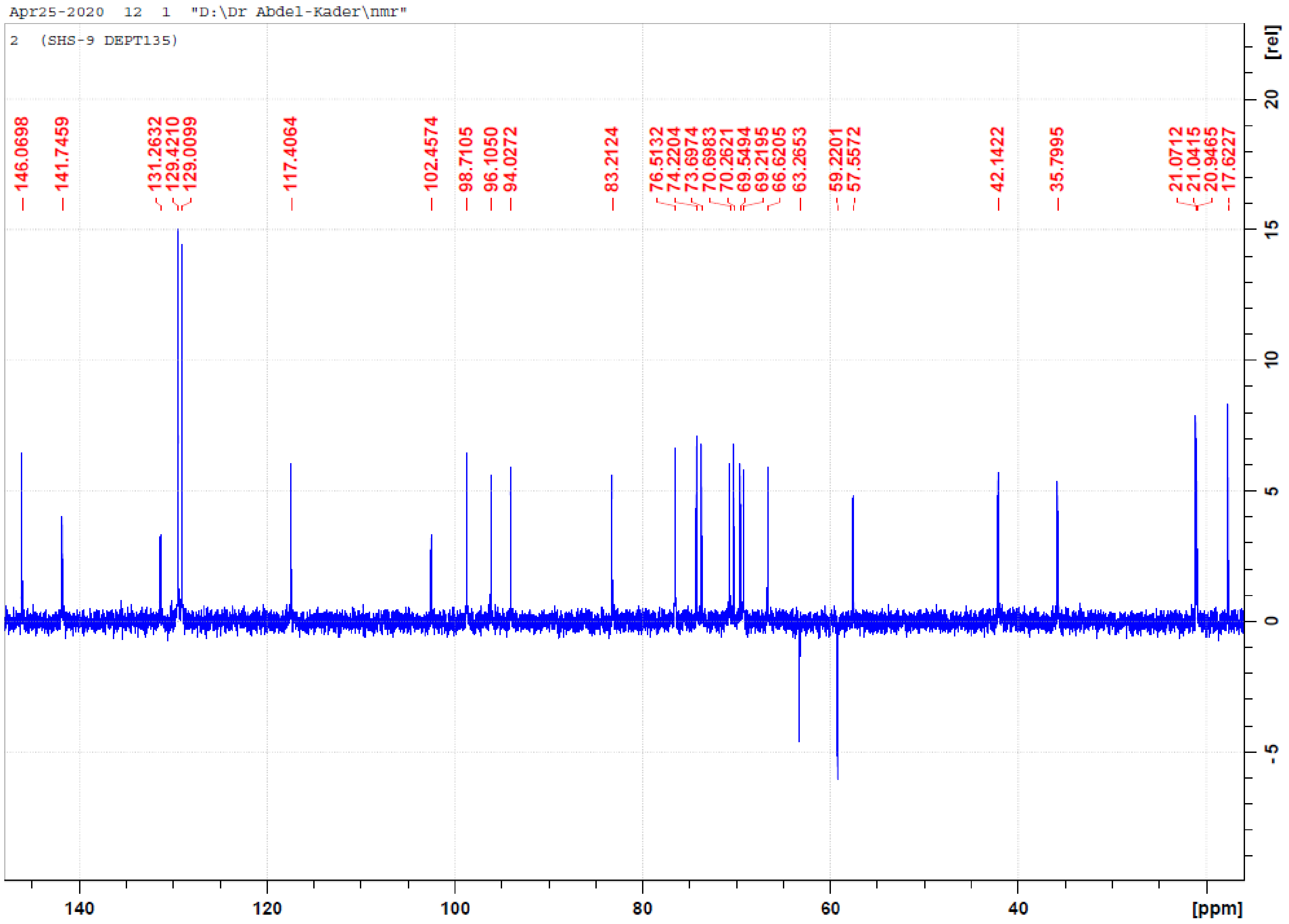


**Figure S27.** DEPT135 spectrum of compound 2.

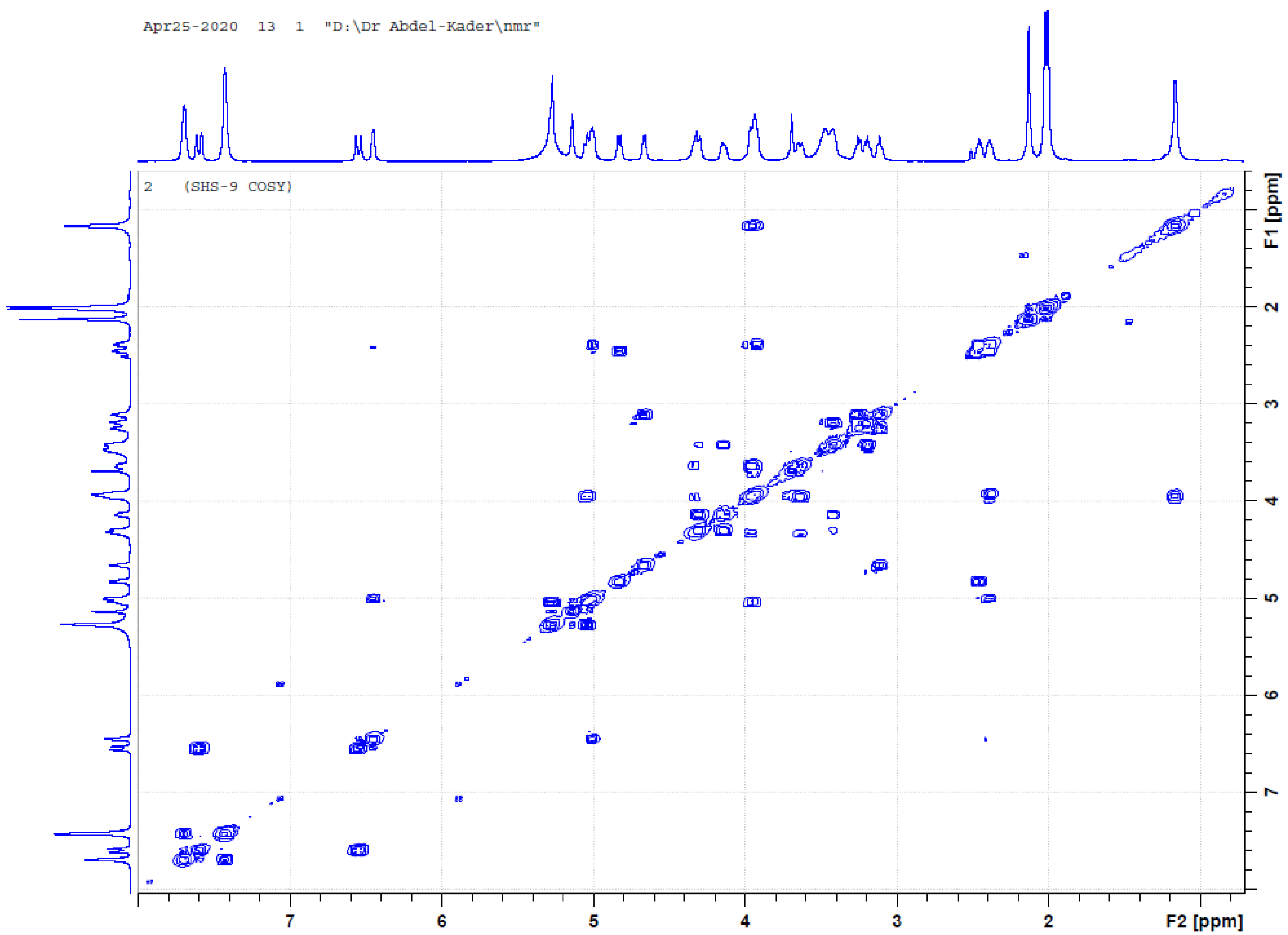


**Figure S28.** COSY spectrum of compound 2.

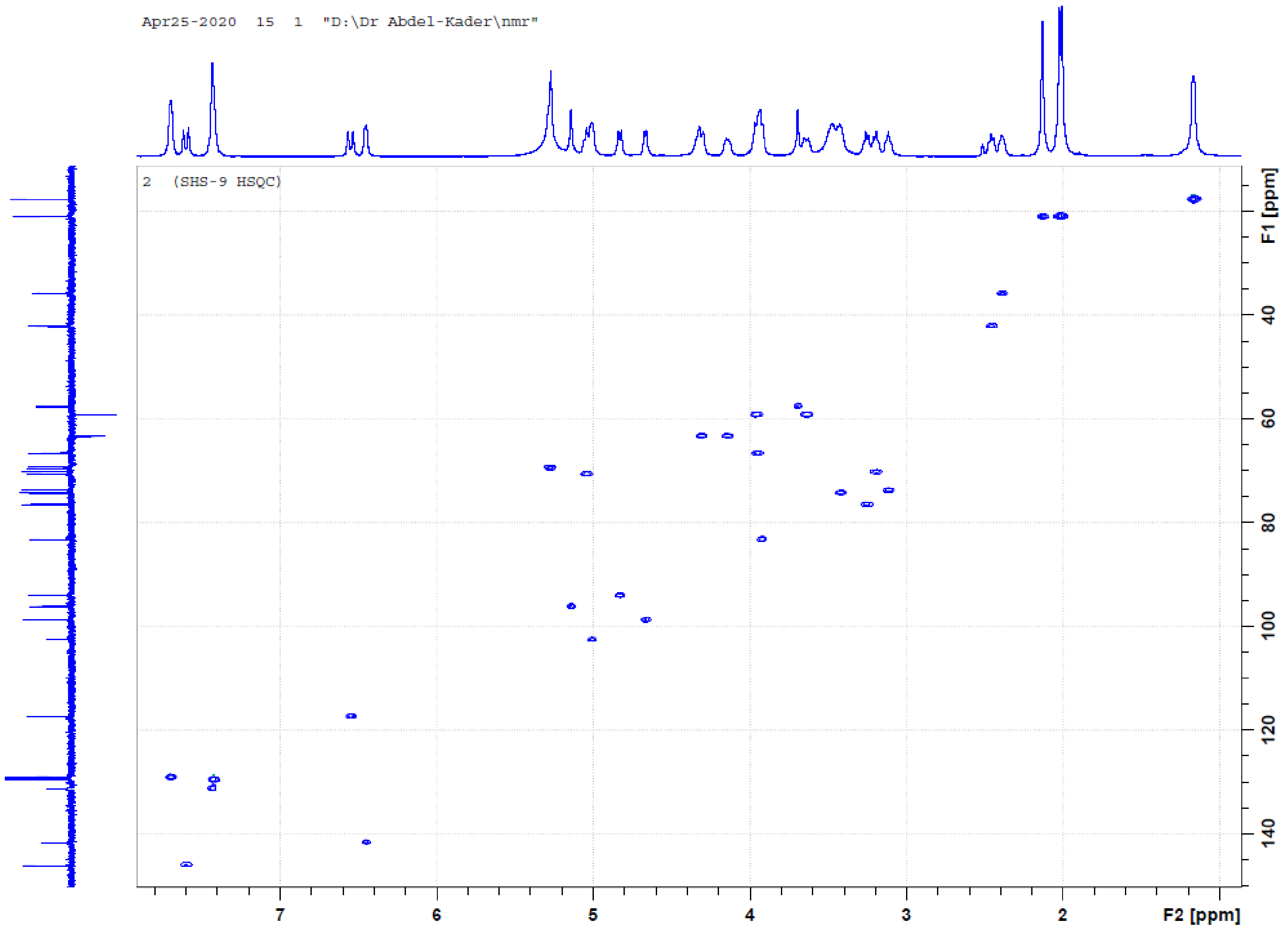


**Figure S29.** HSQC spectrum of compound 2.

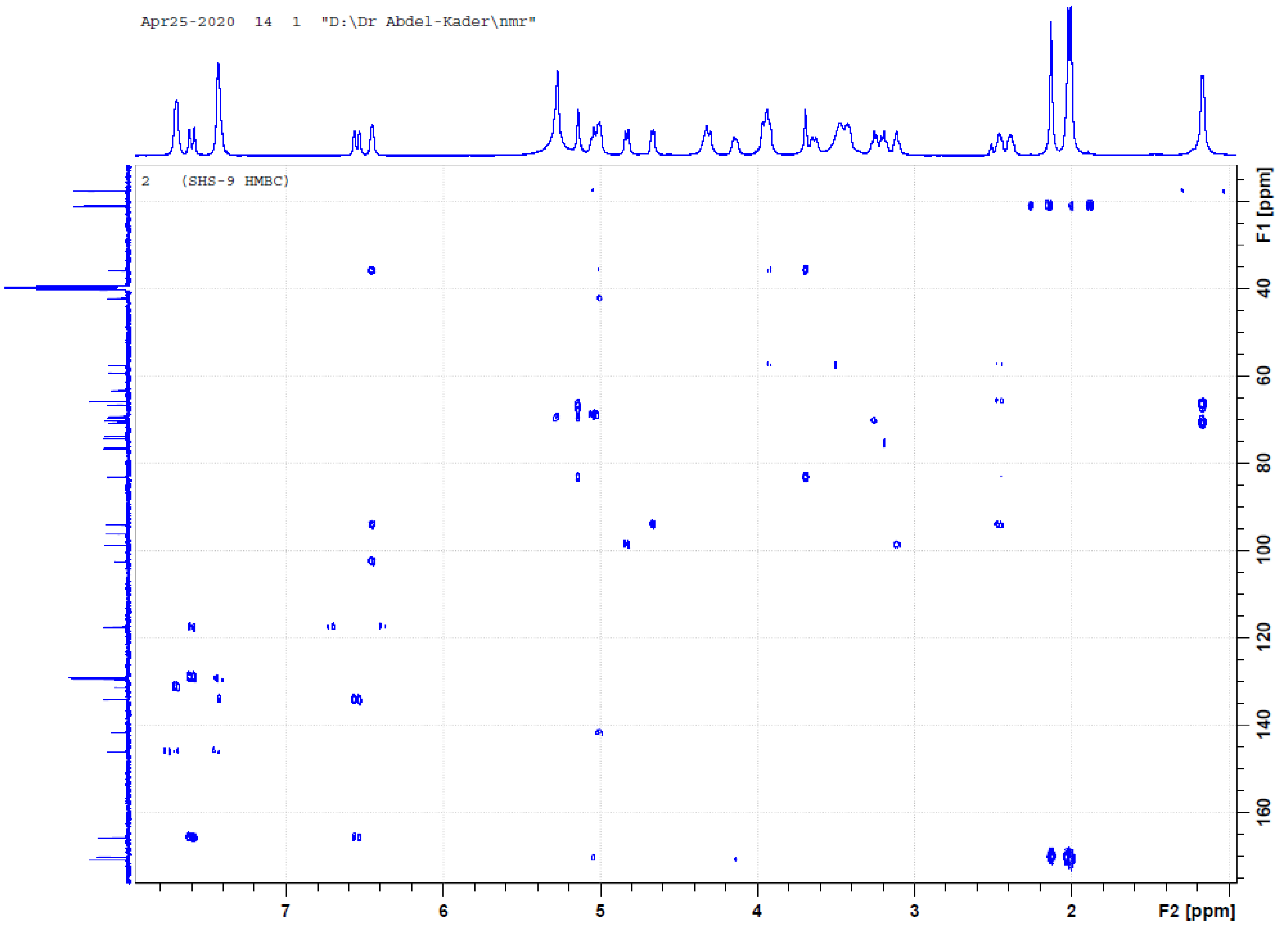


**Figure S30.** HMBC spectrum of compound 2.

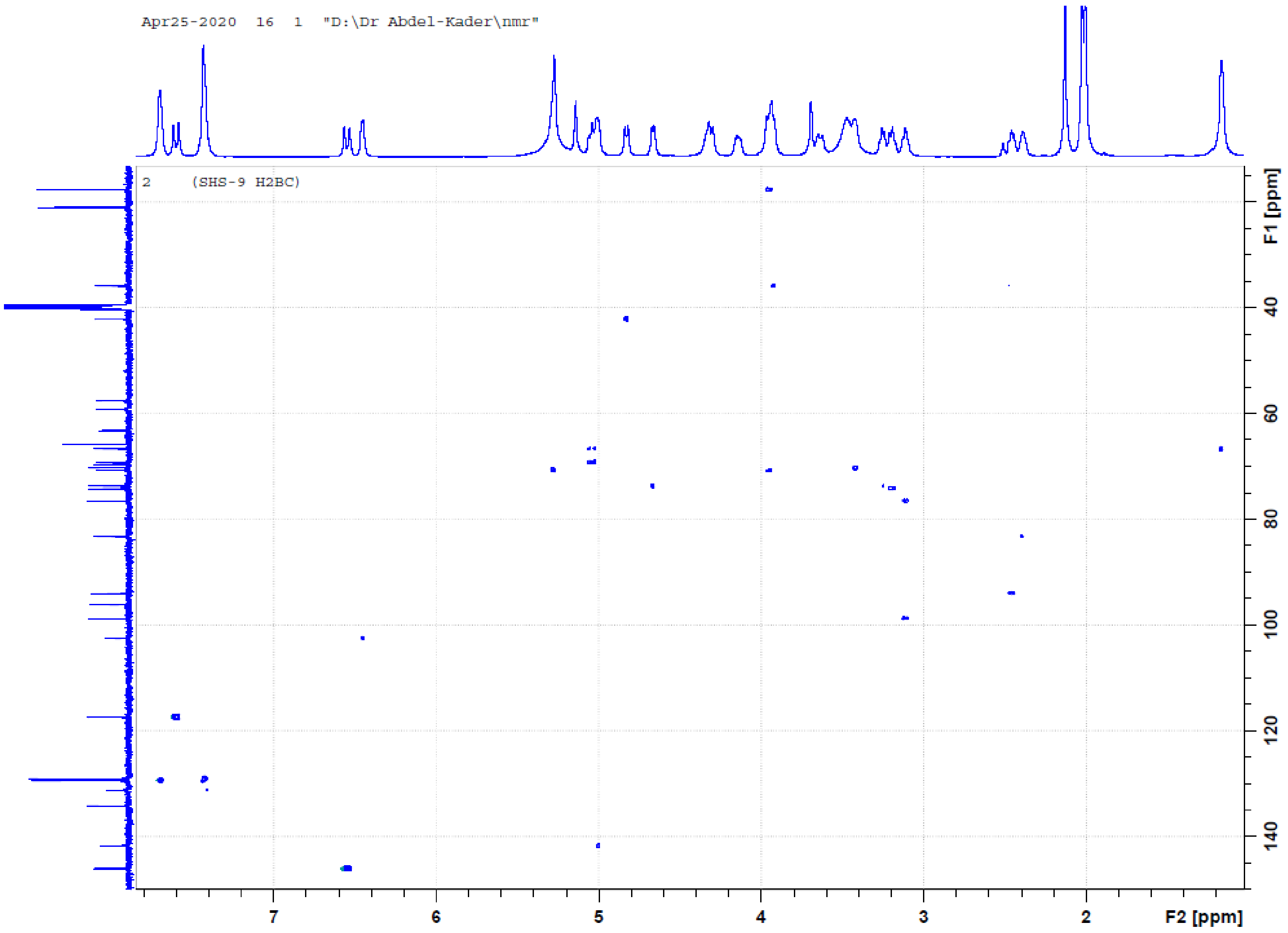


**Figure S31.** H2BC spectrum of compound 2.

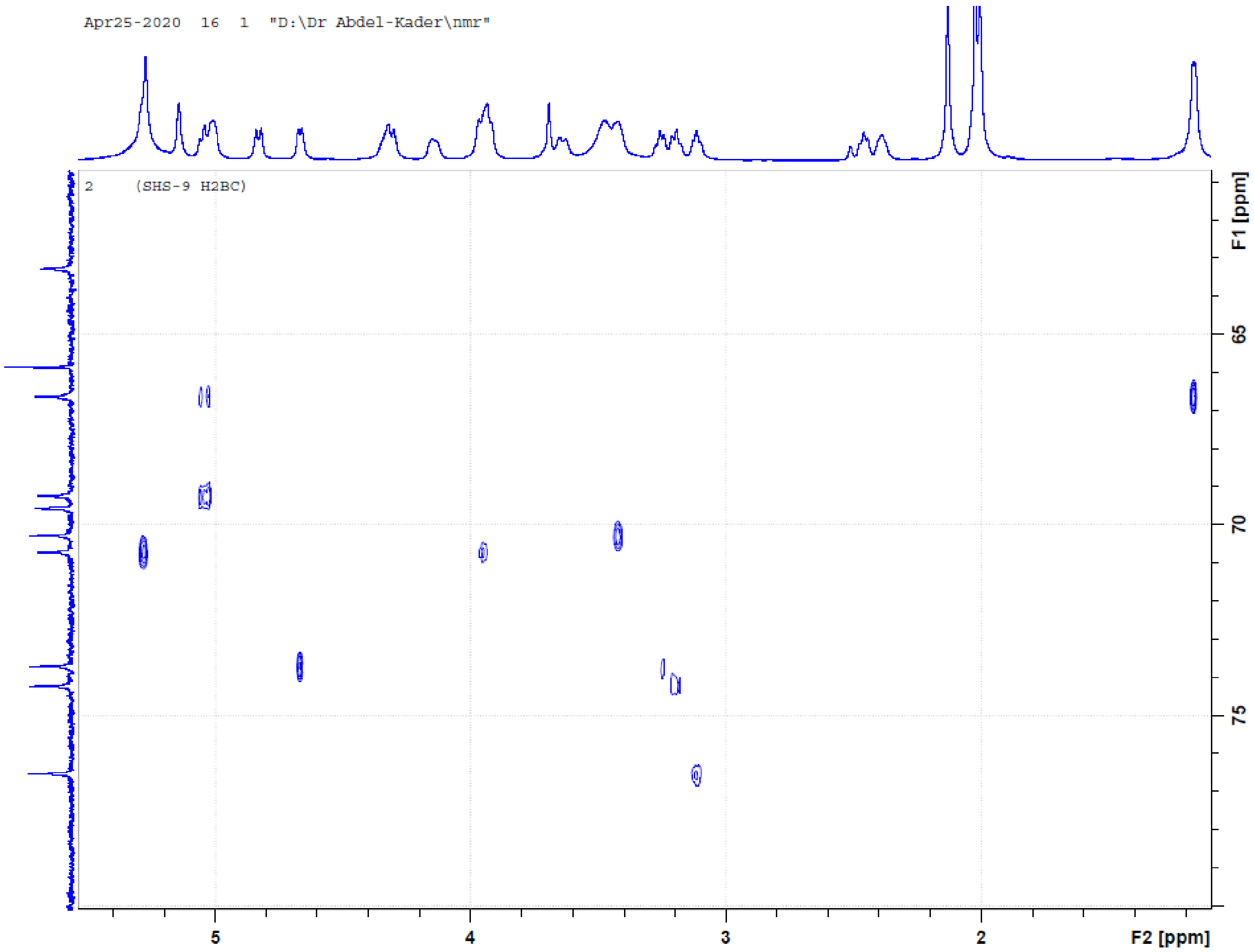


**Figure S32.** H2BC spectrum of compound 2 (expansion).

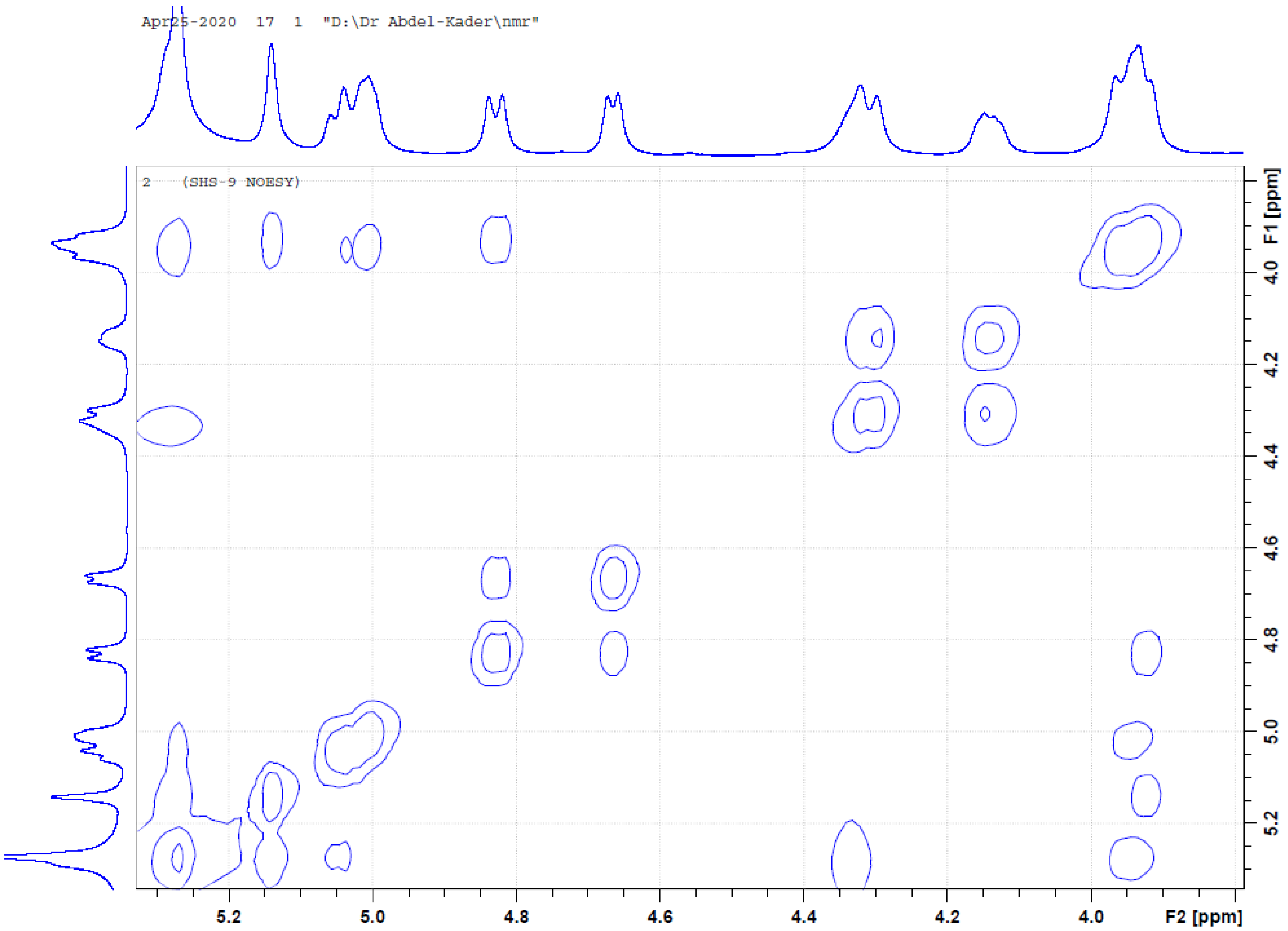


**Figure S33.** NOESY spectrum of compound 2 (Expansion).

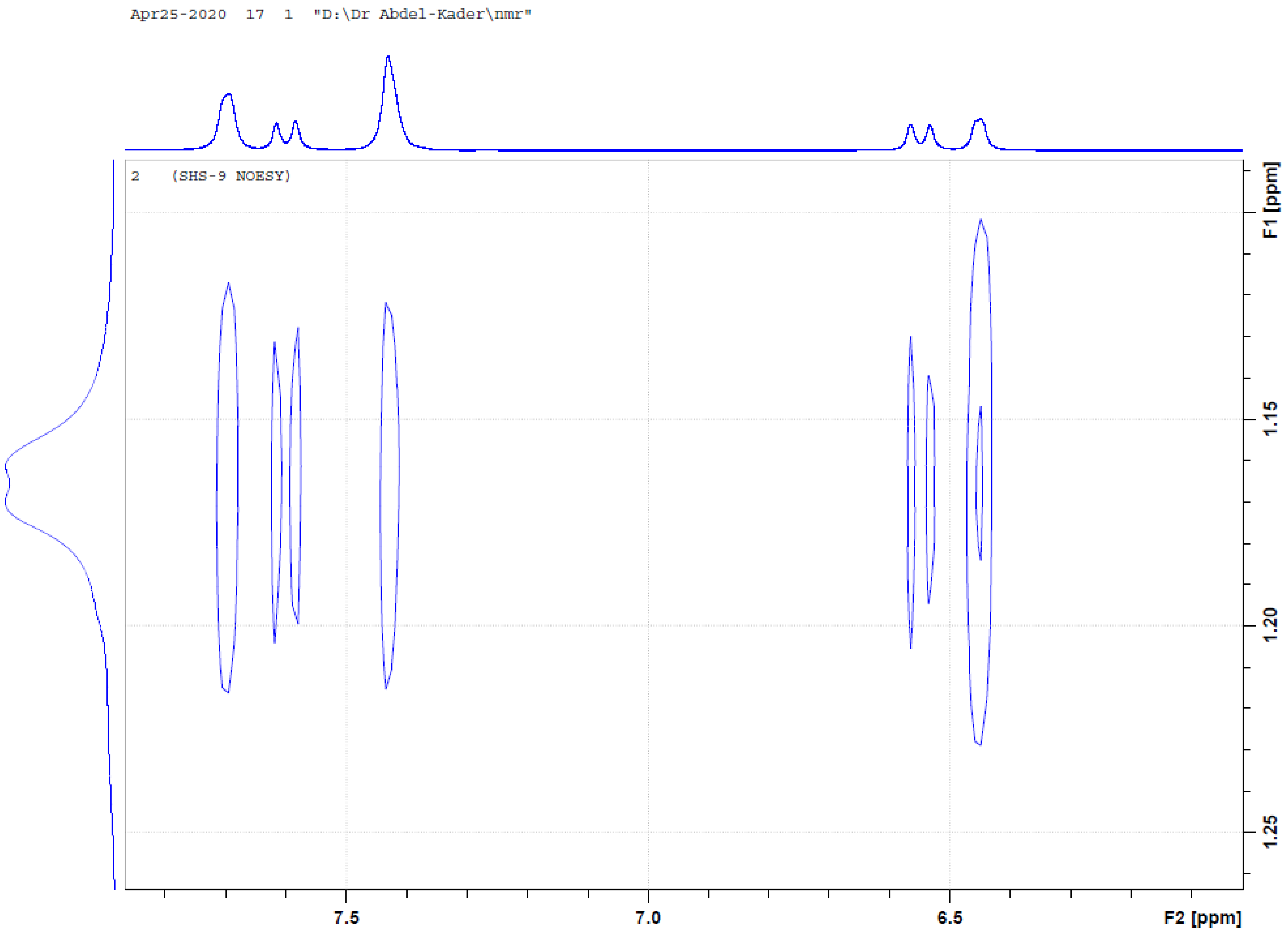


**Figure S34.** NOESY spectrum of compound 2 (Expansion).

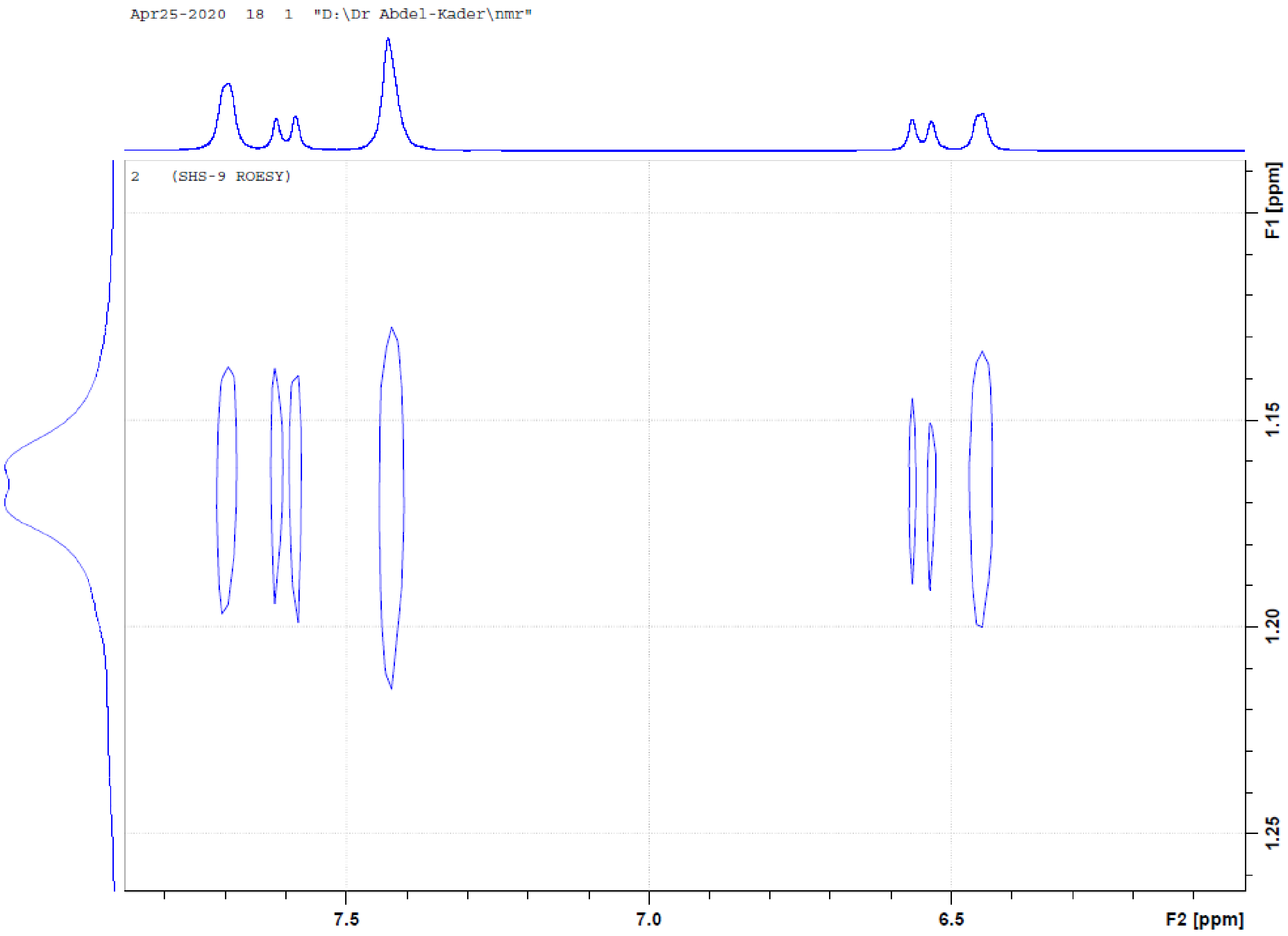


**Figure S35.** ROESY spectrum of compound 2 (Expansion).

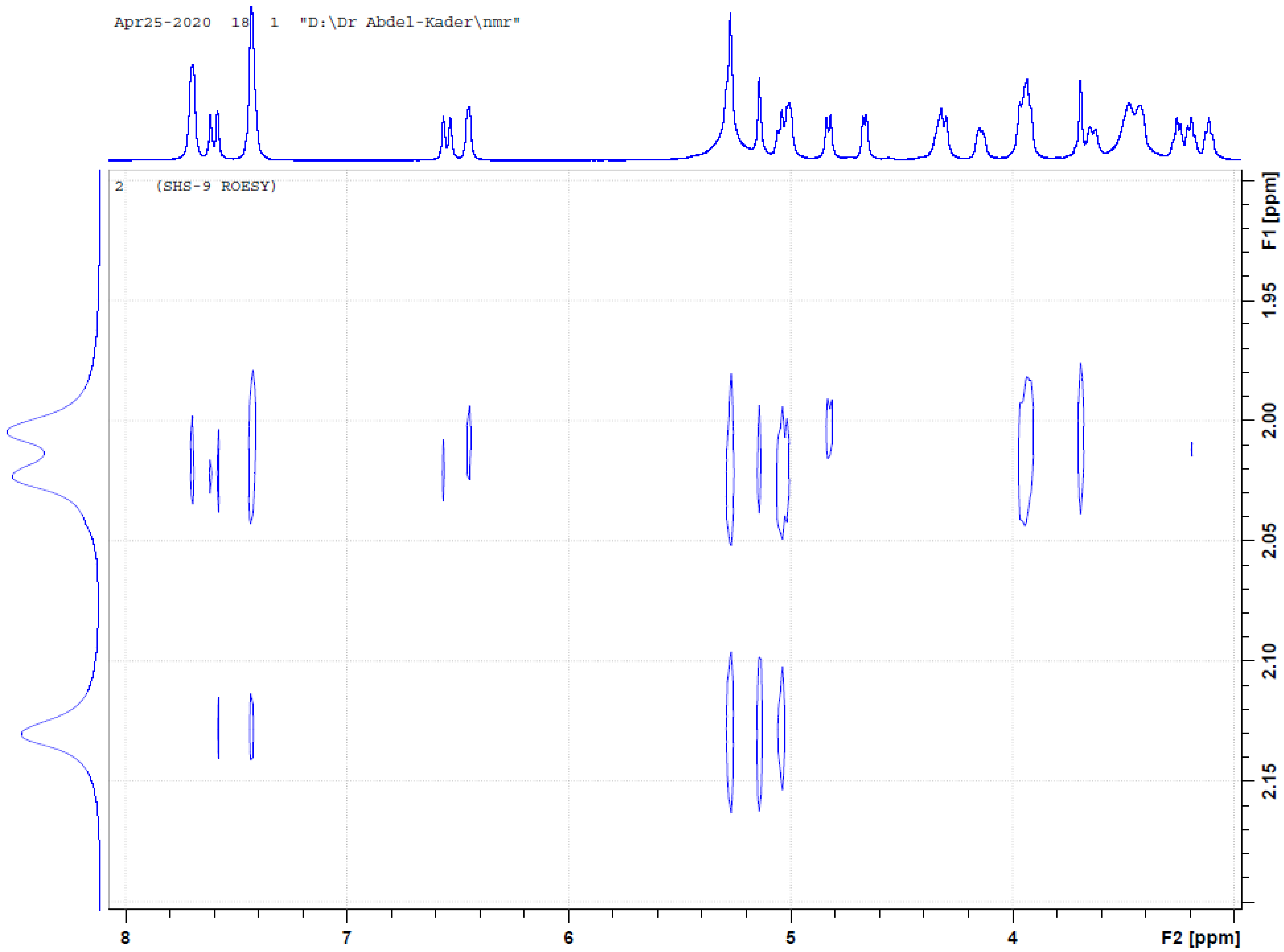


**Figure S36.** ROESY spectrum of compound 2 (Expansion).

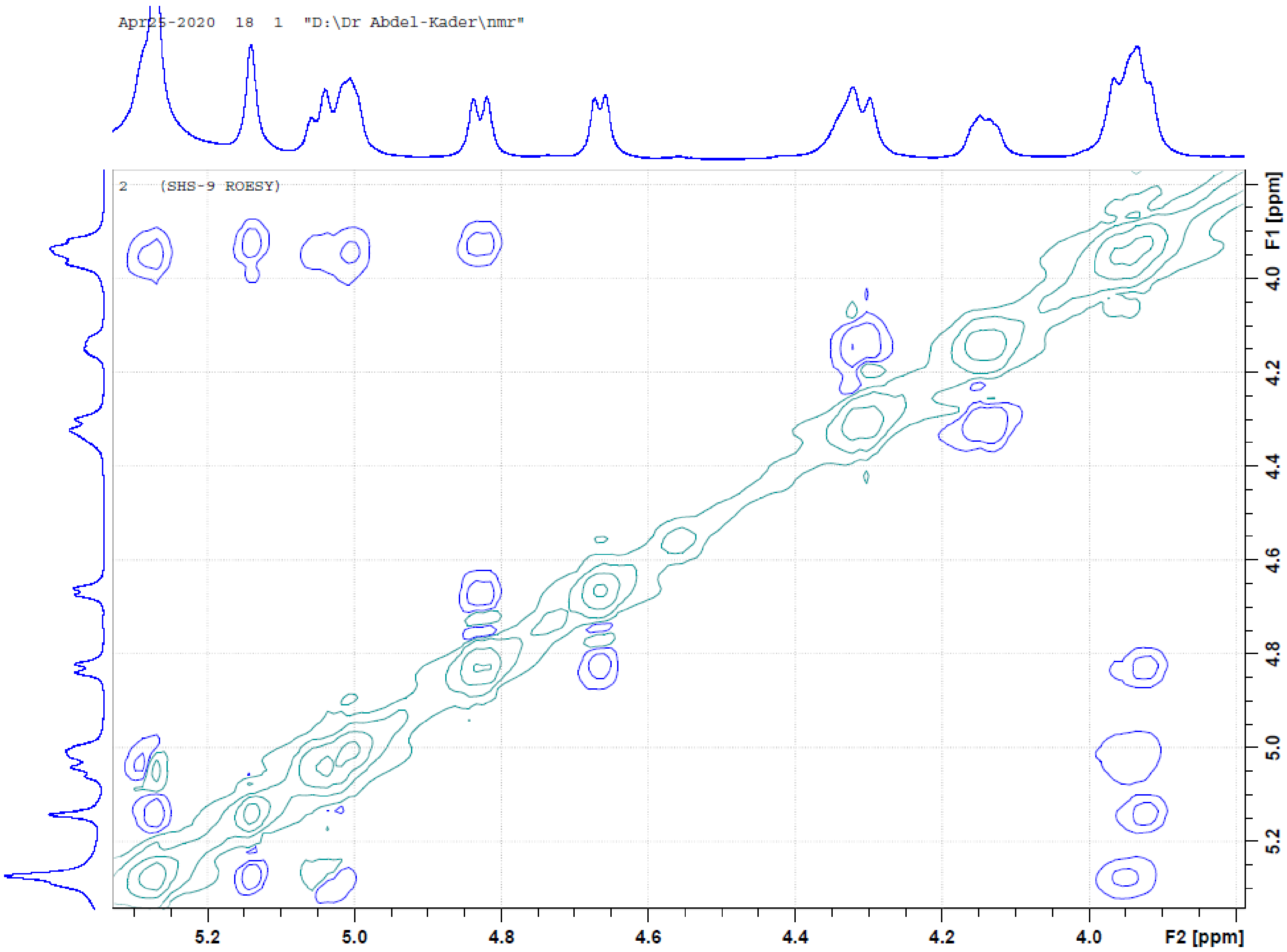


**Figure S37.** ROESY spectrum of compound 2 (Expansion).

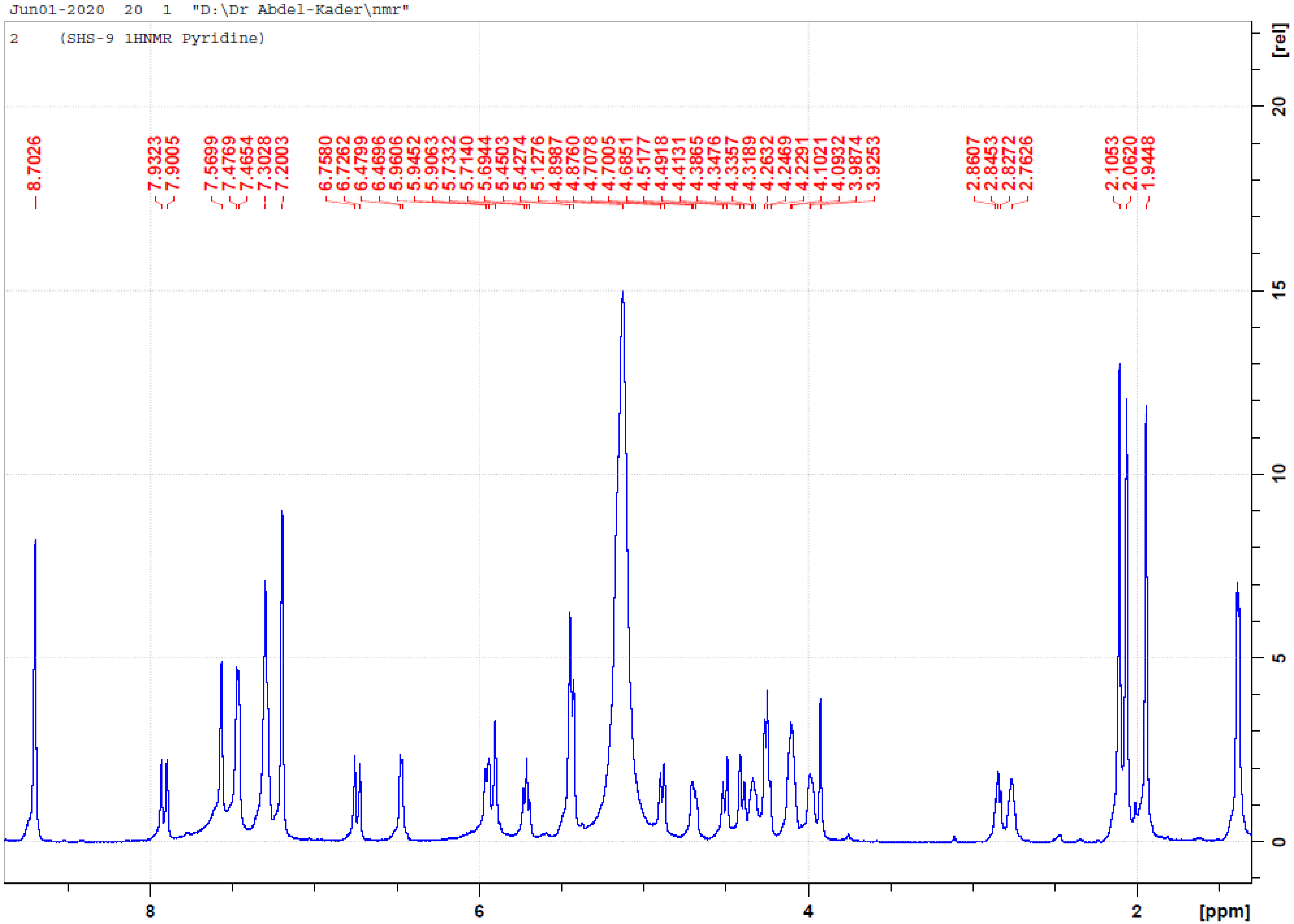


**Figure S38.** $^{1}$HNMR spectrum of compound 2 in Pyridine.

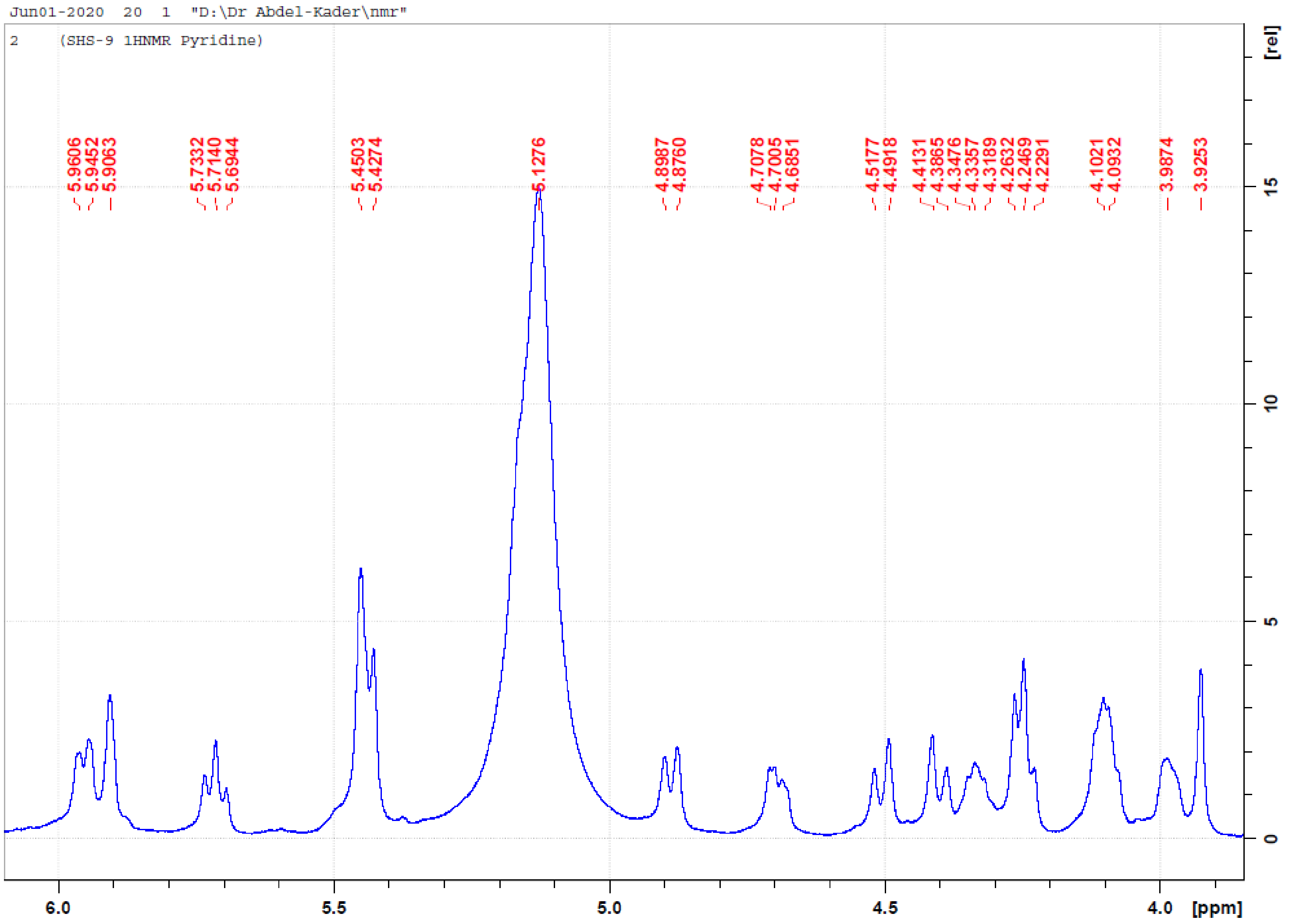


**Figure S39.** $^{1}$HNMR spectrum of compound 2 in Pyridine (expansion).

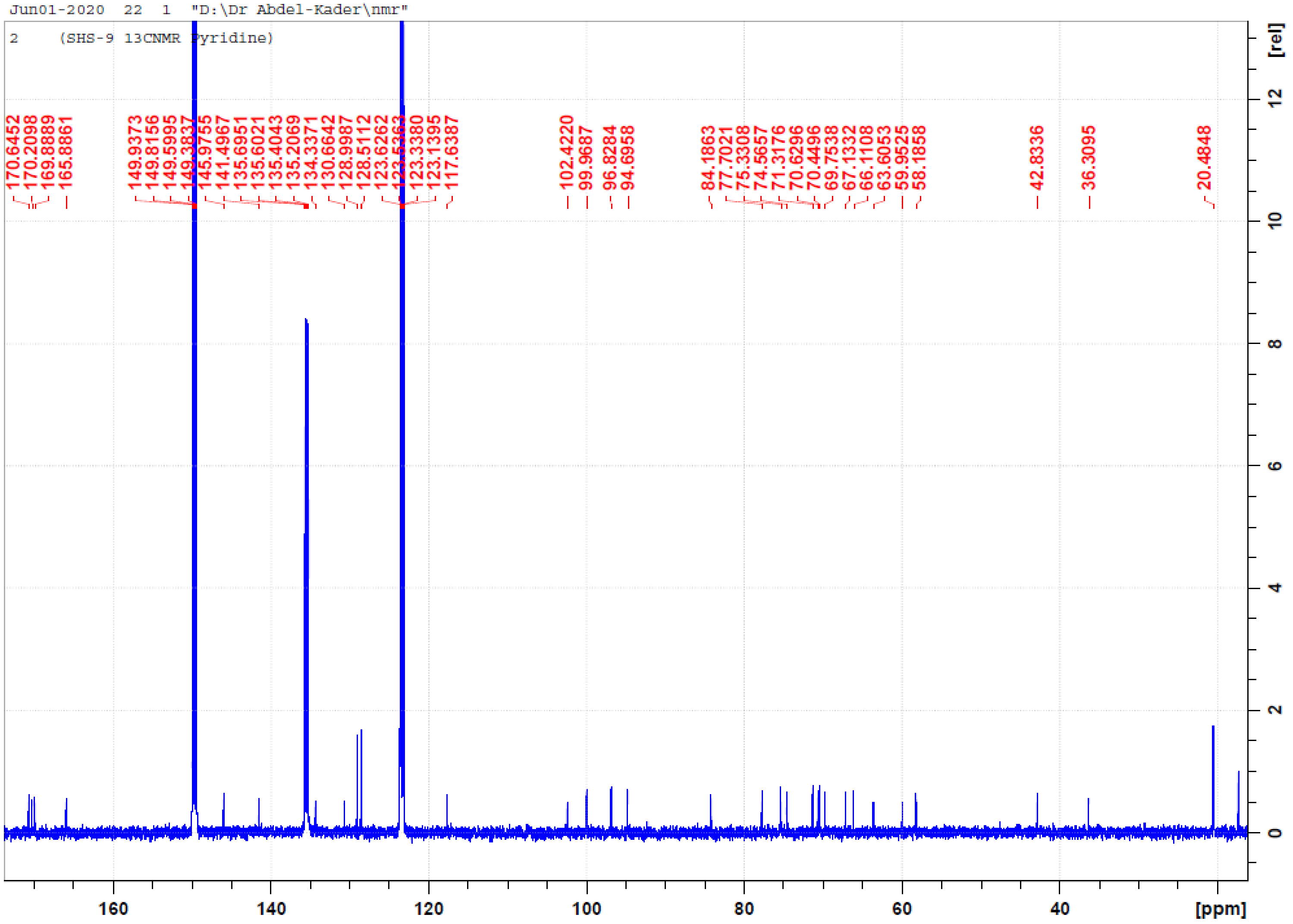


**Figure S40.** $^{13}$CNMR spectrum of compound 2 in Pyridine.

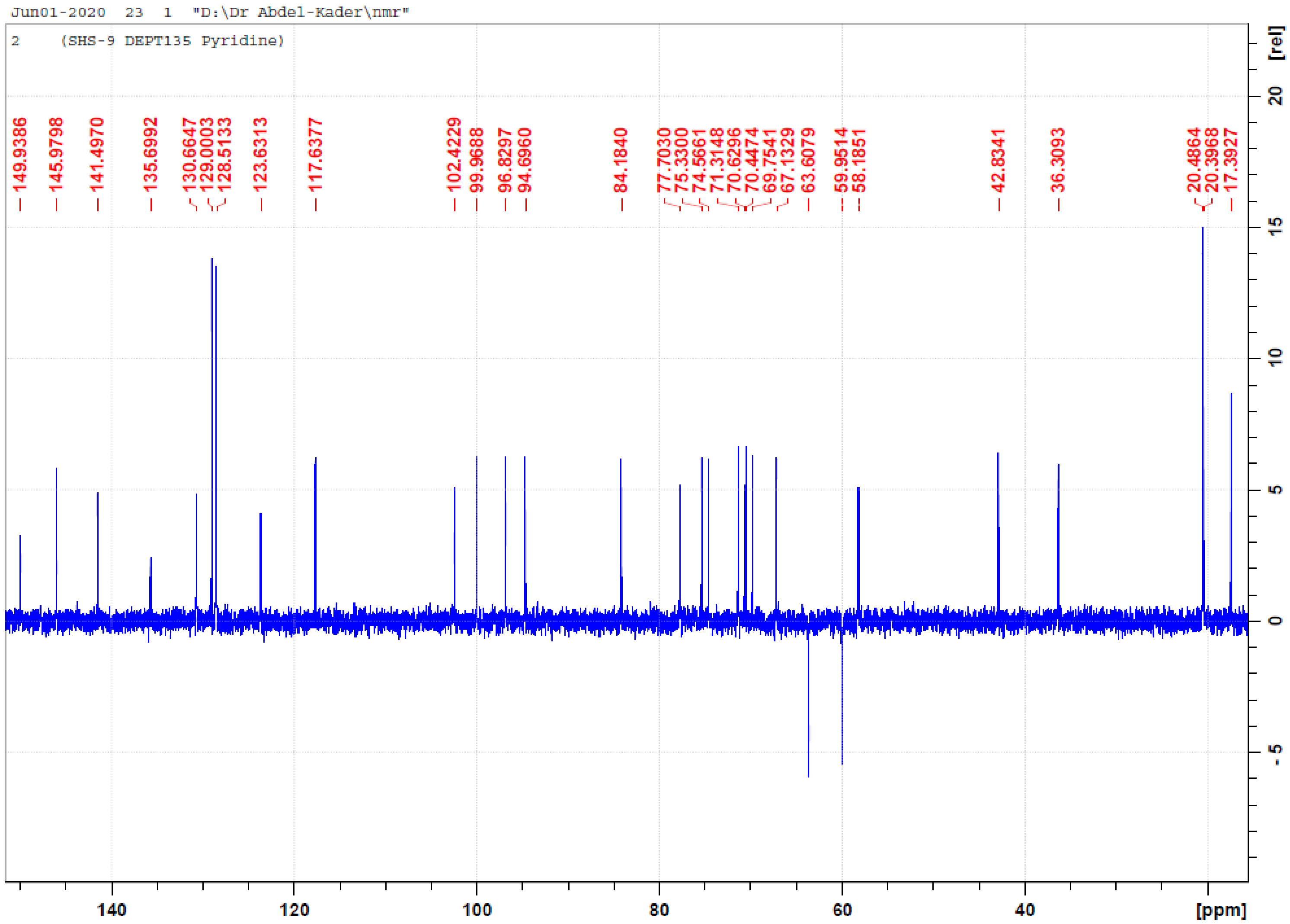


**Figure S41.** DEPT135 spectrum of compound 2 in Pyridine.

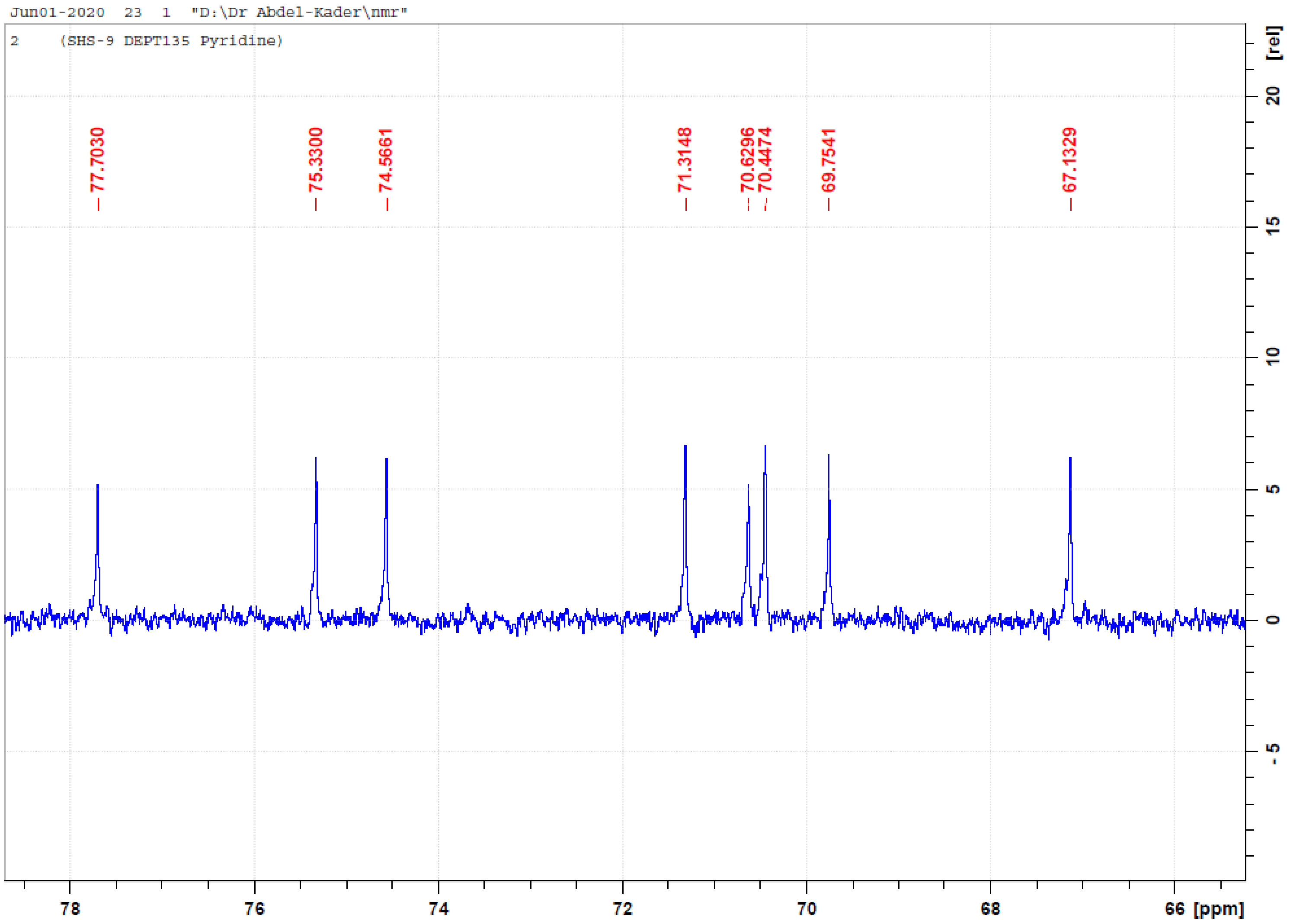


**Figure S42.** DEPT135 spectrum of compound 2 in Pyridine (expansion).

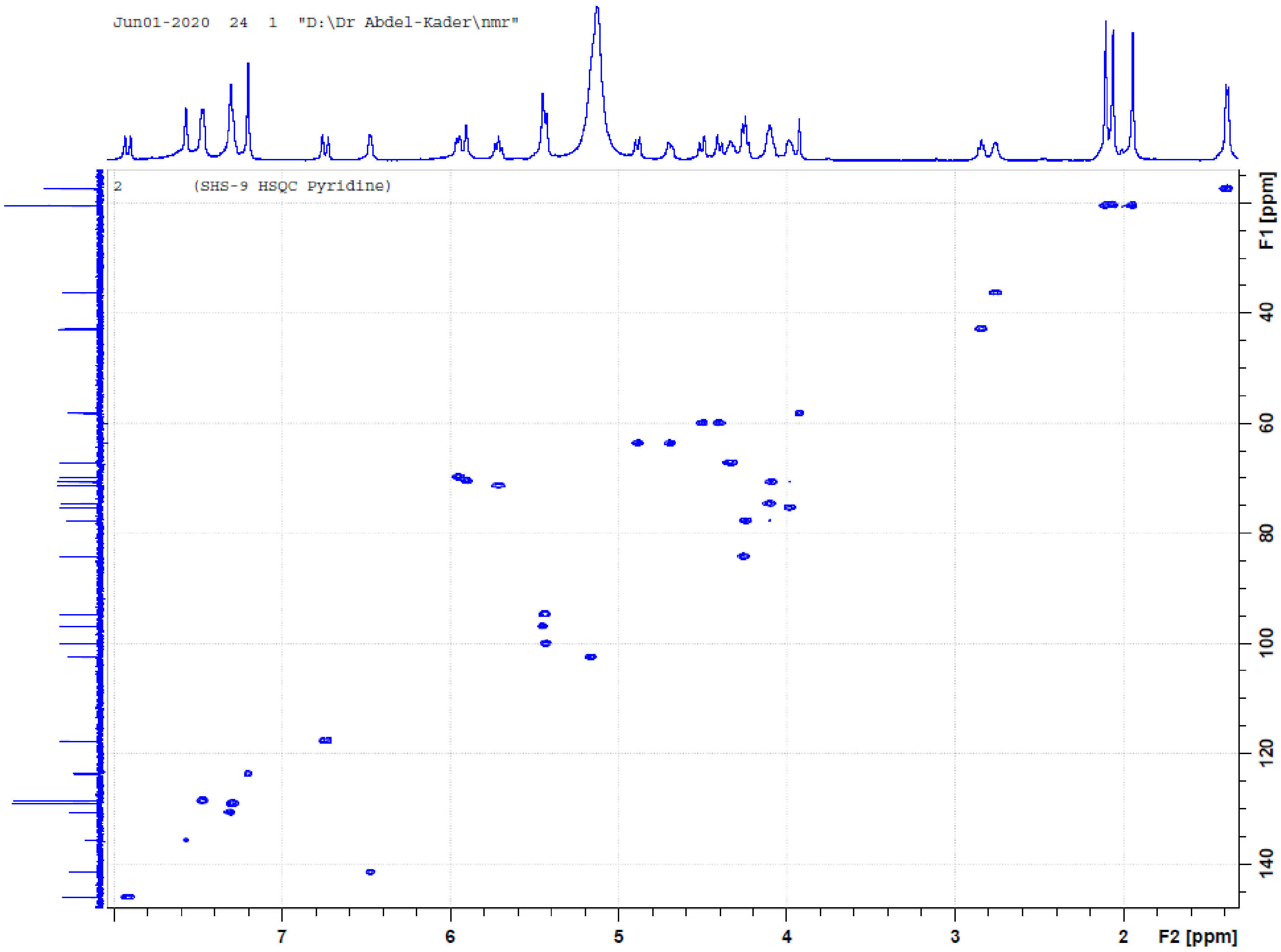


**Figure S43.** HSQC spectrum of compound 2 in Pyridine.

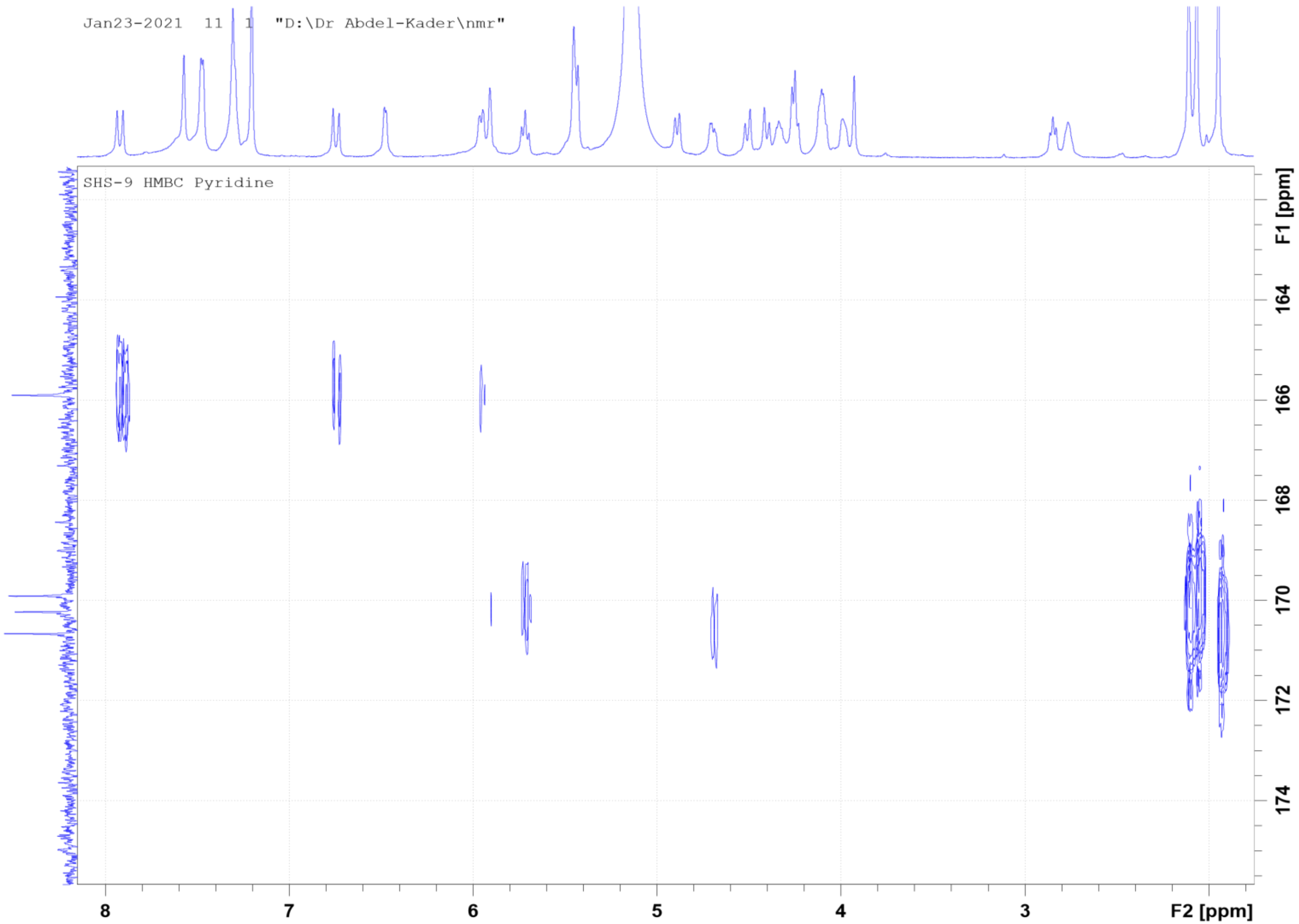


**Figure S44.** HMBC spectrum of compound 2 in Pyridine (expansion).

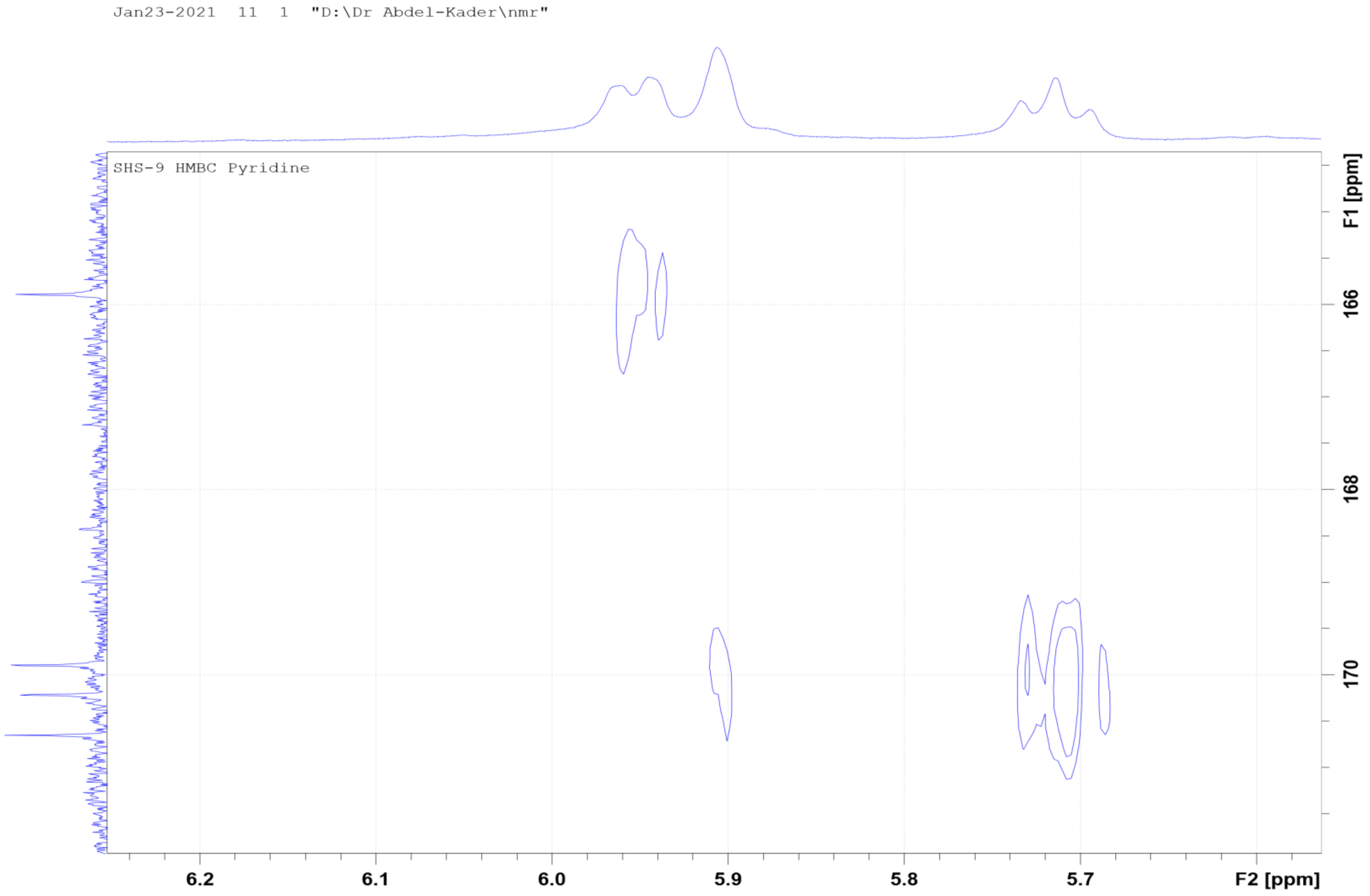


**Figure S45.** HMBC spectrum of compound 2 in Pyridine (expansion).

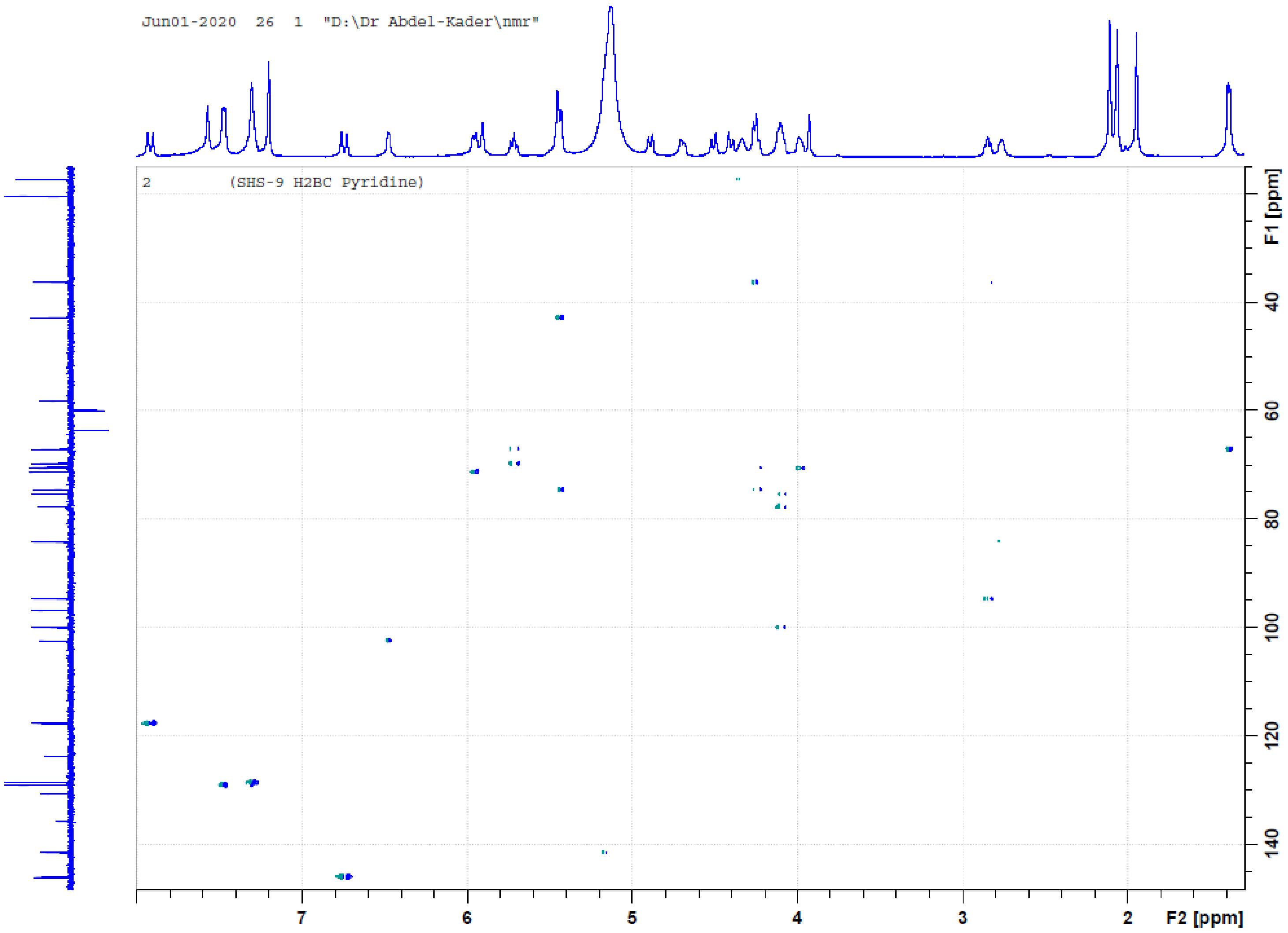


**Figure S46.** H2BC spectrum of compound 2 in Pyridine.

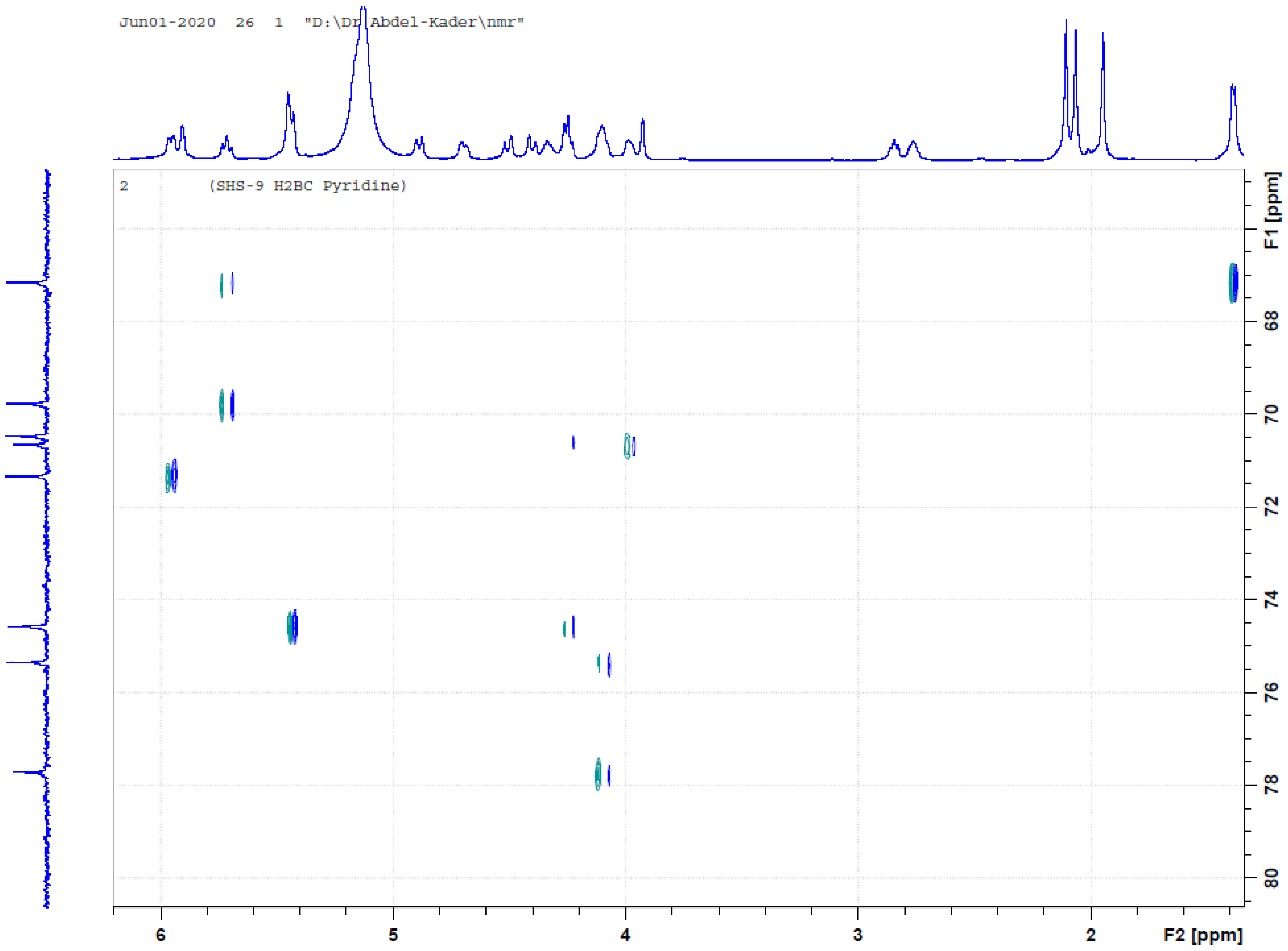


**Figure S47.** H2BC spectrum of compound 2 in Pyridine (expansion).

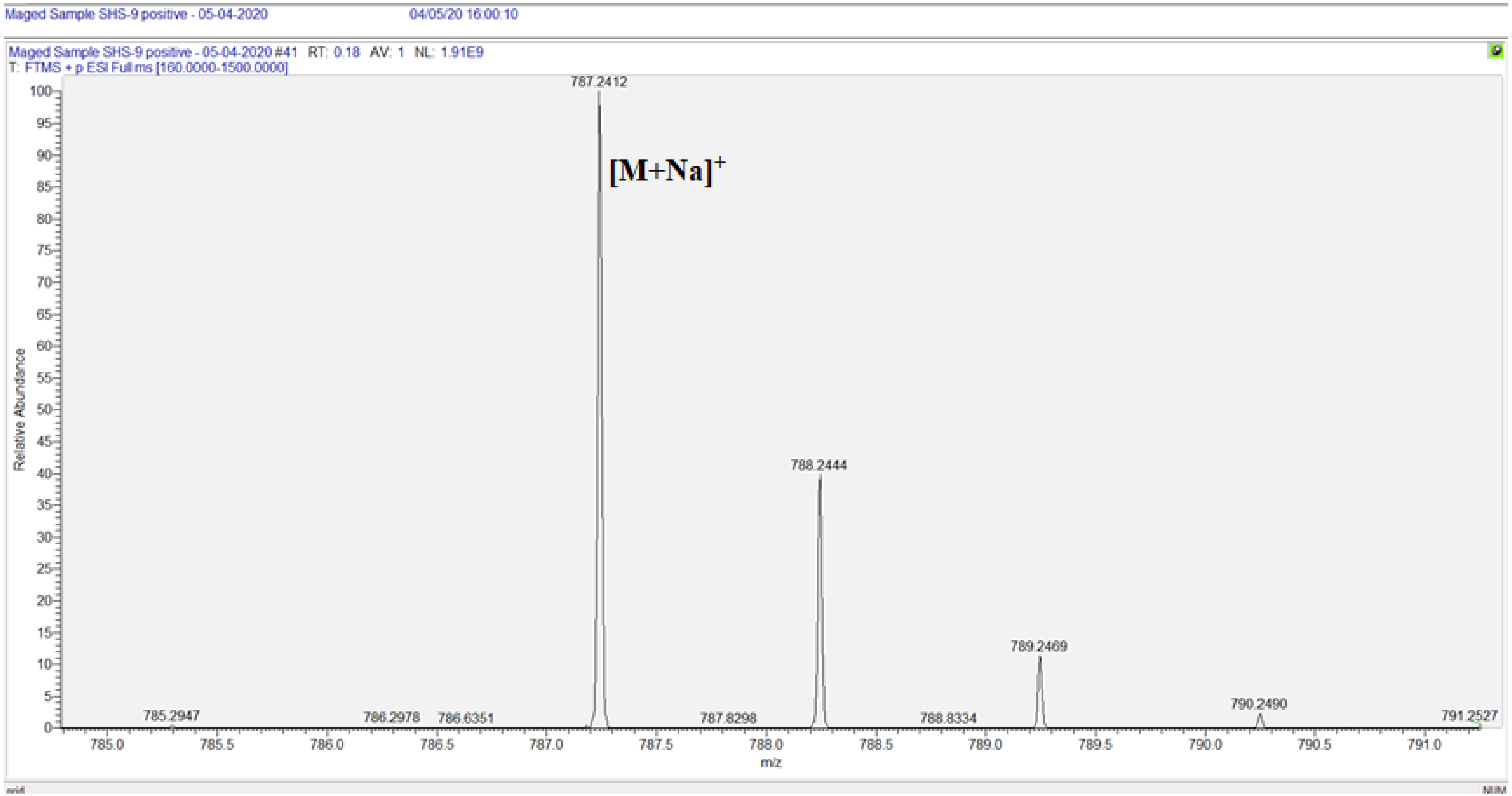


**Figure S48.** HRESIMS of compound 2 (positive mode).

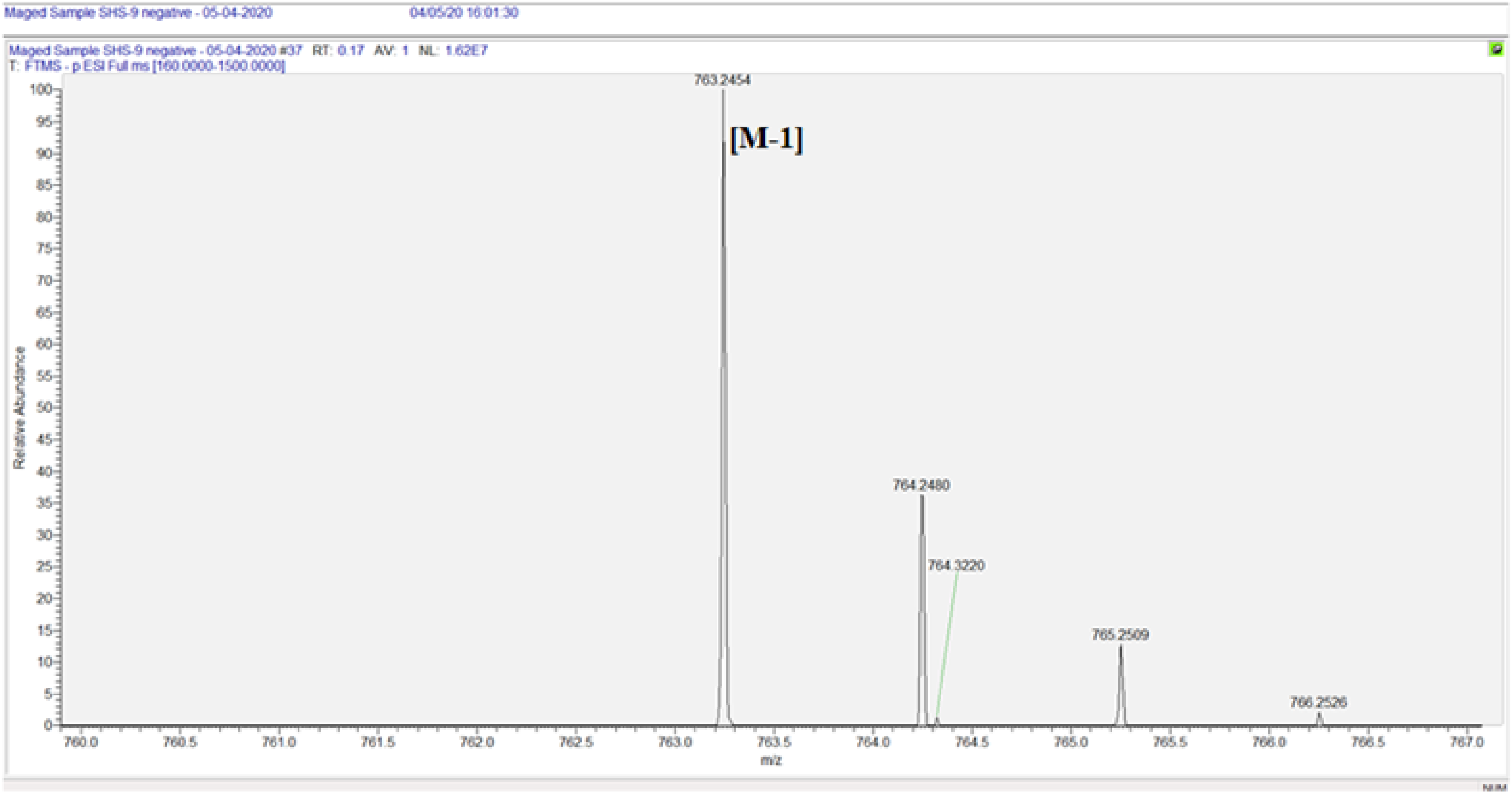


**Figure S49.** HRESIMS of compound 2 (negative mode).

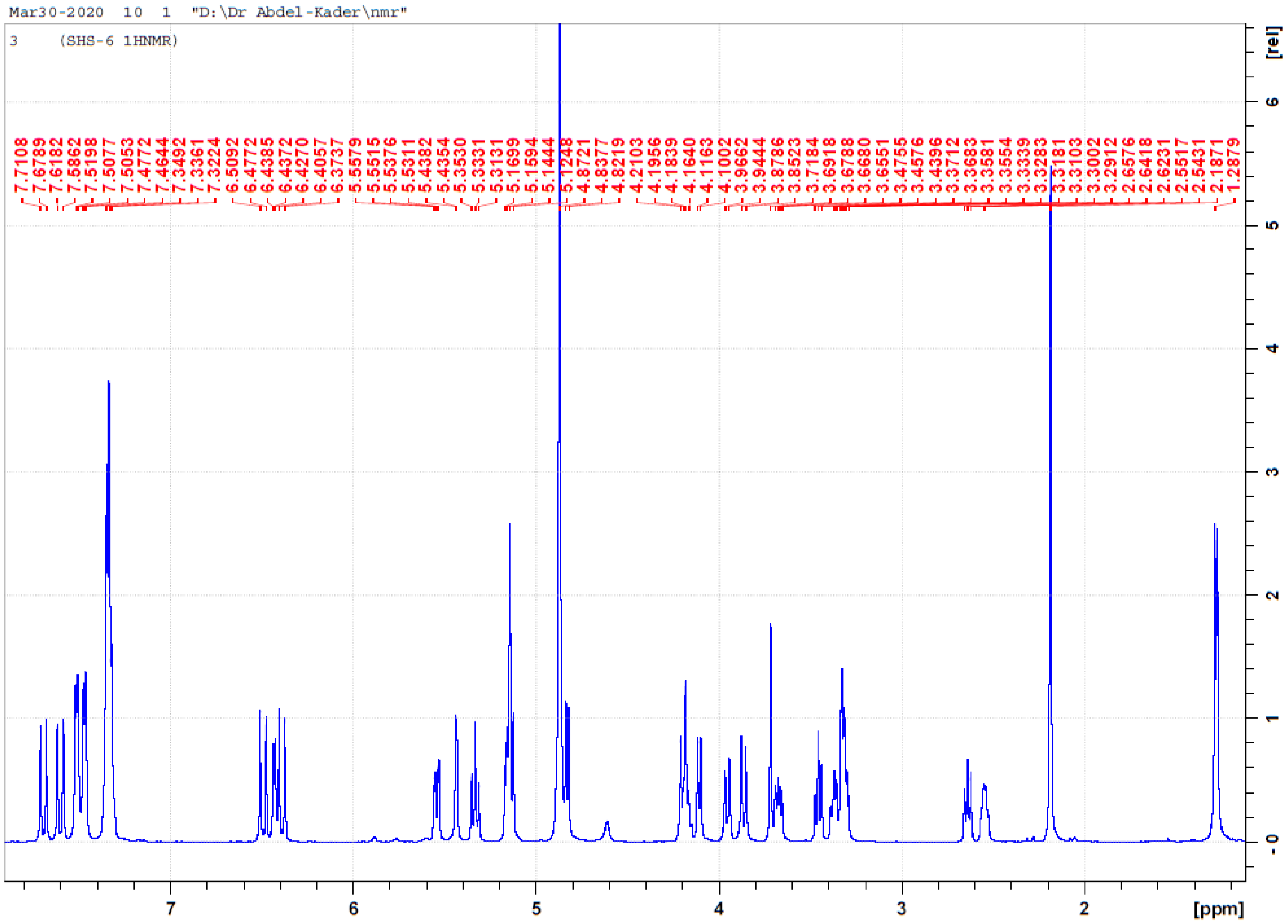


**Figure S50.** $^{1}$HNMR spectrum of compound 3.

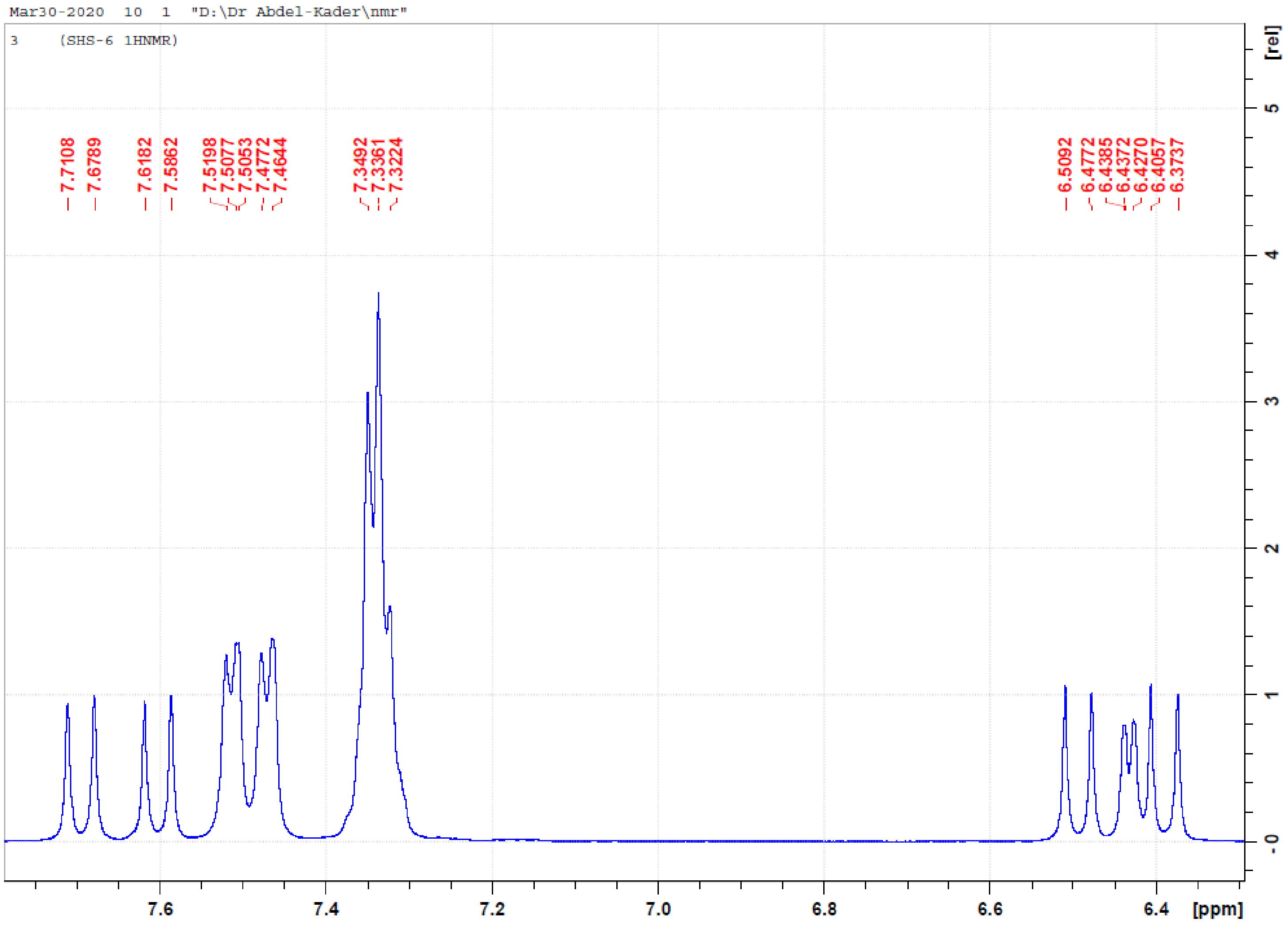


**Figure S51.** $^{1}$HNMR spectrum of compound 3 (expansion).

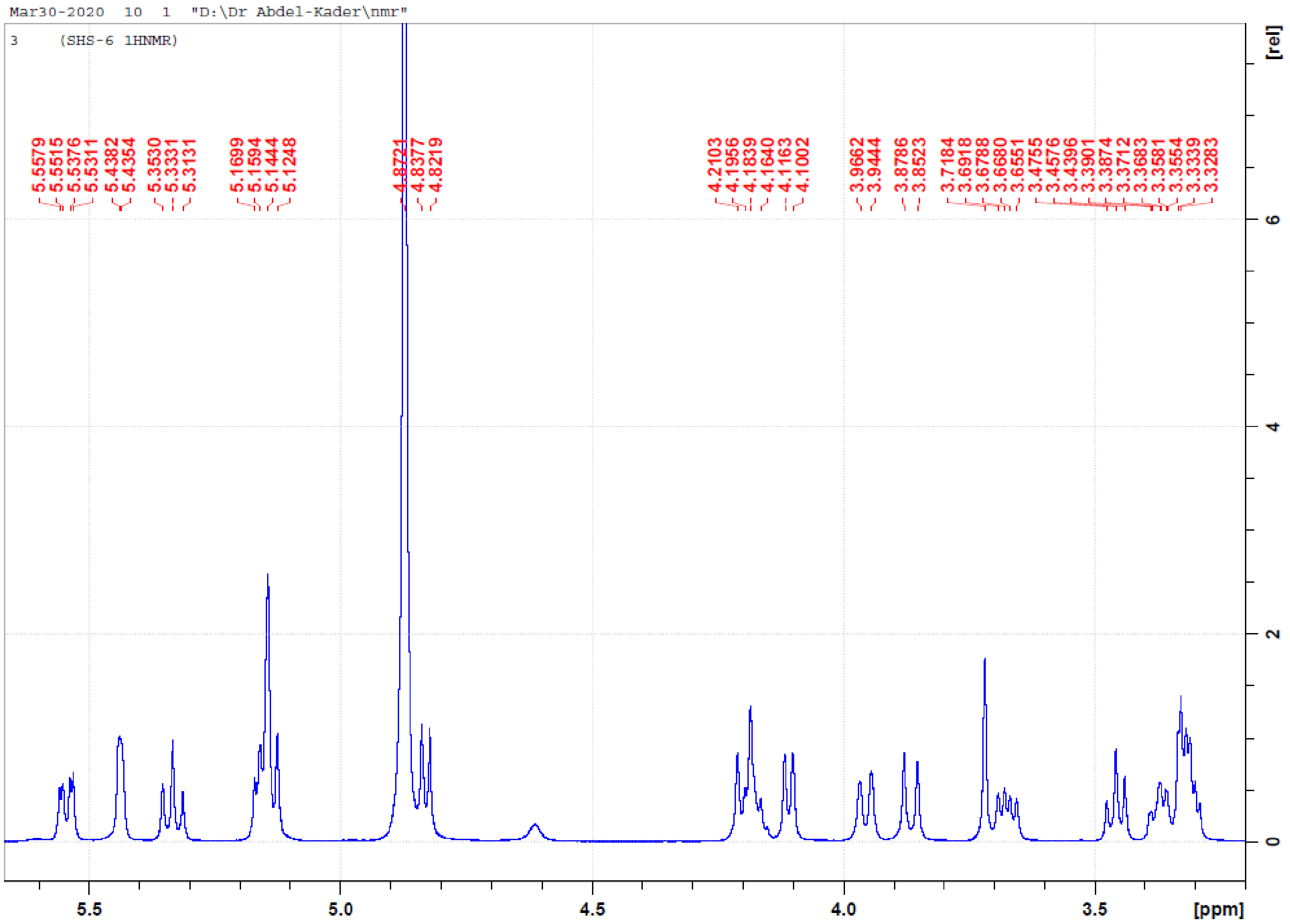


**Figure S52.** $^{1}$HNMR spectrum of compound 3 (expansion).

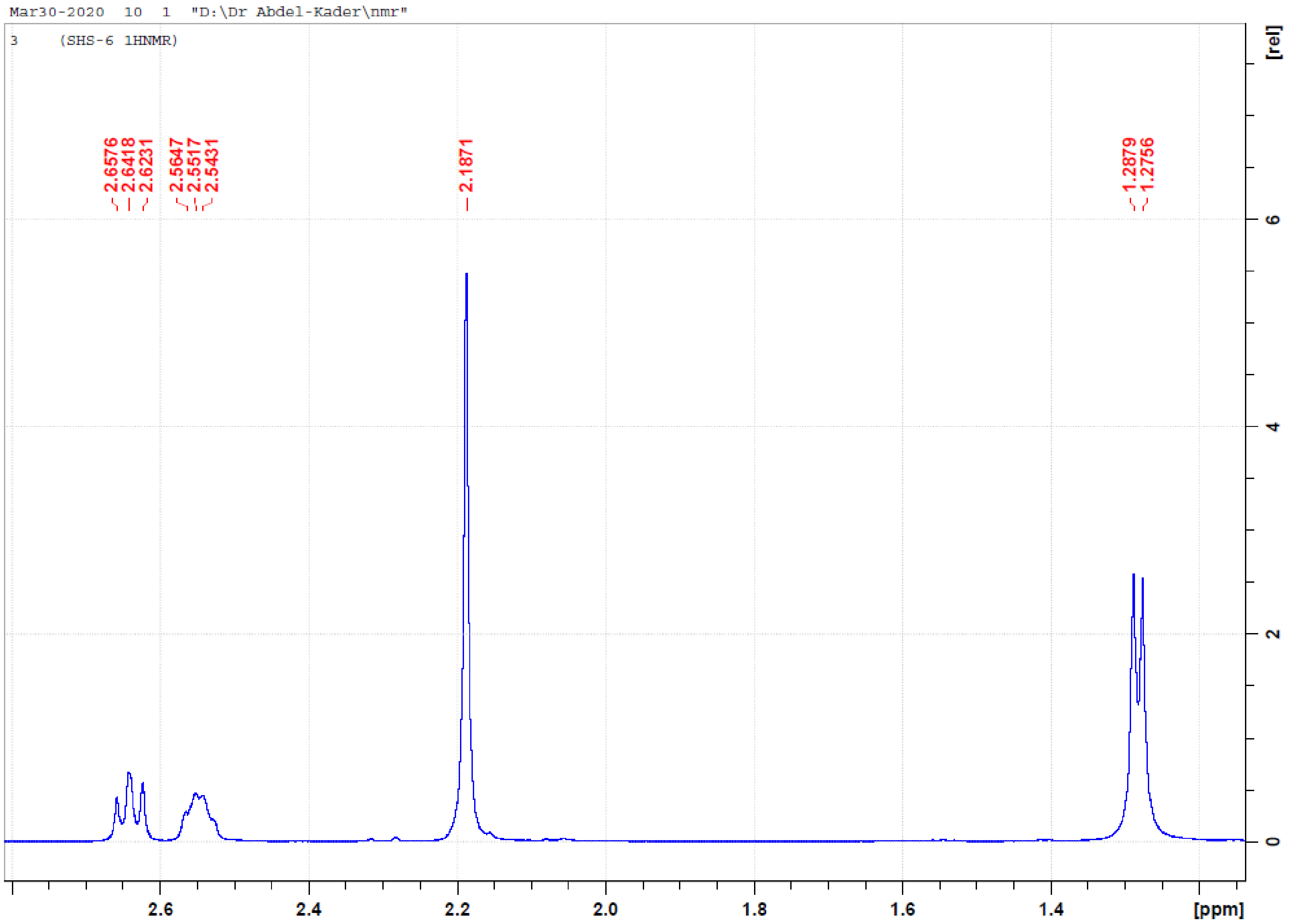


**Figure S53.** $^{1}$HNMR spectrum of compound 3 (expansion).

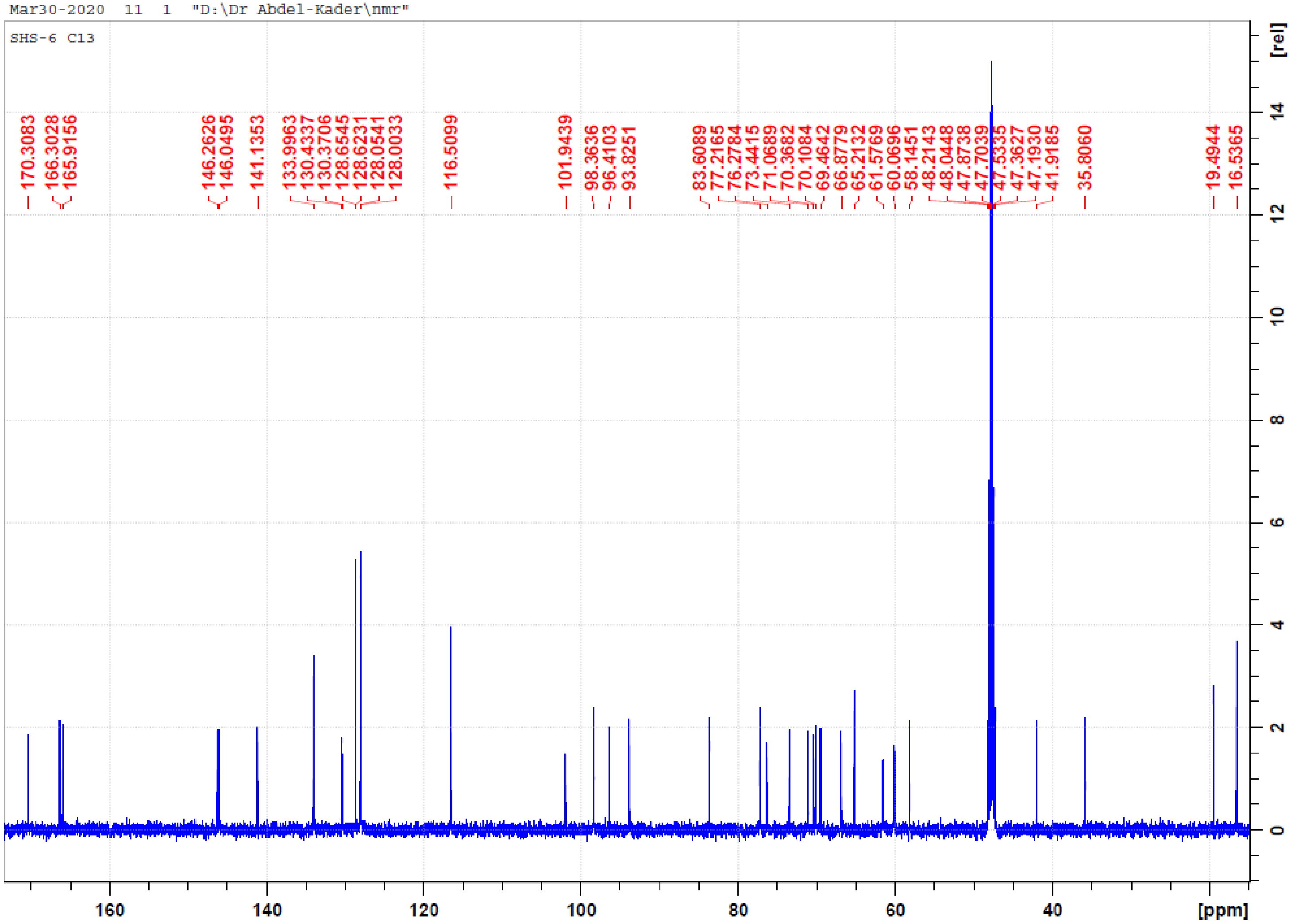


**Figure S54.** $^{13}$CNMR spectrum of compound 3.

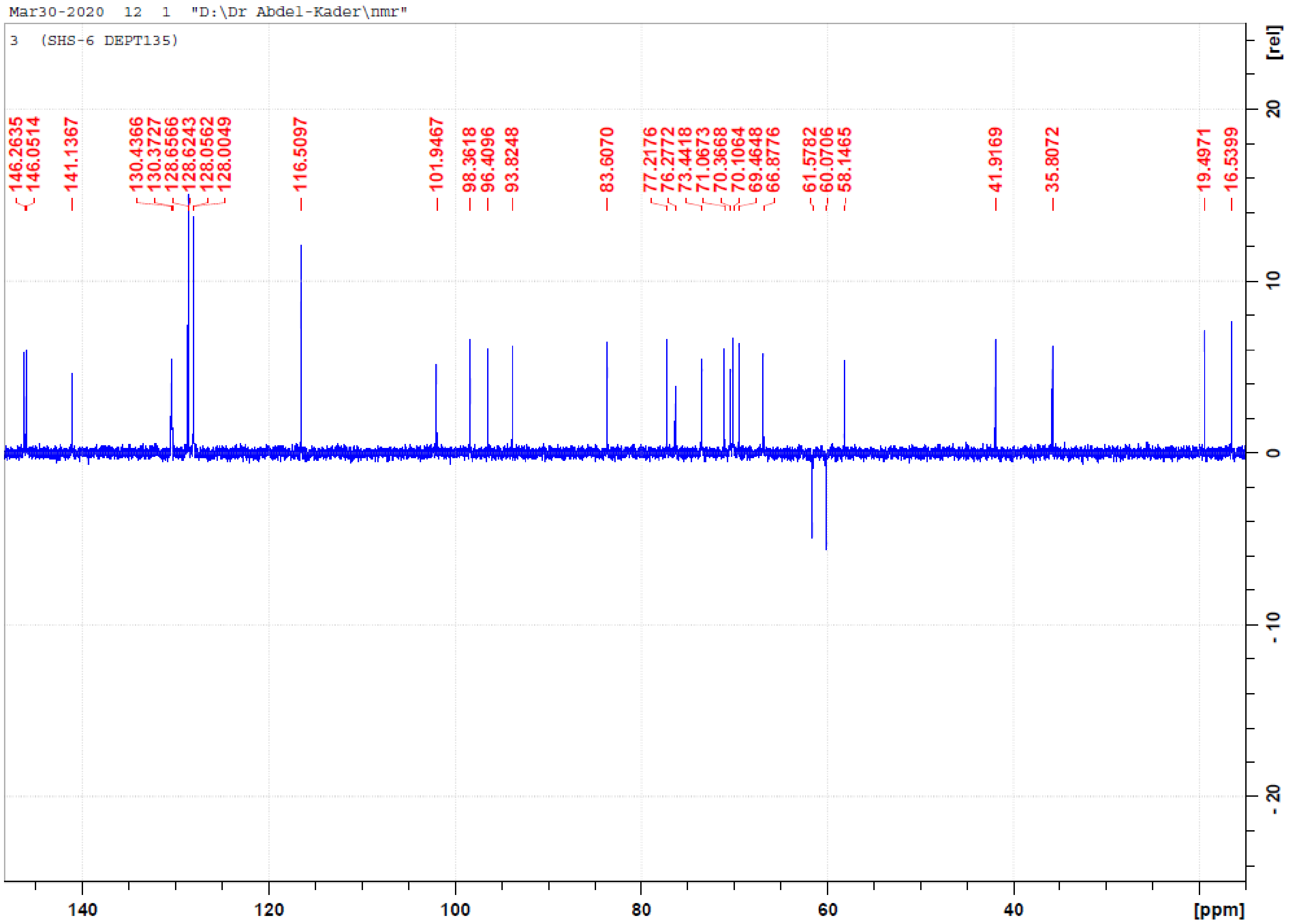


**Figure S55.** DEPT135 spectrum of compound 3.

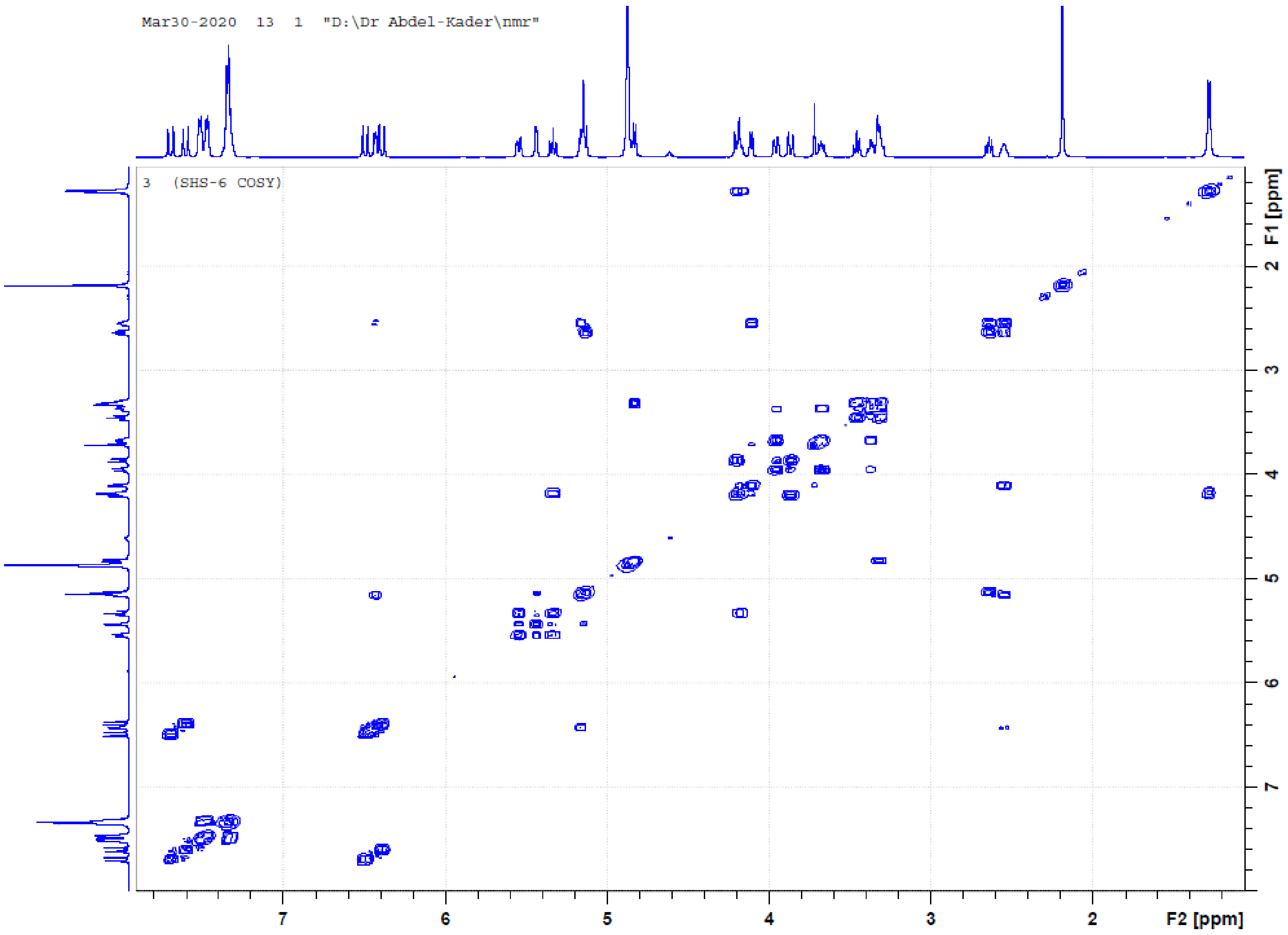


**Figure S56.** COSY spectrum of compound 3.

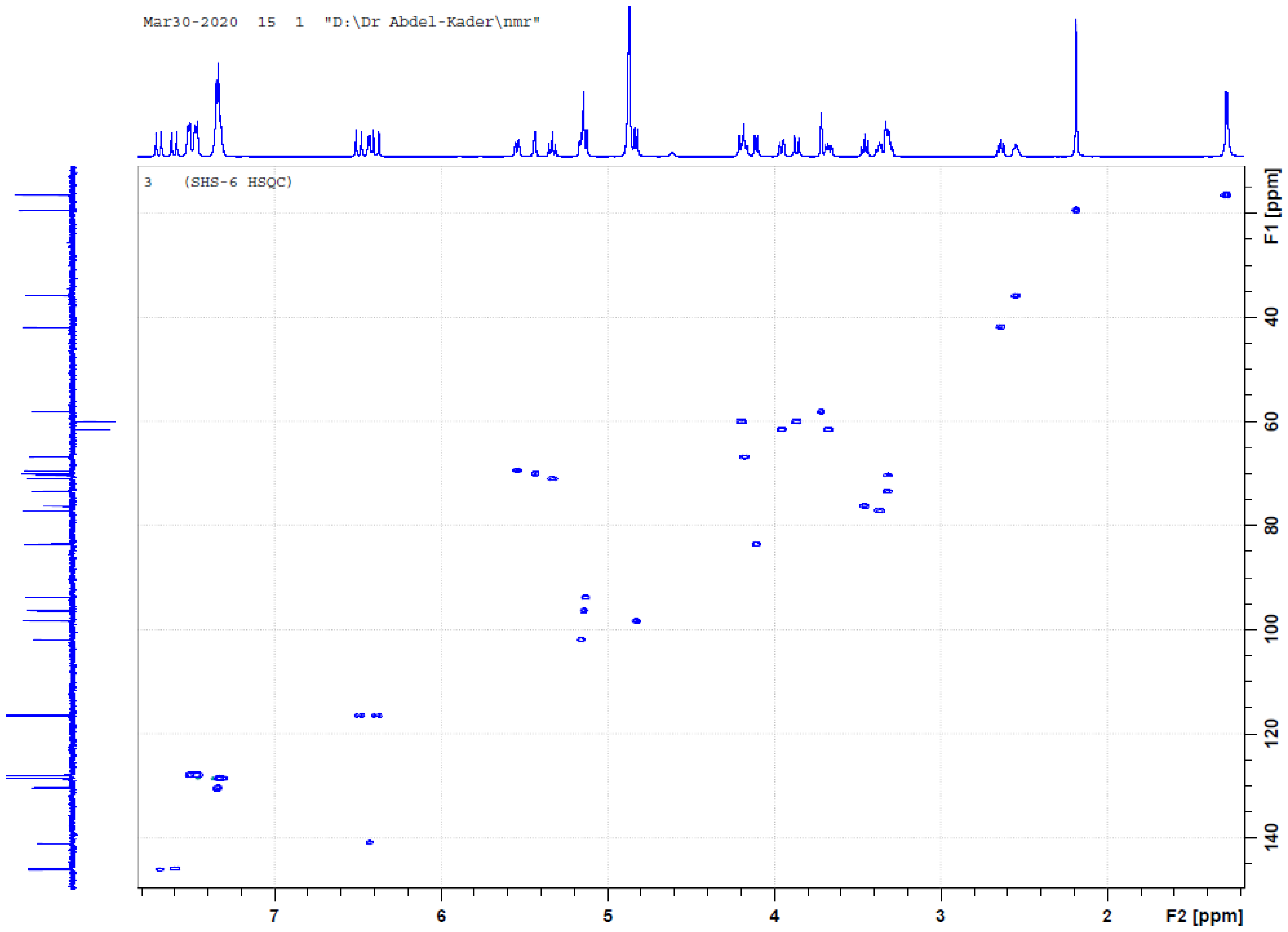


**Figure S57.** HSQC spectrum of compound 3.

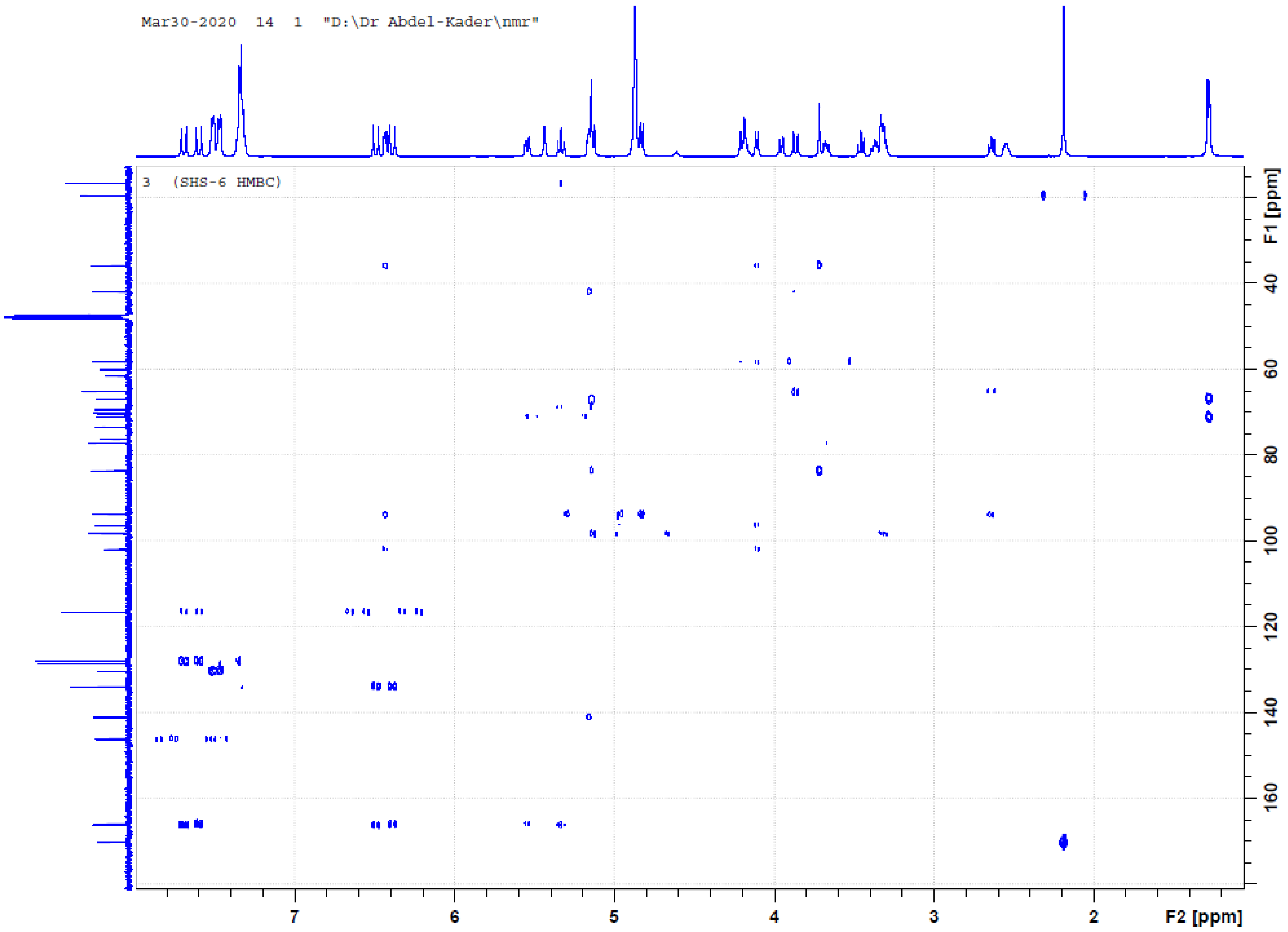


**Figure S58.** HMBC spectrum of compound 3.

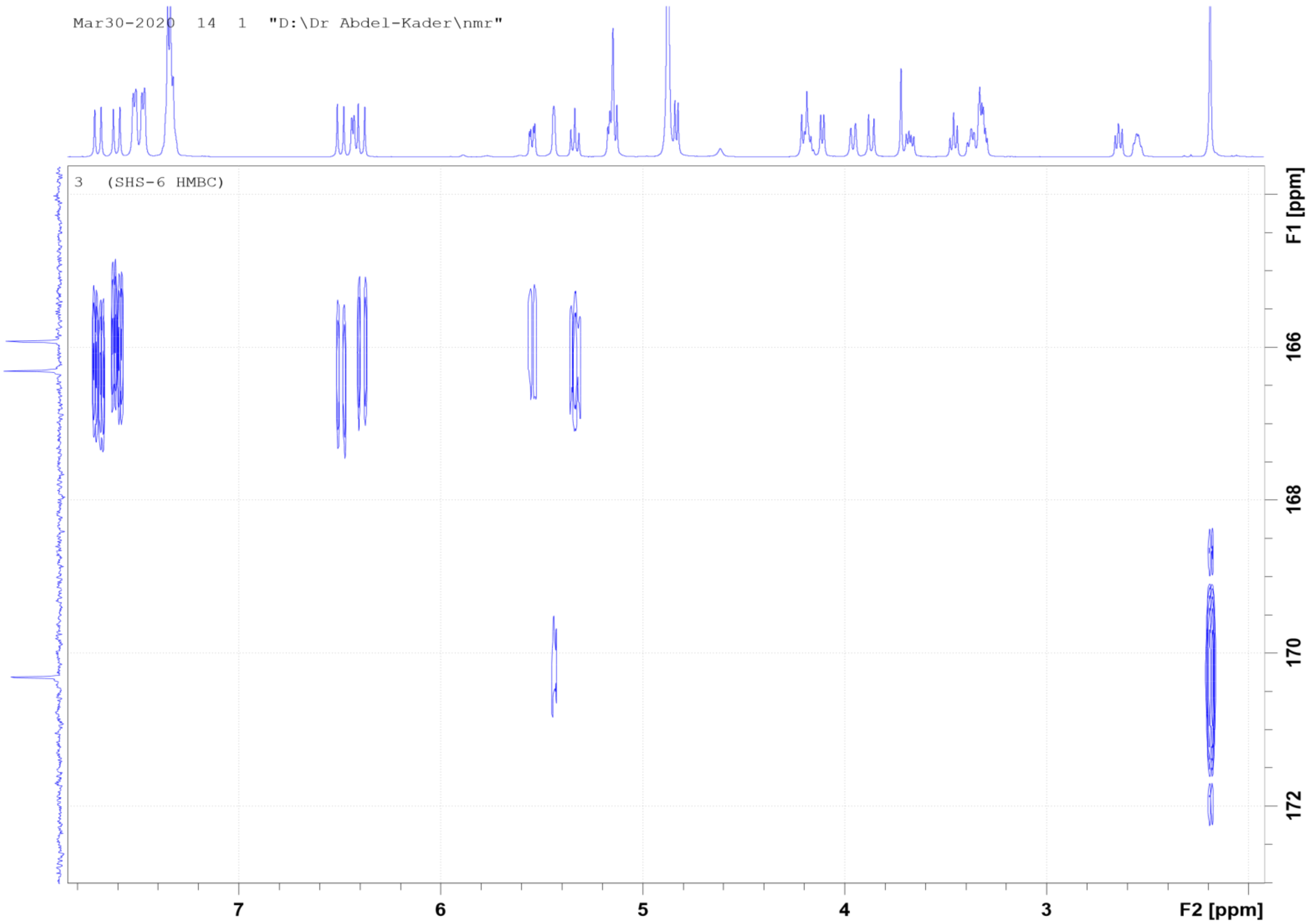


**Figure S59.** HMBC spectrum of compound 3 (expansion).

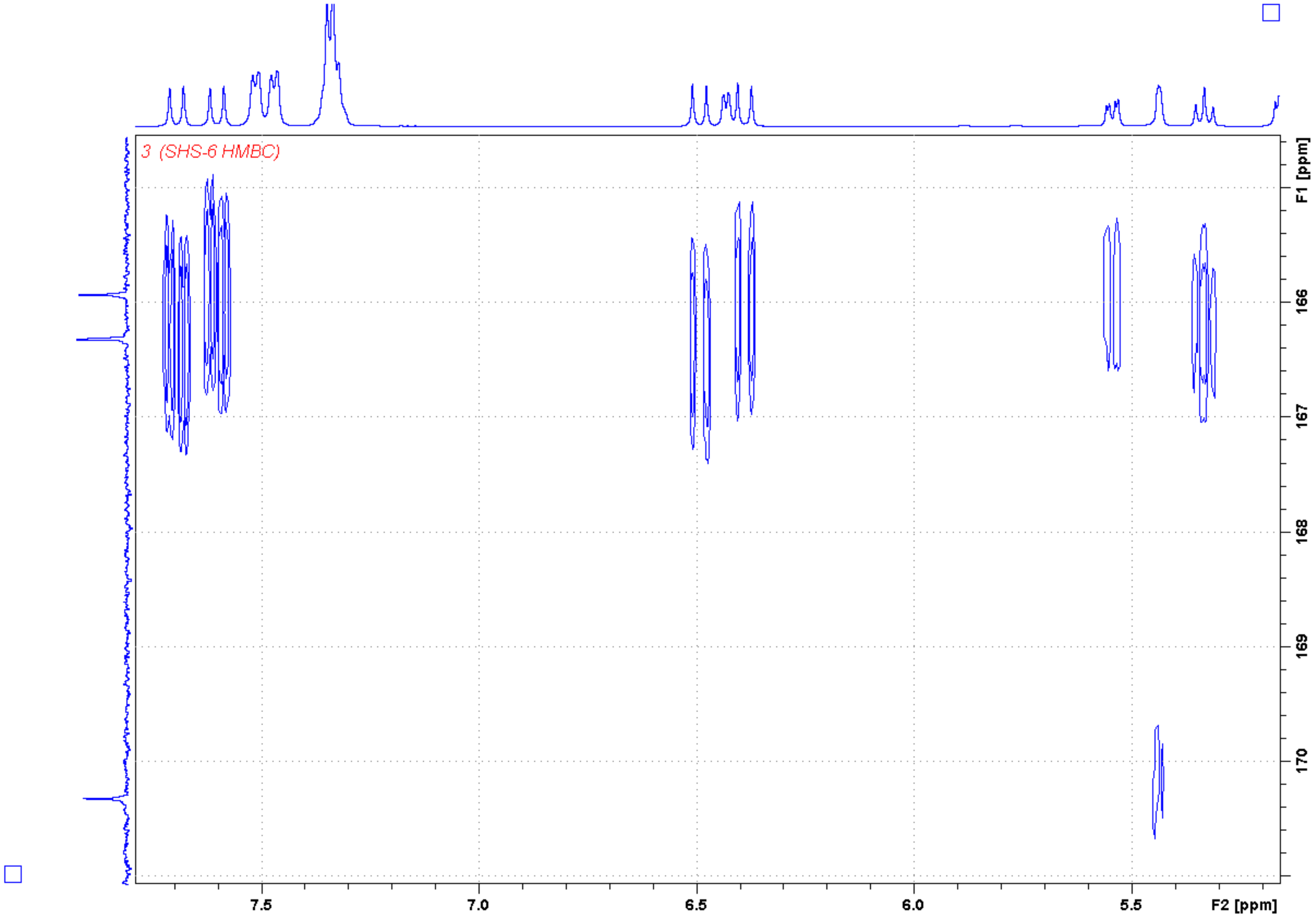


**Figure S60.** HMBC spectrum of compound 3 (expansion).

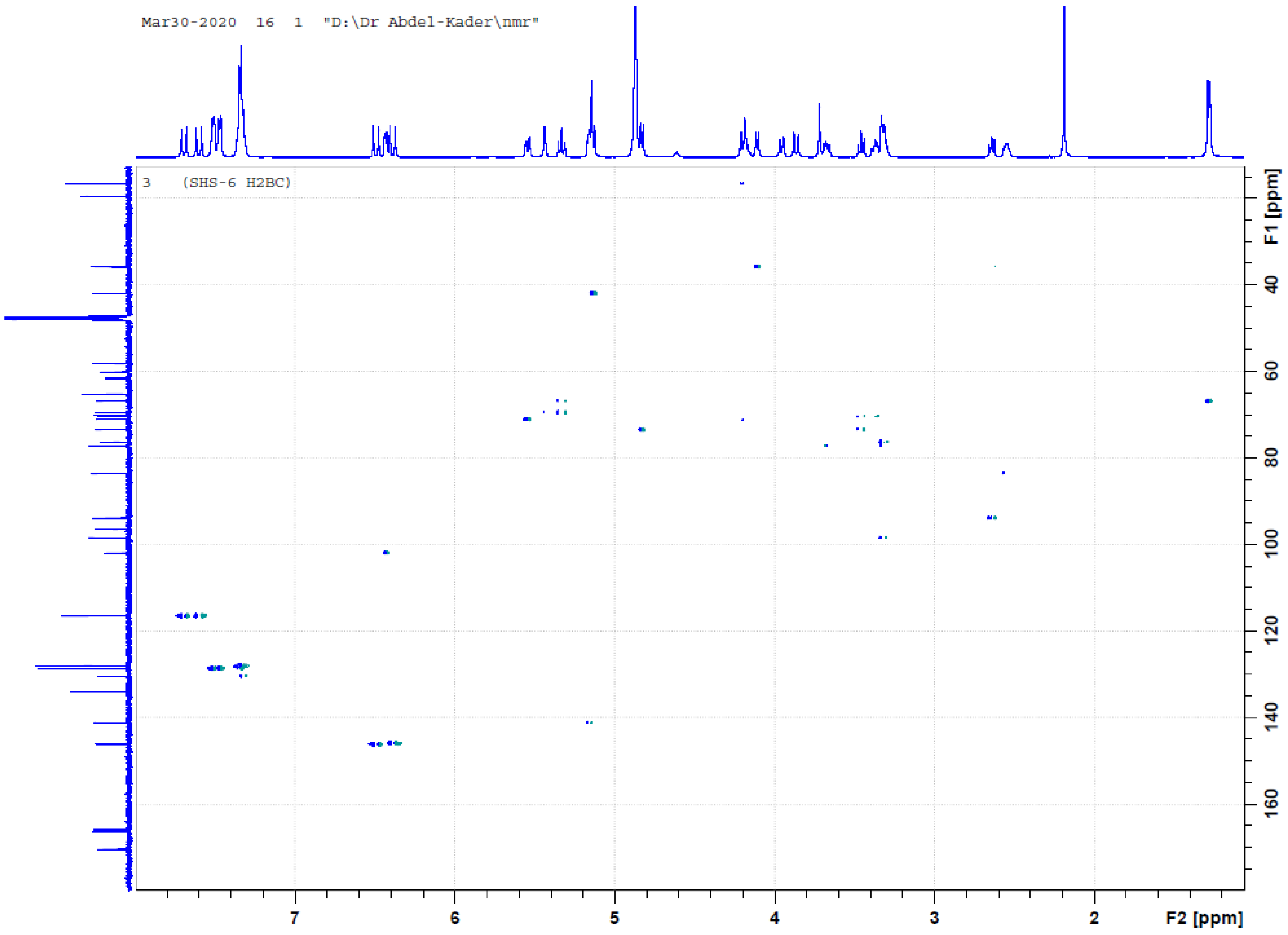


**Figure S61.** H2BC spectrum of compound 3.

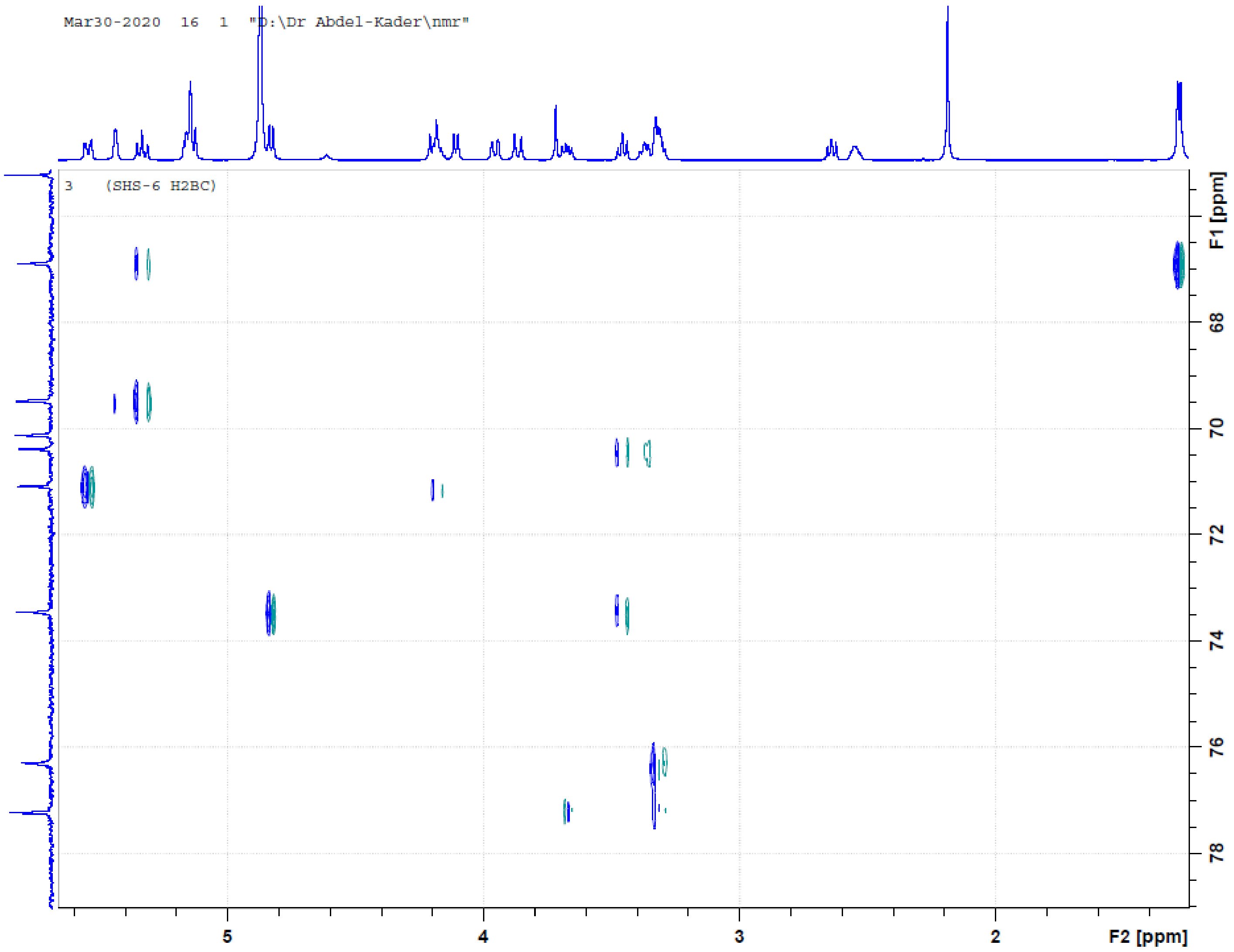


**Figure S62** H2BC spectrum of compound 3 (expansion).

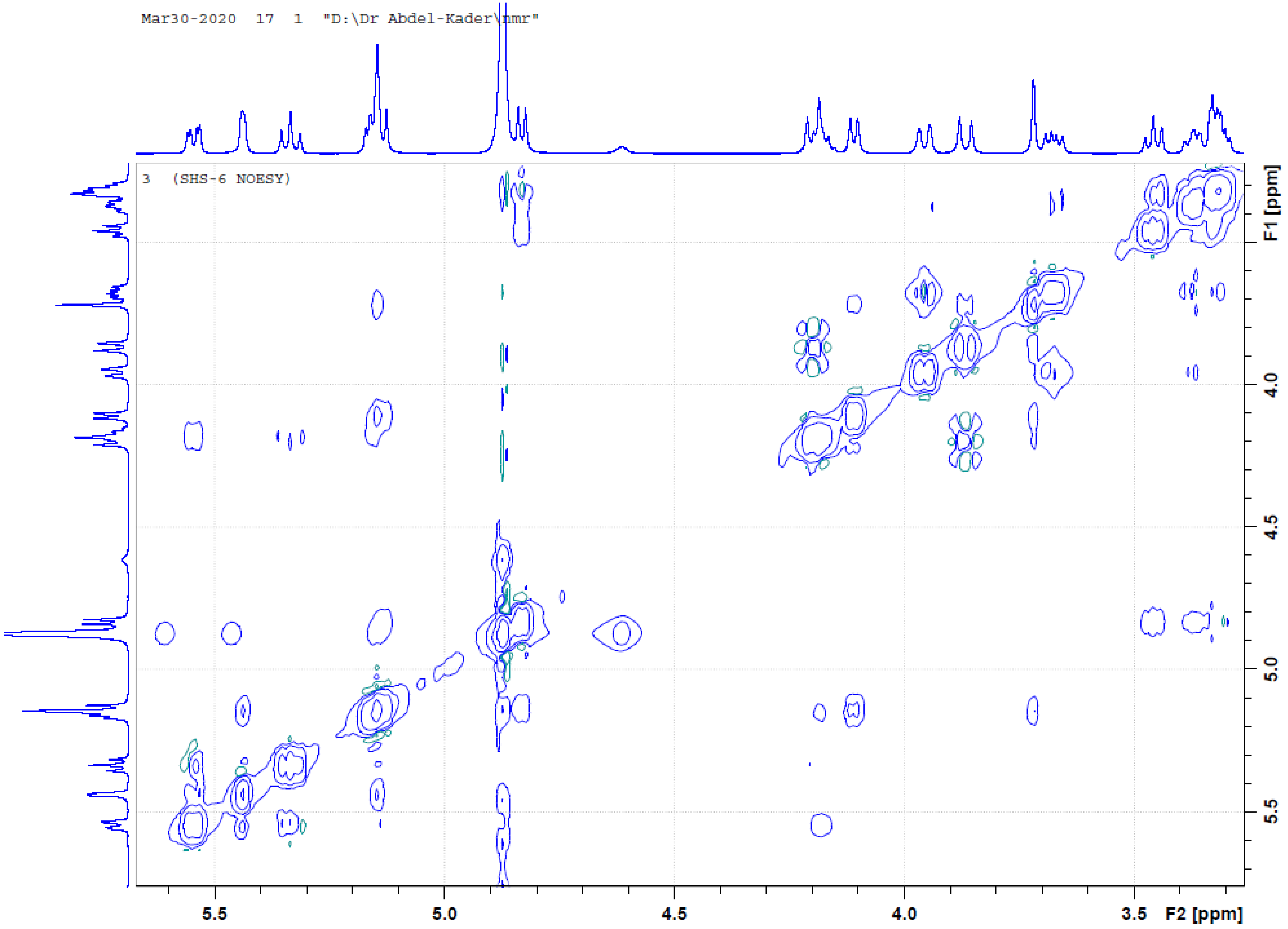


**Figure S63.** NOESY spectrum of compound 3 (expansion).

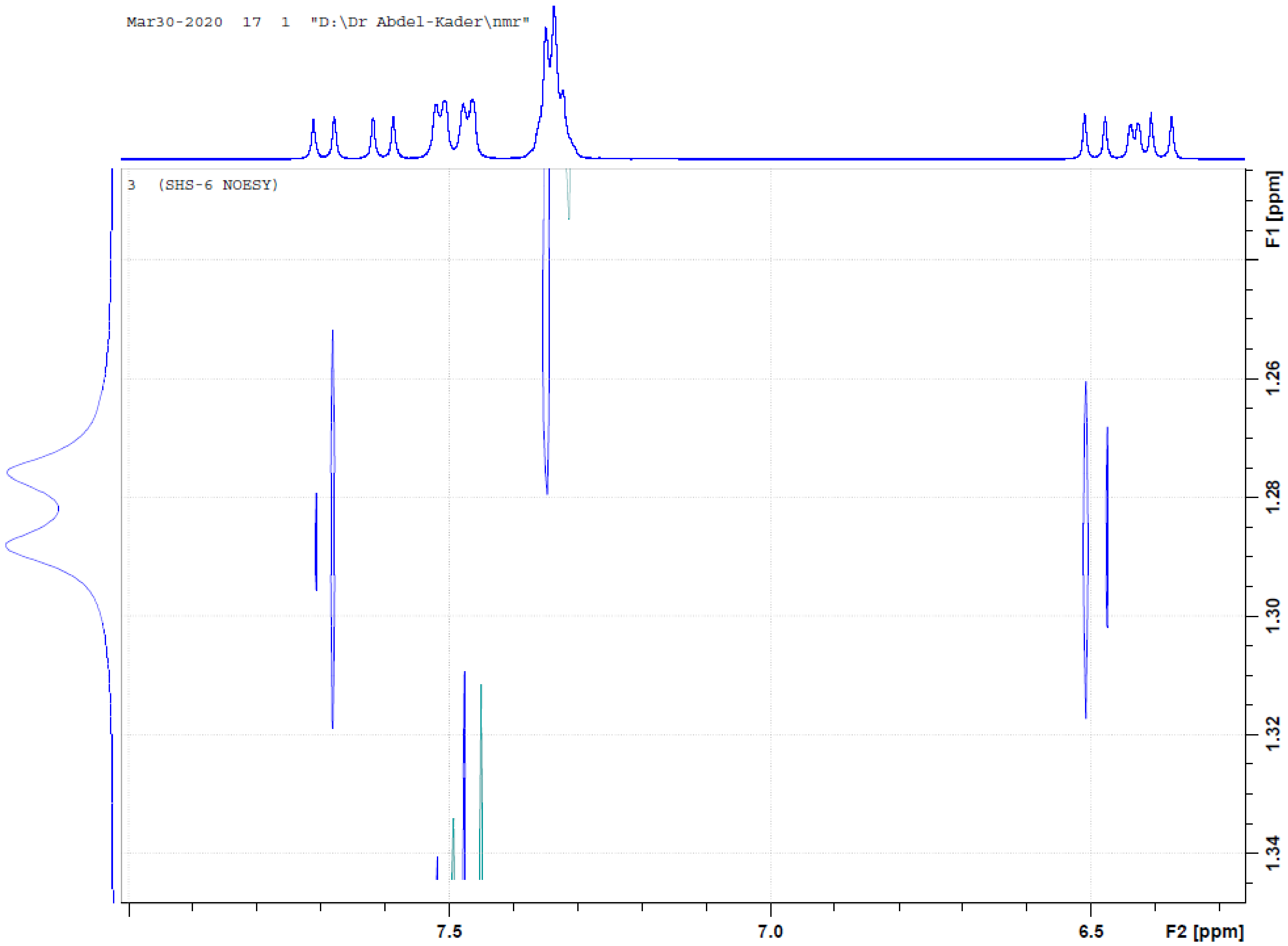


**Figure S64.** NOESY spectrum of compound 3 (expansion).

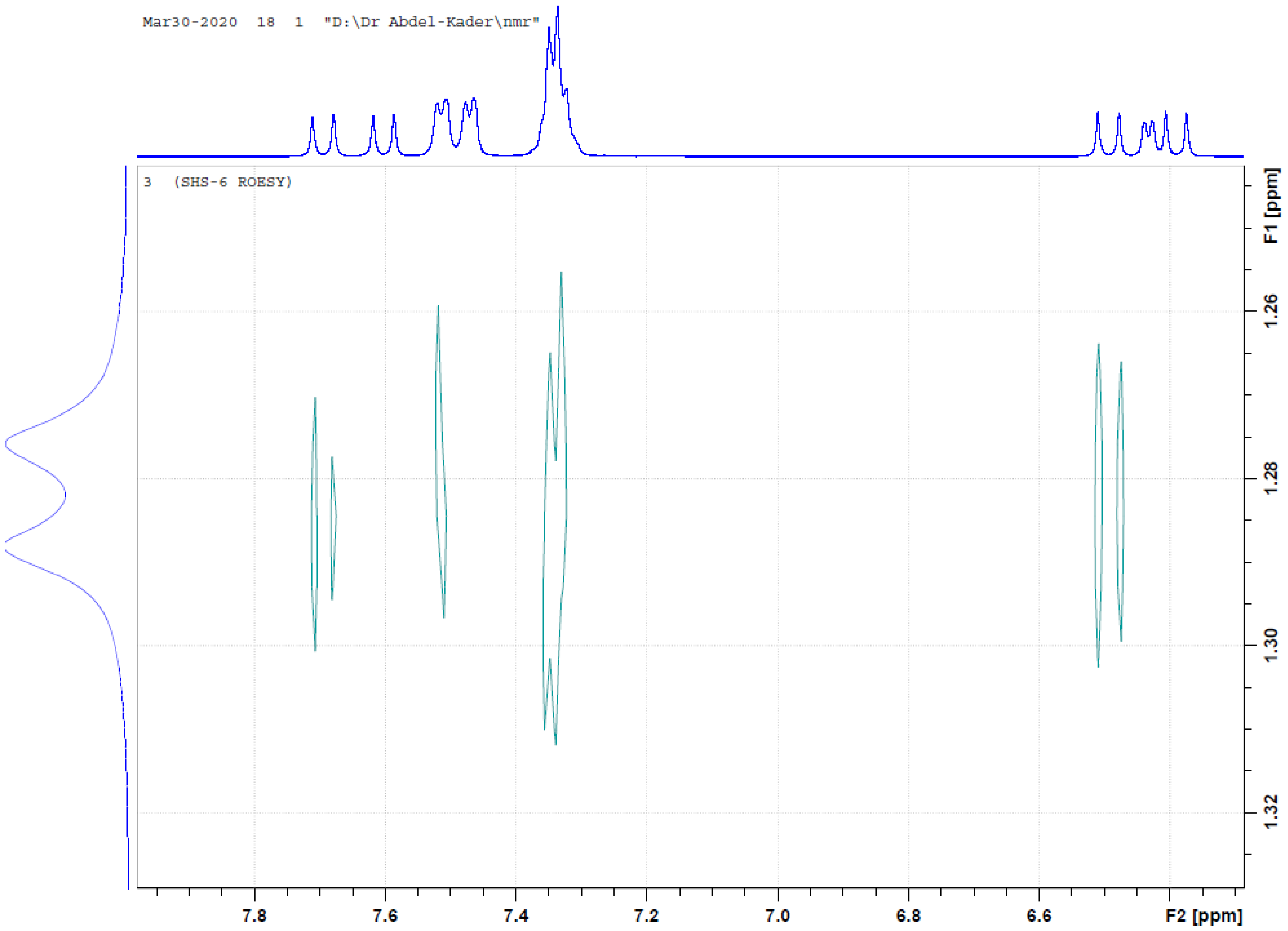


**Figure S65.** ROESY spectrum of compound 3 (expansion).

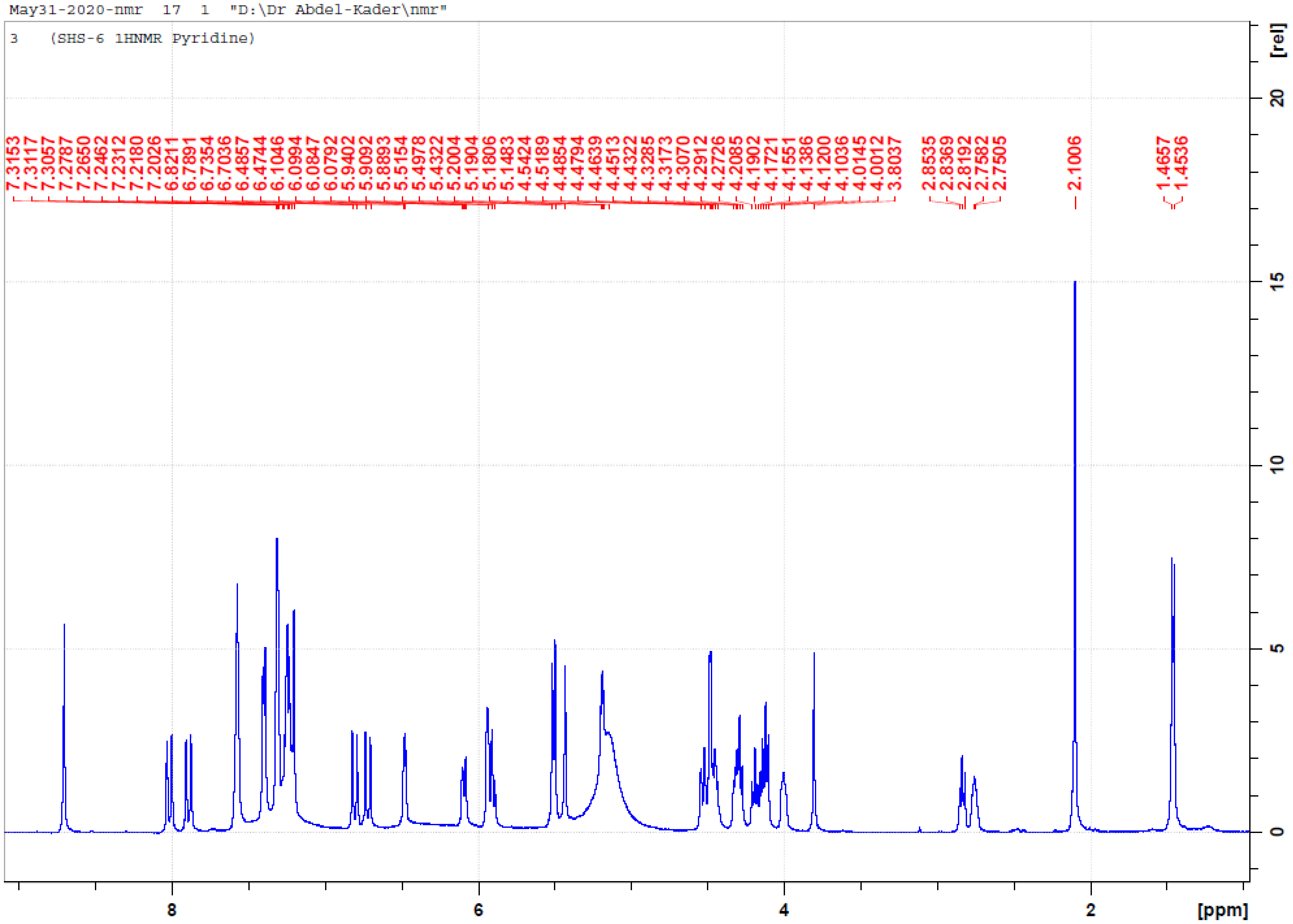


**Figure S66.** $^{1}$HNMR spectrum of compound 3 in Pyridine.

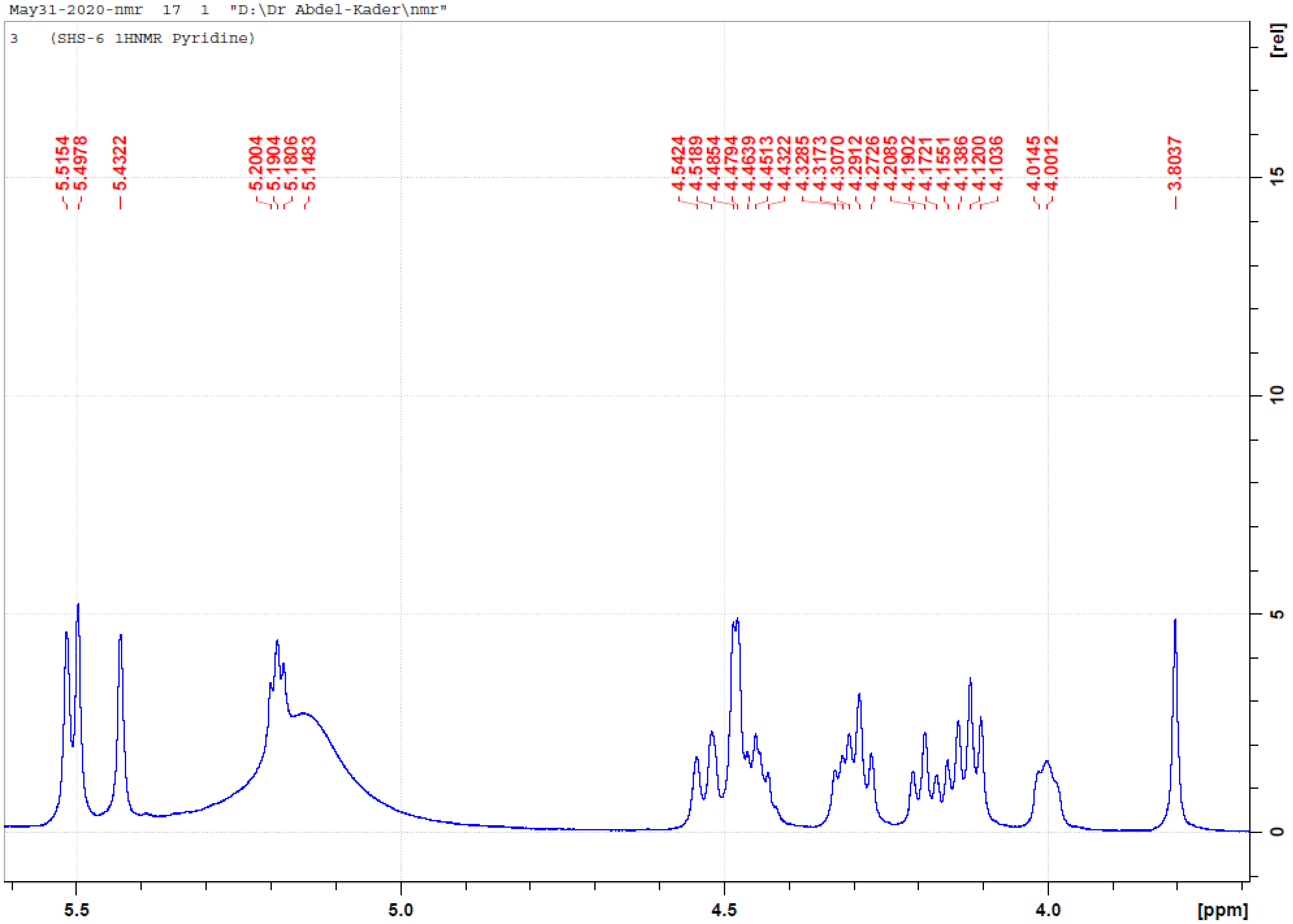


**Figure S67.** $^{1}$HNMR spectrum of compound 3 in Pyridine (expansion).

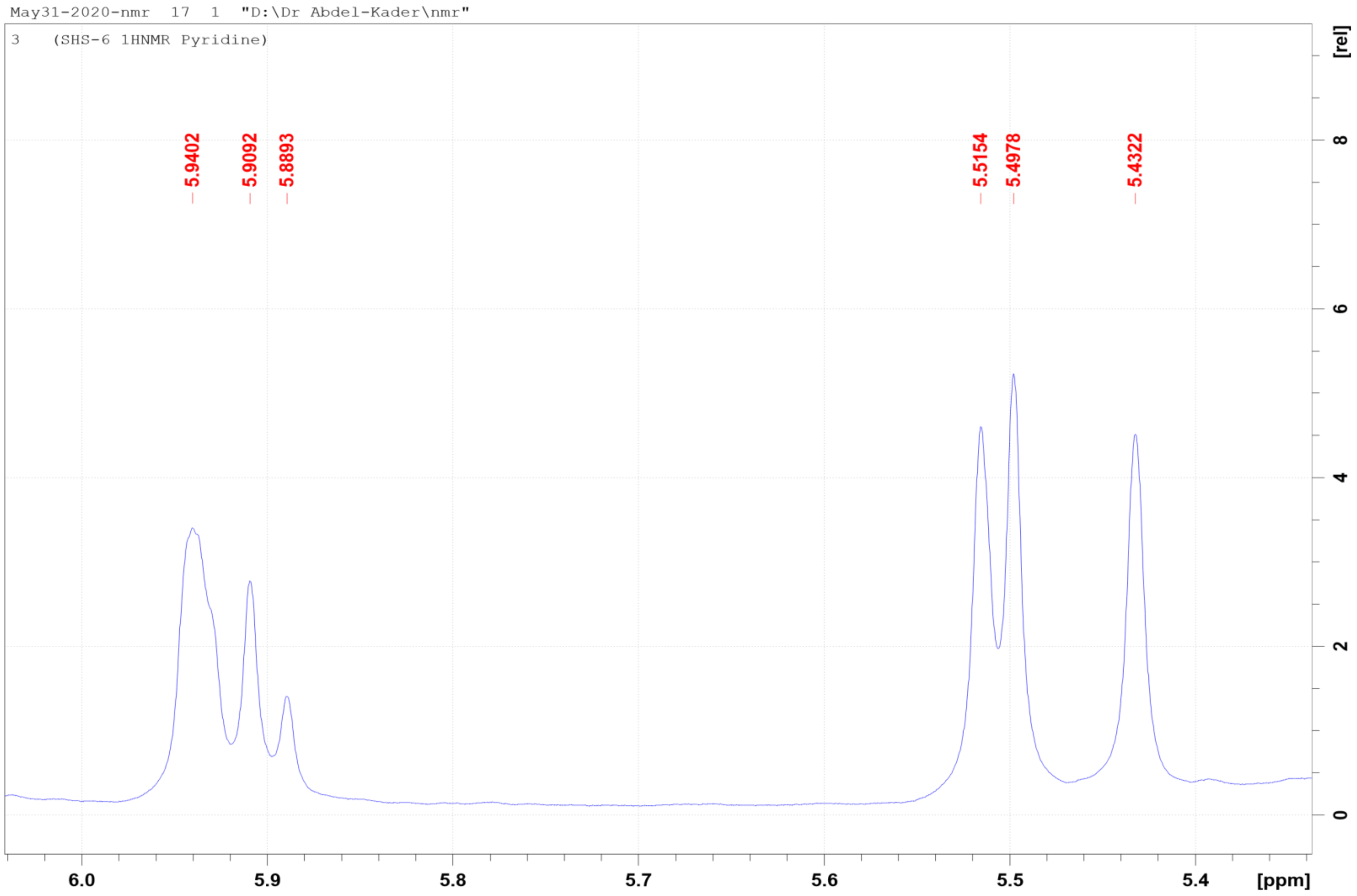


**Figure S68.** $^{1}$HNMR spectrum of compound 3 in Pyridine (expansion).

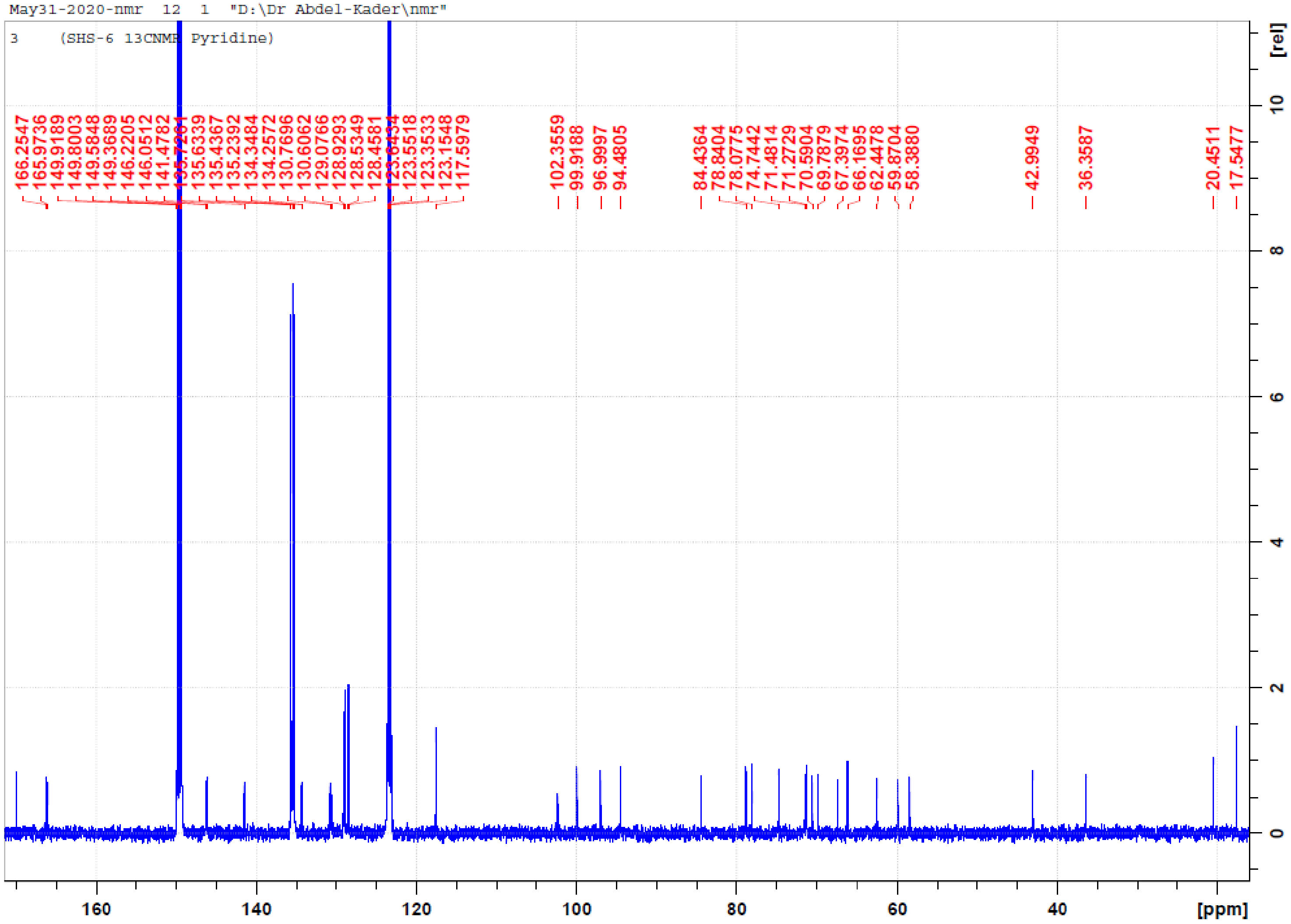


**Figure S69.** $^{13}$CNMR spectrum of compound 3 in Pyridine.

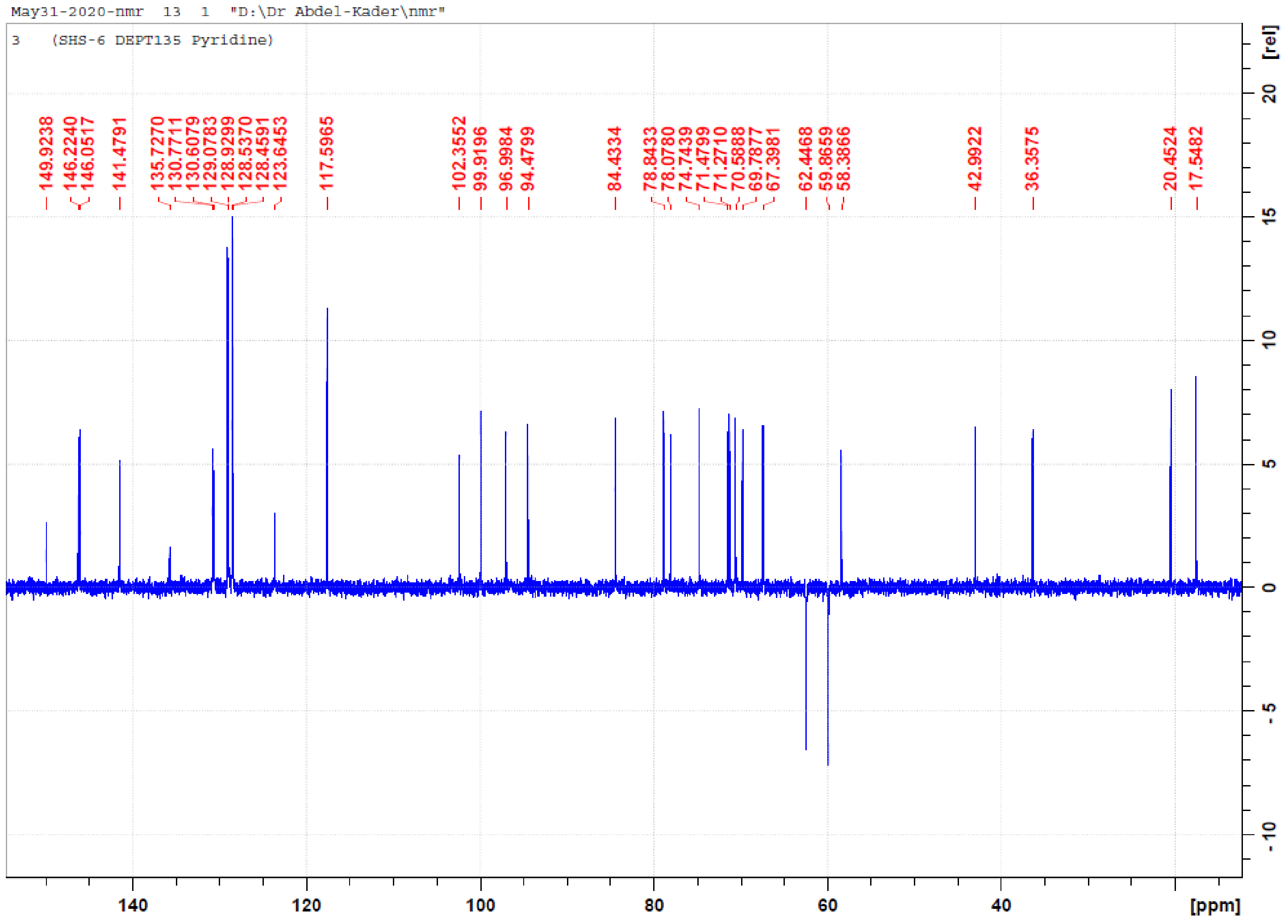


**Figure S70.** DEPT135 spectrum of compound 3 in Pyridine.

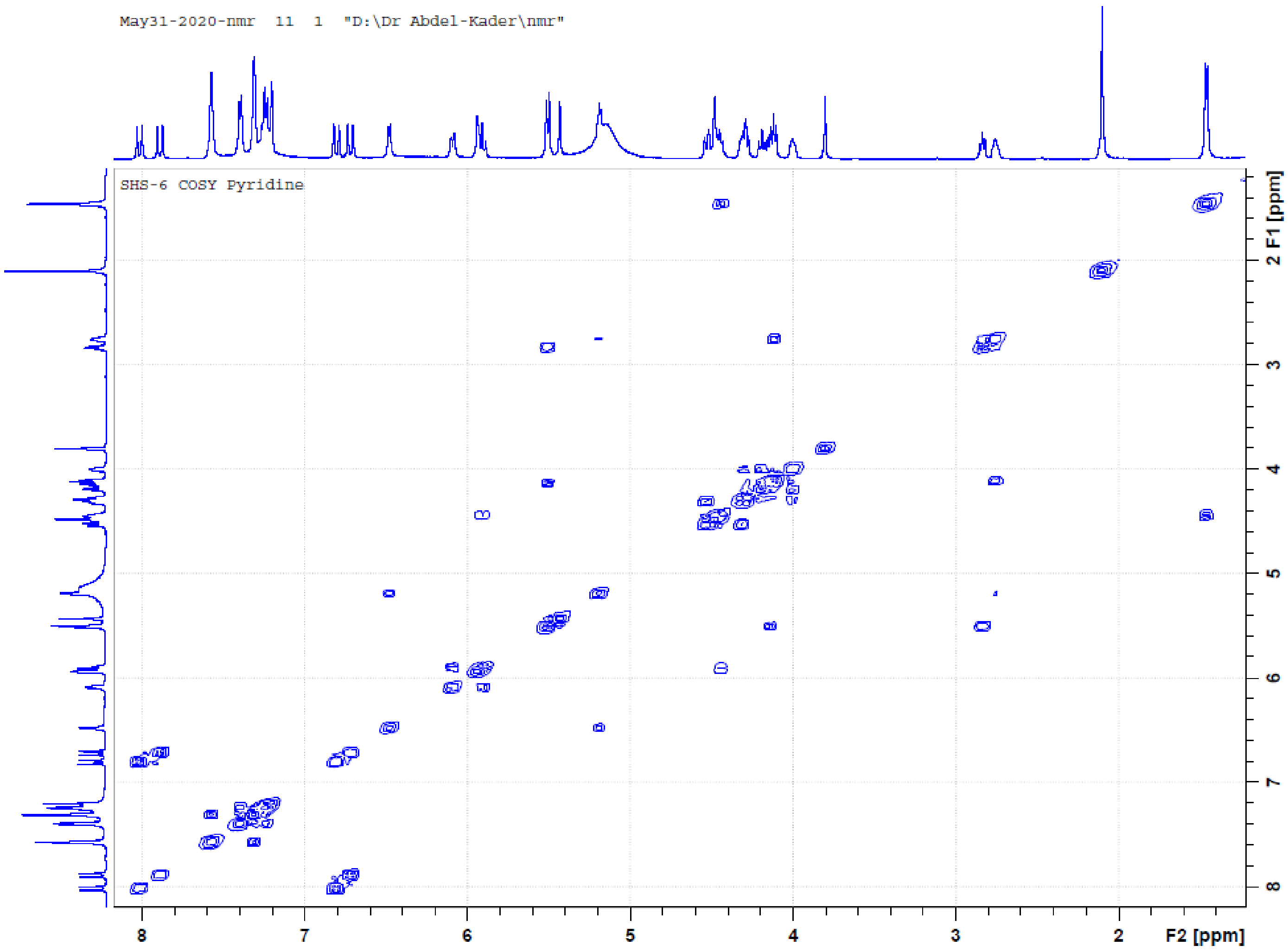


**Figure S71.** COSY spectrum of compound 3 in Pyridine.

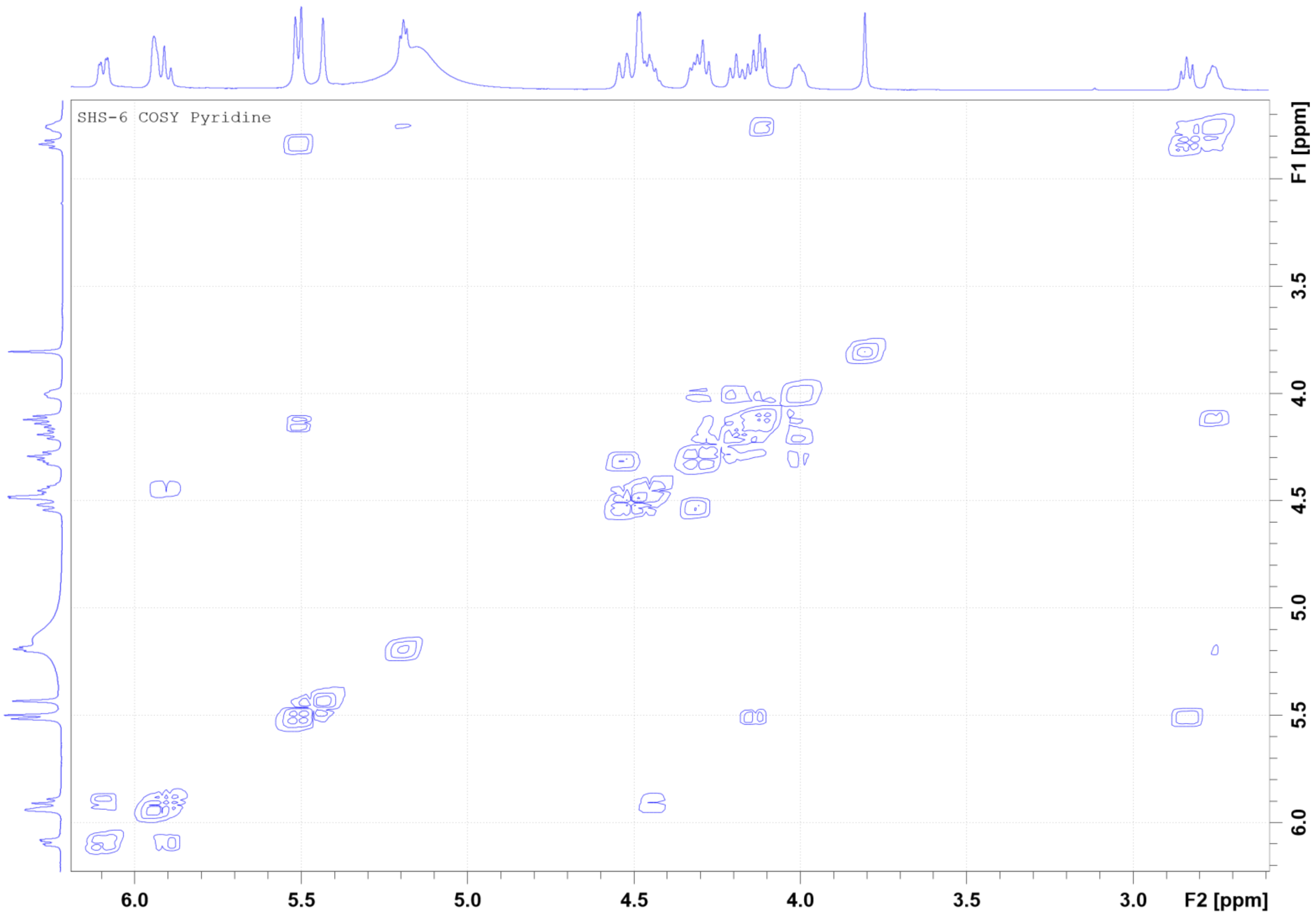


**Figure S72.** COSY spectrum of compound 3 in Pyridine (expansion).

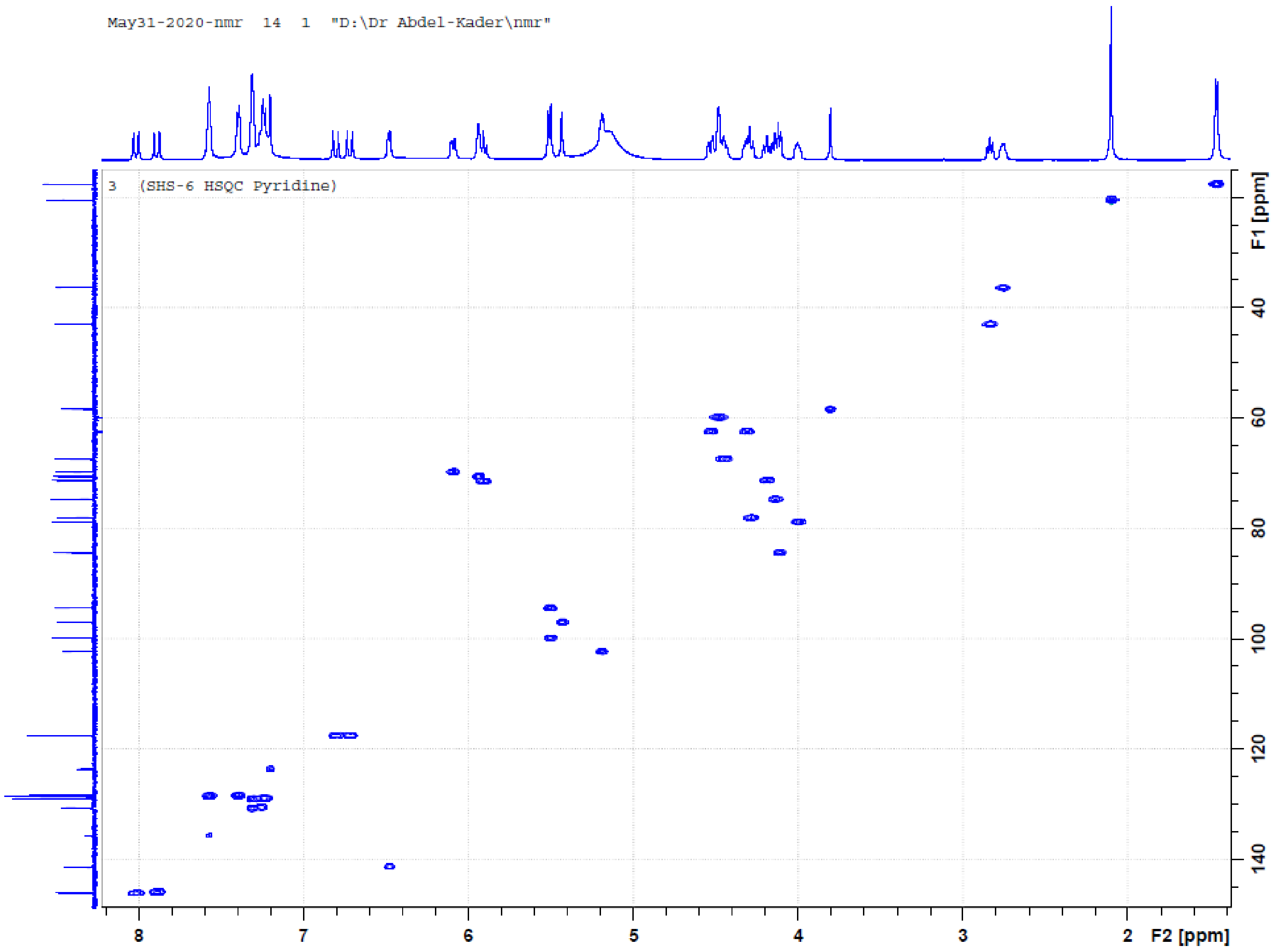


**Figure S73.** HSQC spectrum of compound 3 in Pyridine.

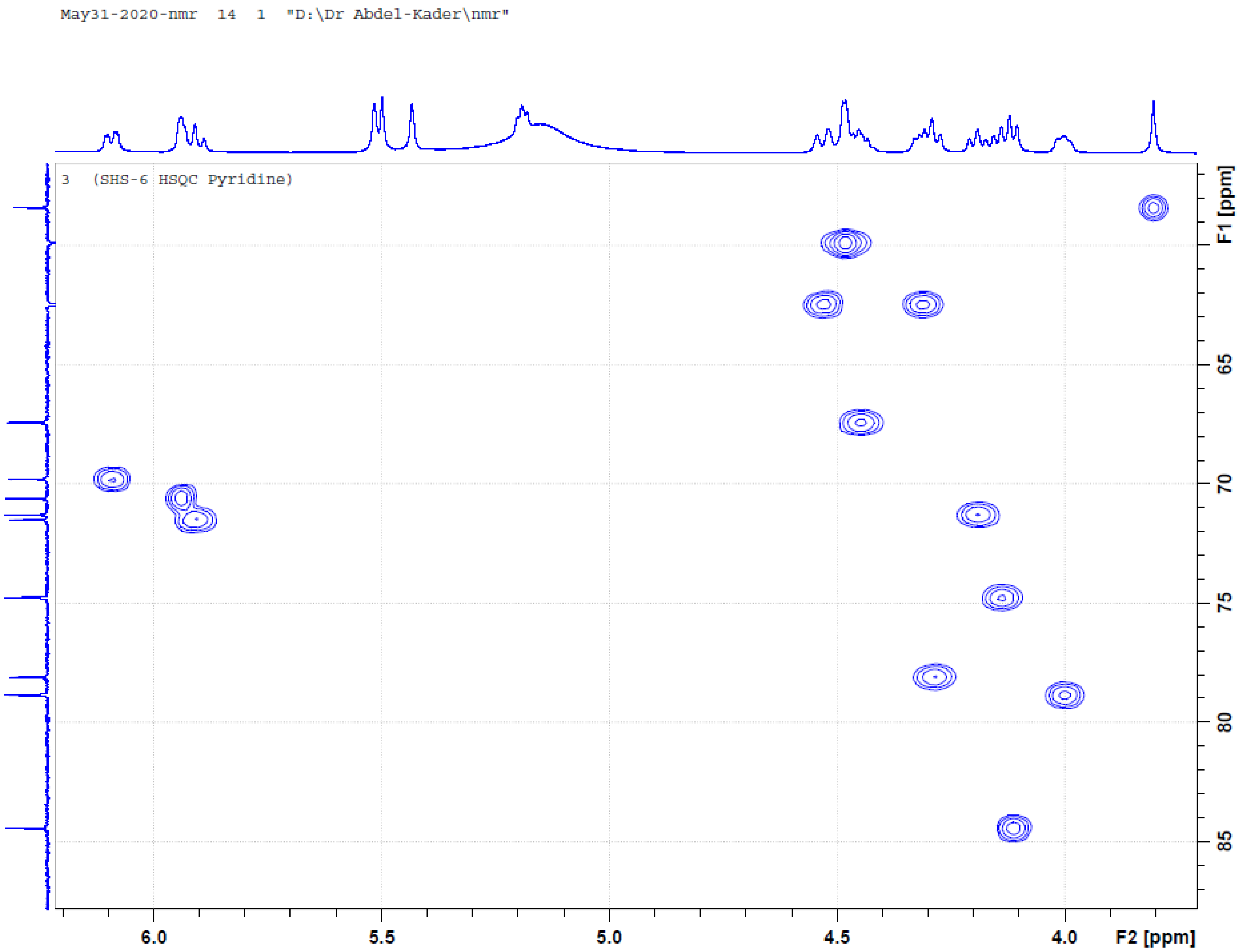


**Figure S74.** HSQC spectrum of compound 3 in Pyridine (expansion).

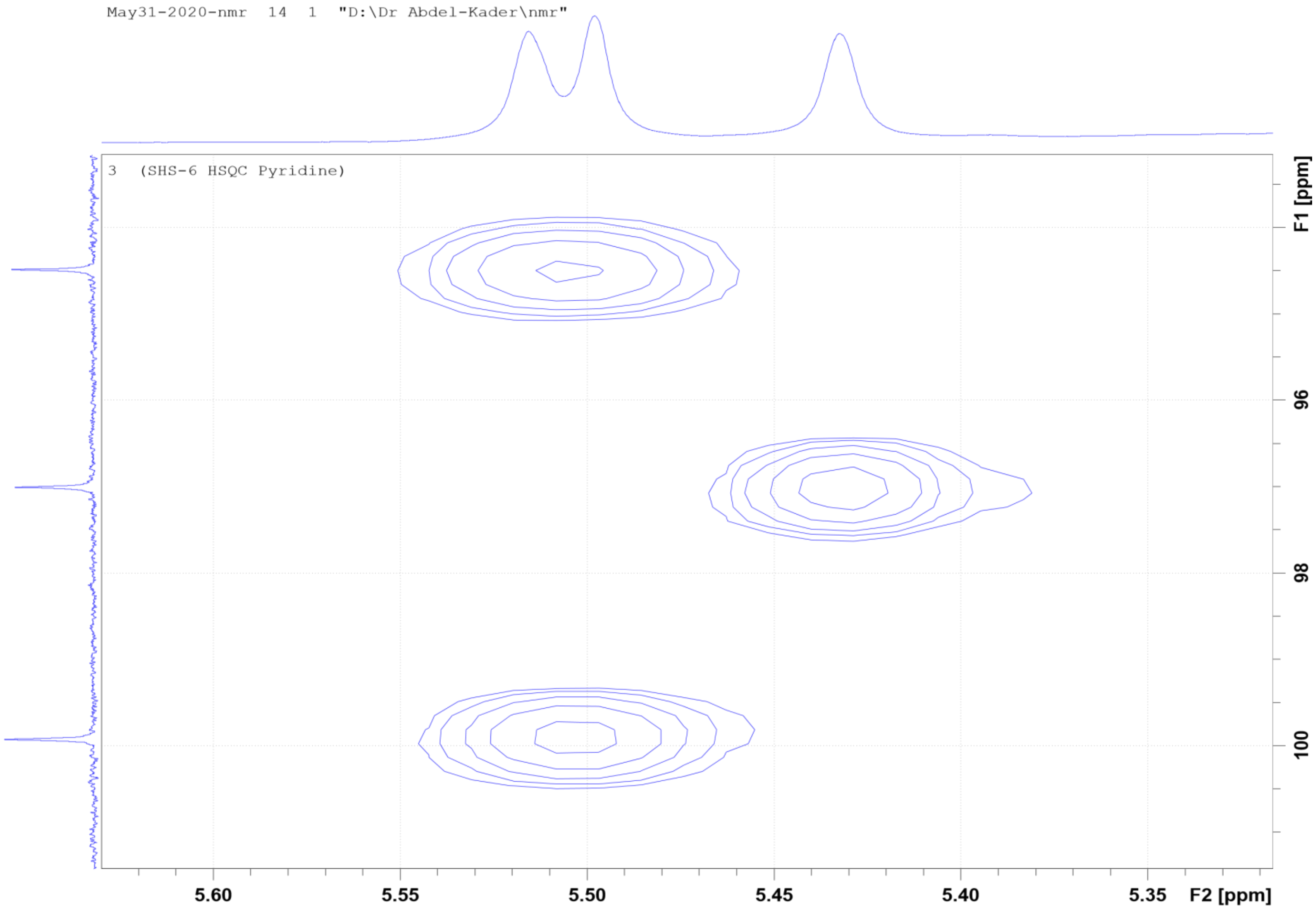


**Figure S75.** HSQC spectrum of compound 3 in Pyridine (expansion).

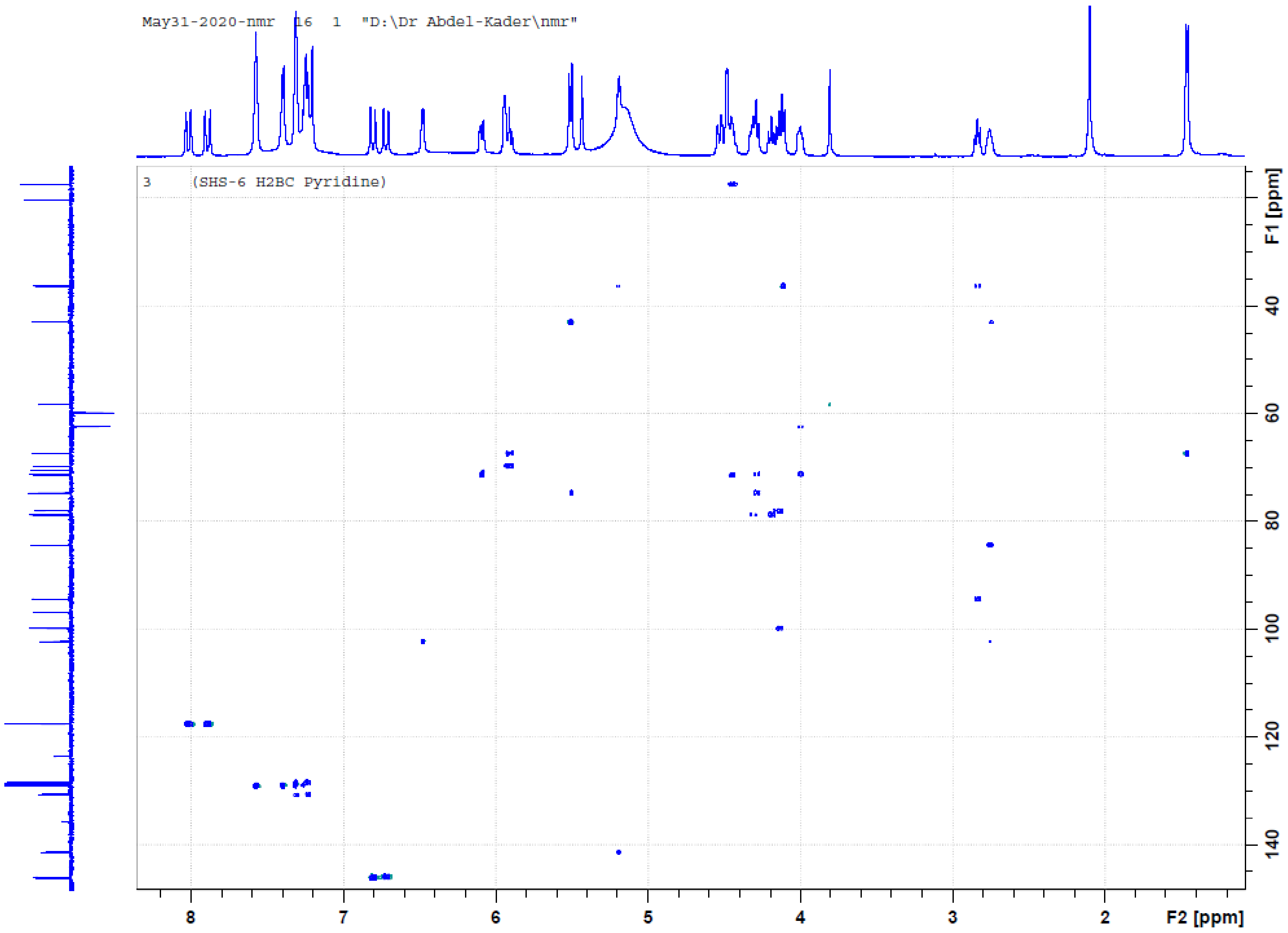


**Figure S76.** H2BC spectrum of compound 3 in Pyridine.

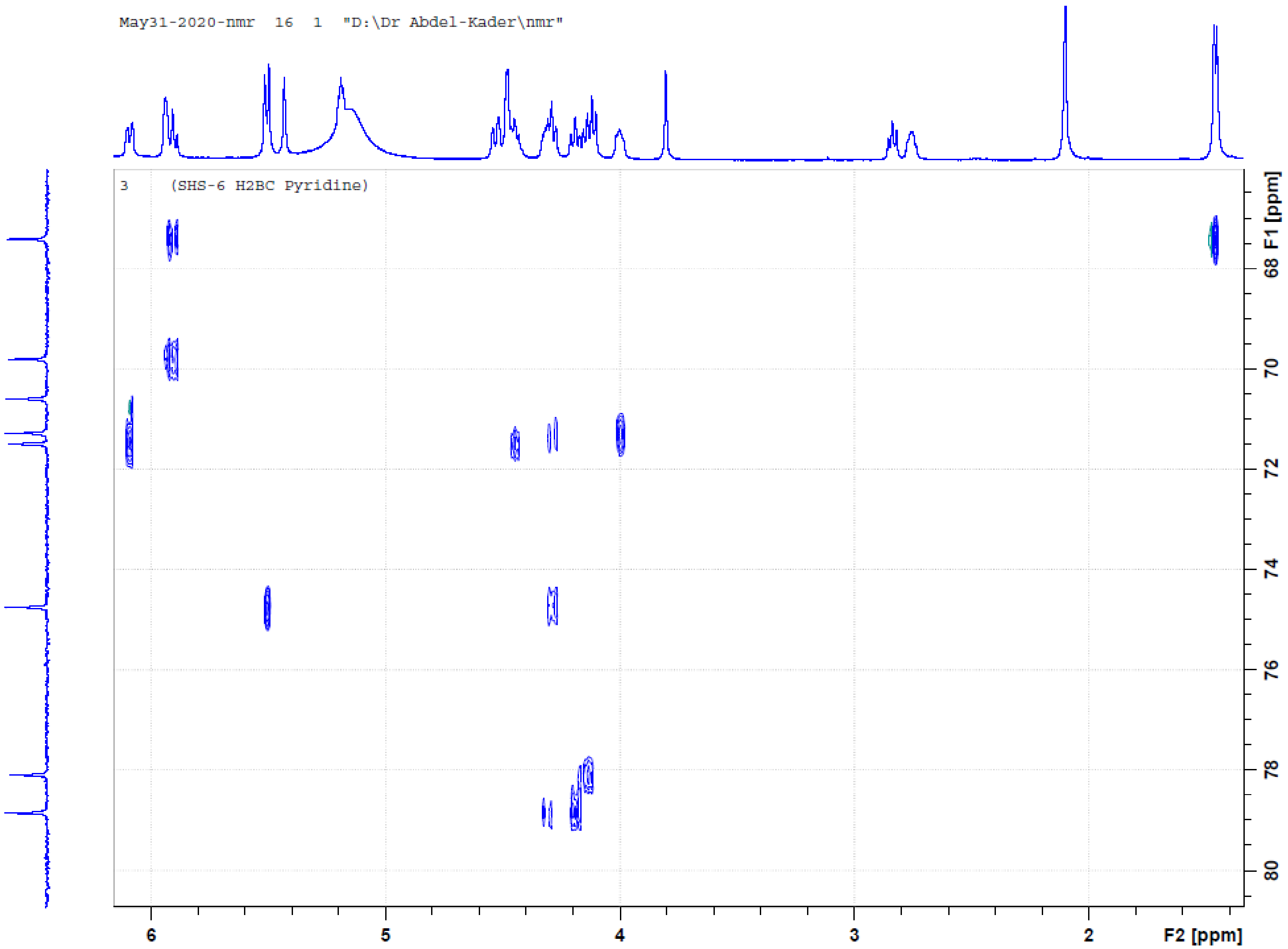


**Figure S77.** H2BC spectrum of compound 3 in Pyridine (expansion).

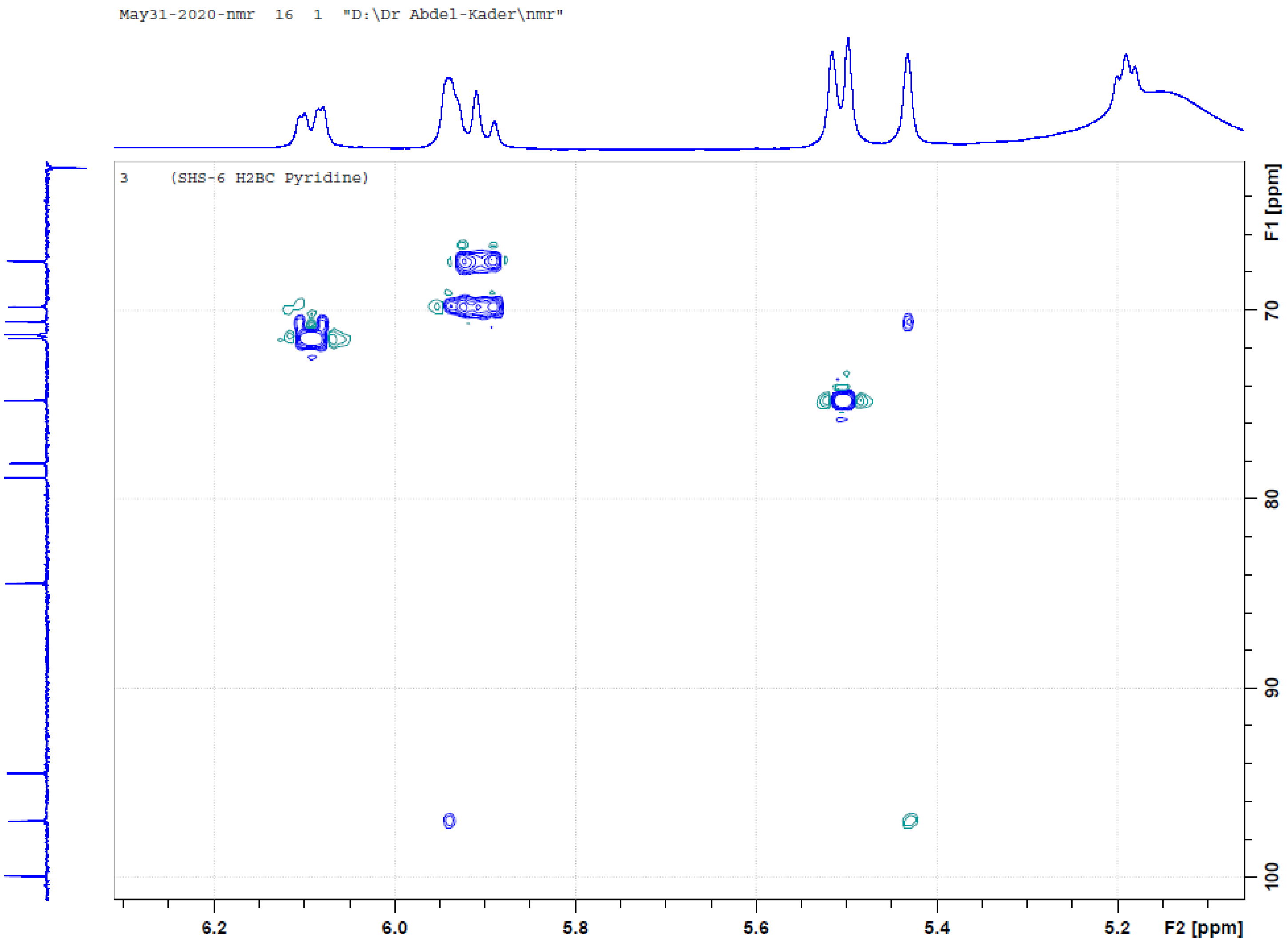


**Figure S78.** H2BC spectrum of compound 3 in Pyridine (expansion).

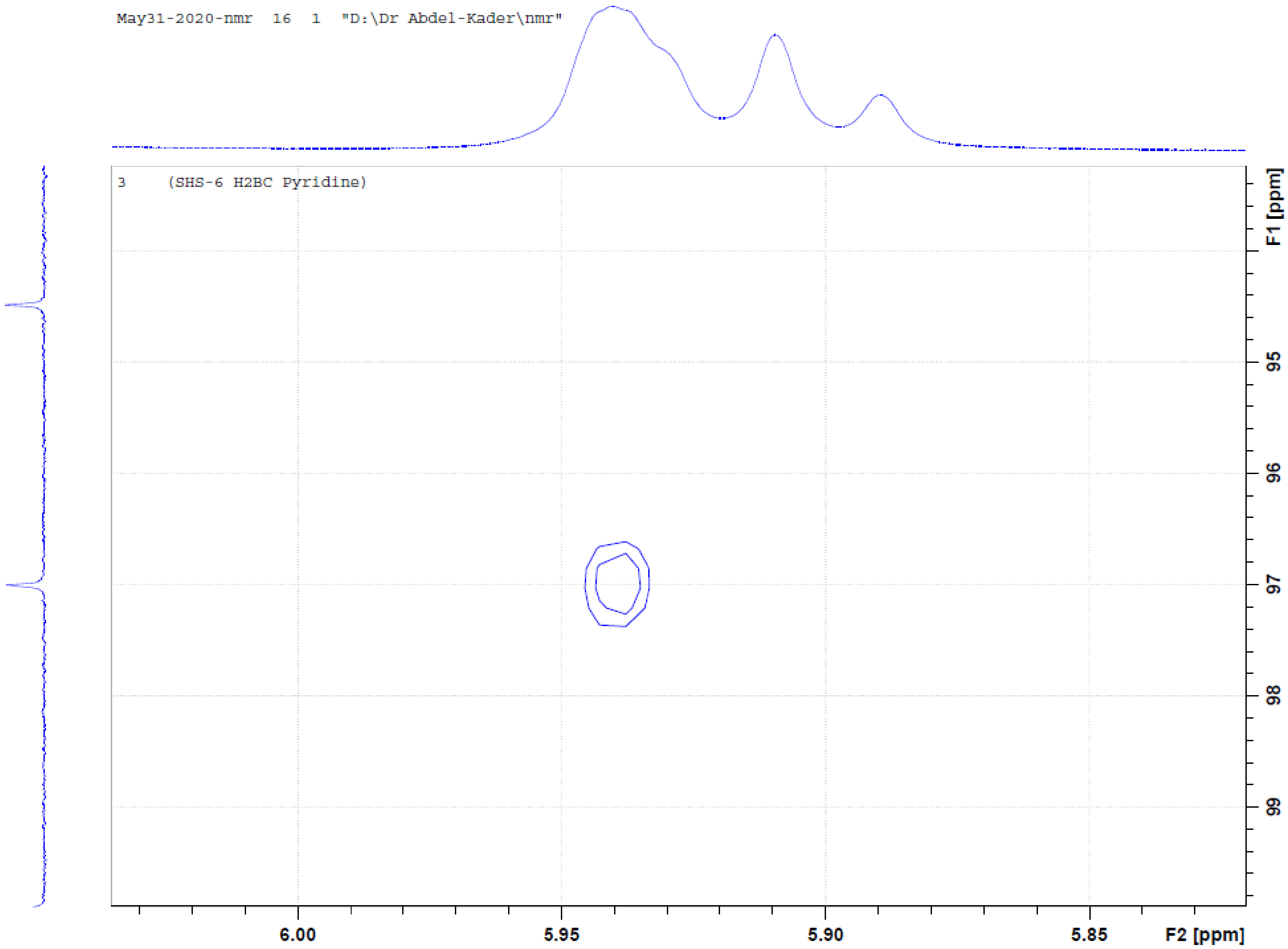


**Figure S79.** H2BC spectrum of compound 3 in Pyridine (expansion).

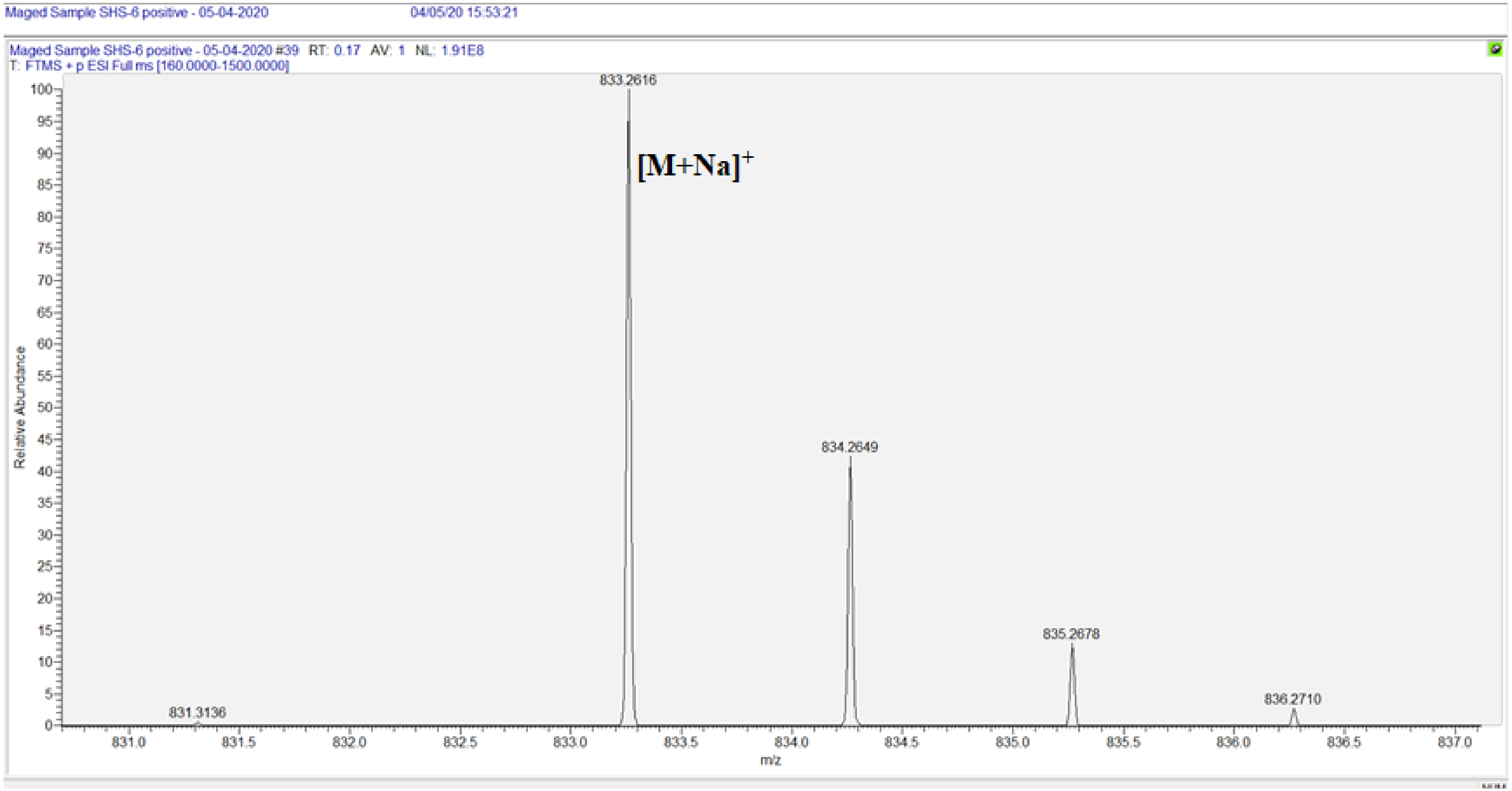


**Figure S80.** HRESIMS of compound 3 (positive mode).

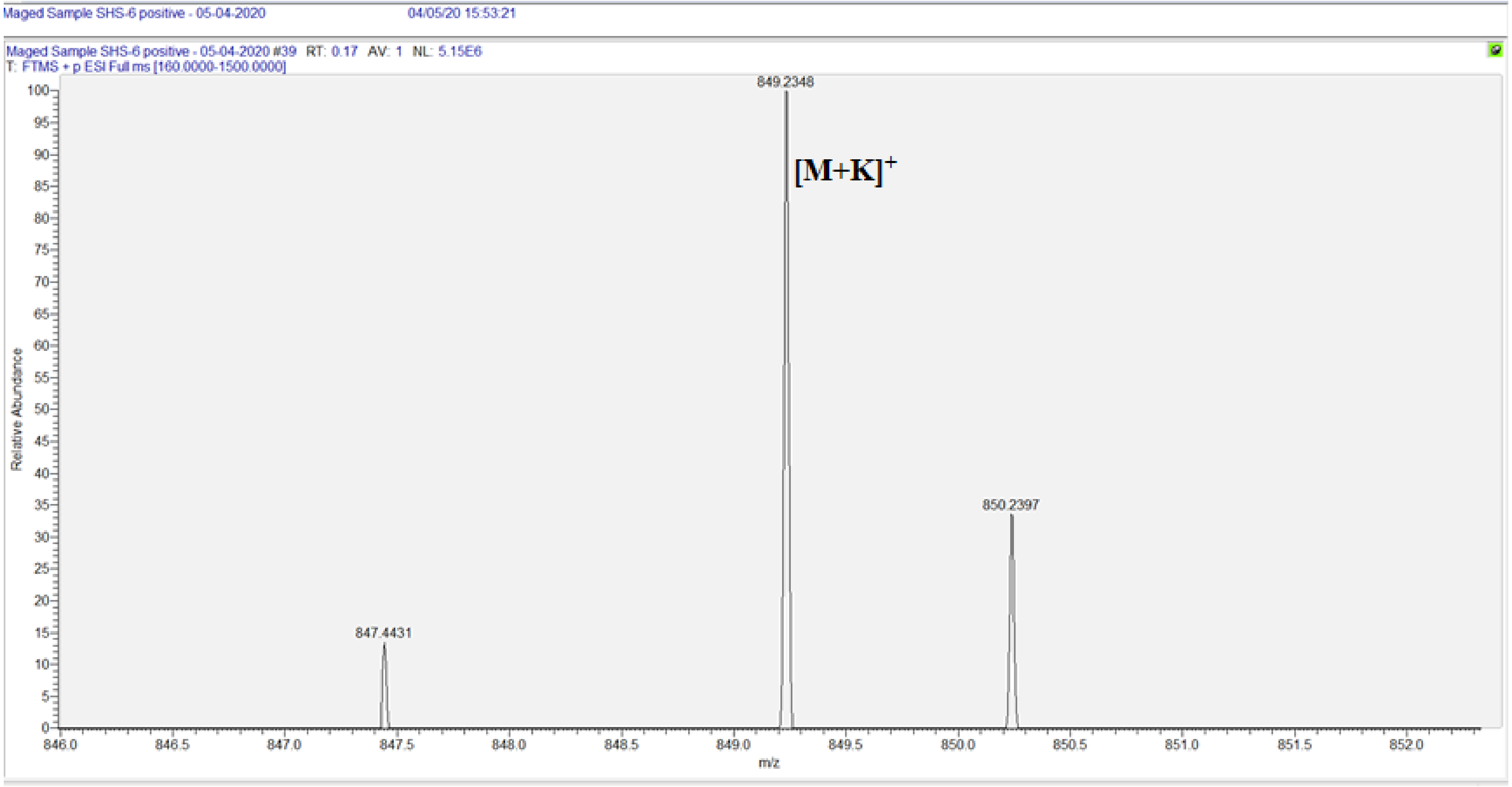


**Figure S81.** HRESIMS of compound 3 (positive mode).

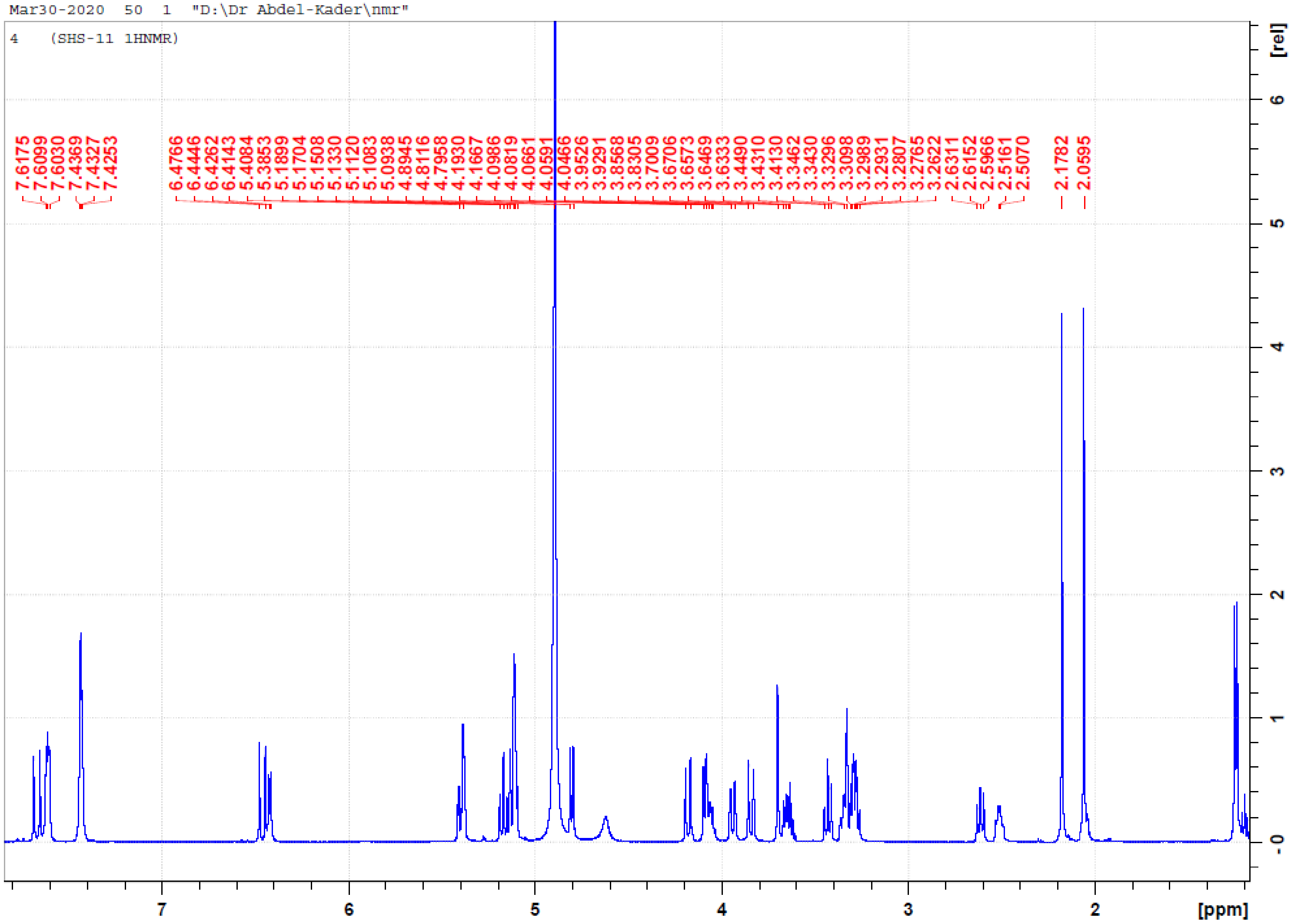


**Figure S82.** $^{1}$HNMR spectrum of compound 4.

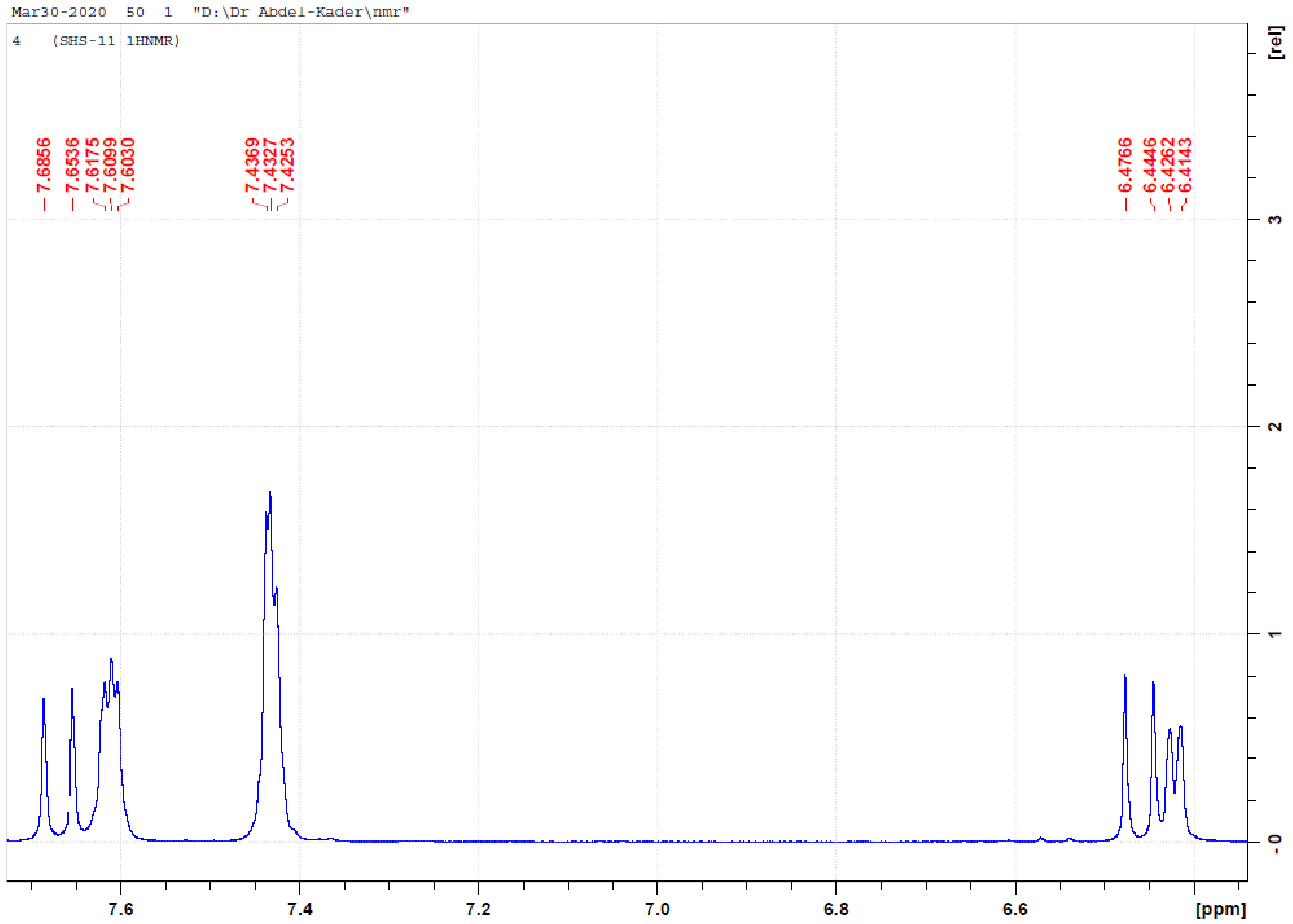


**Figure S83.** $^{1}$HNMR spectrum of compound 4 (expansion).

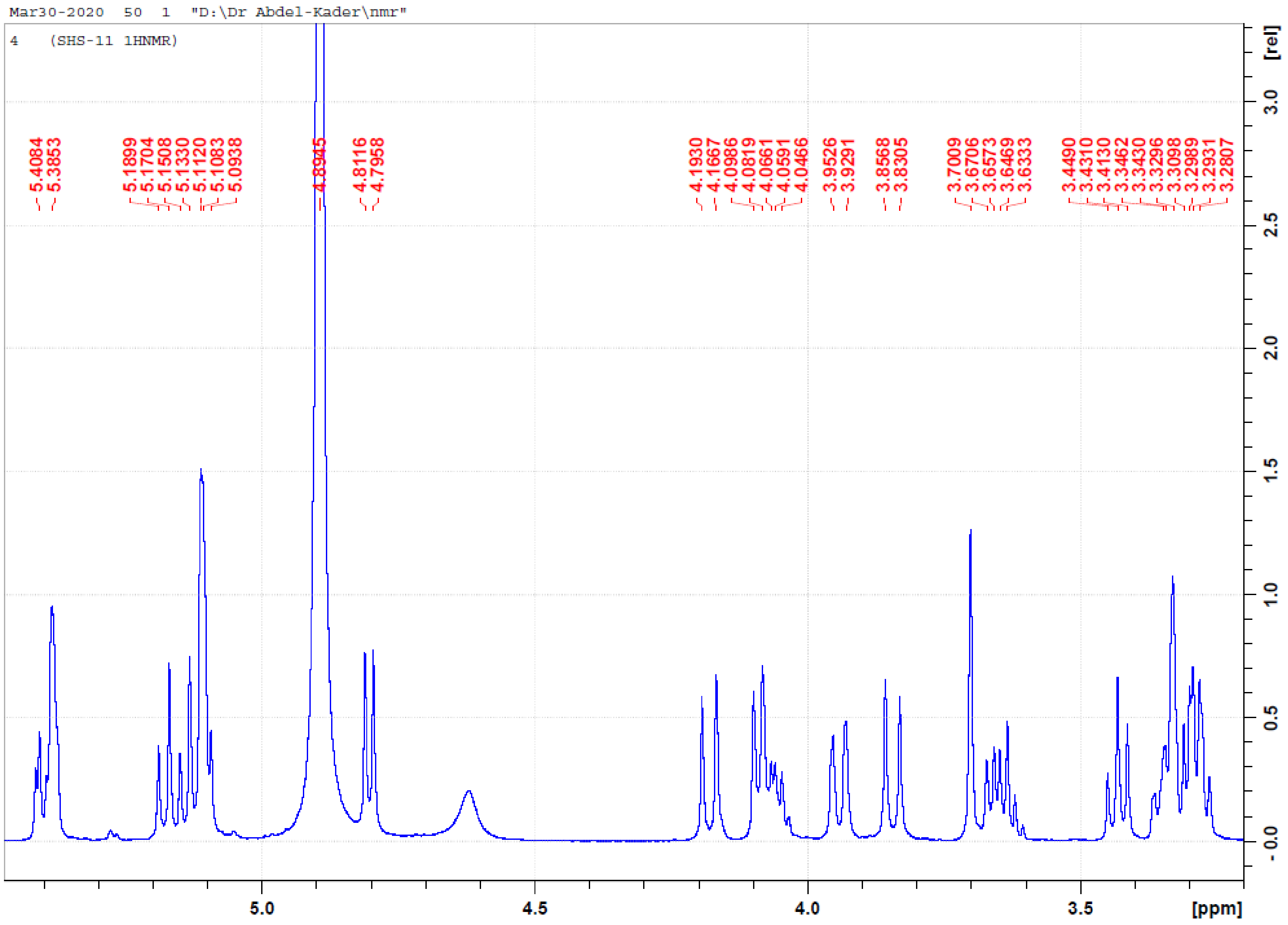


**Figure S84.** $^{1}$HNMR spectrum of compound 4 (expansion).

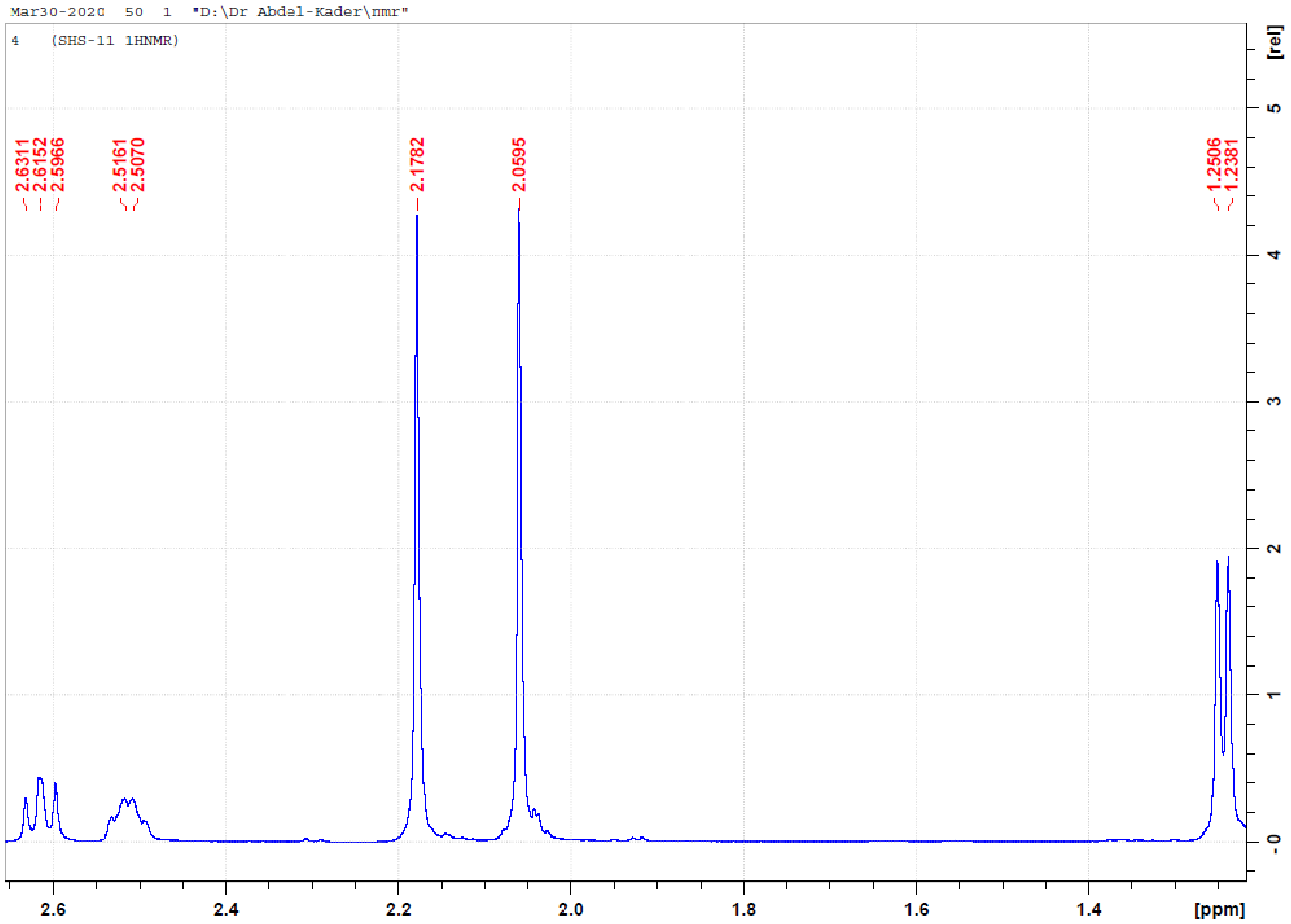


**Figure S85.** $^{1}$HNMR spectrum of compound 4 (expansion).

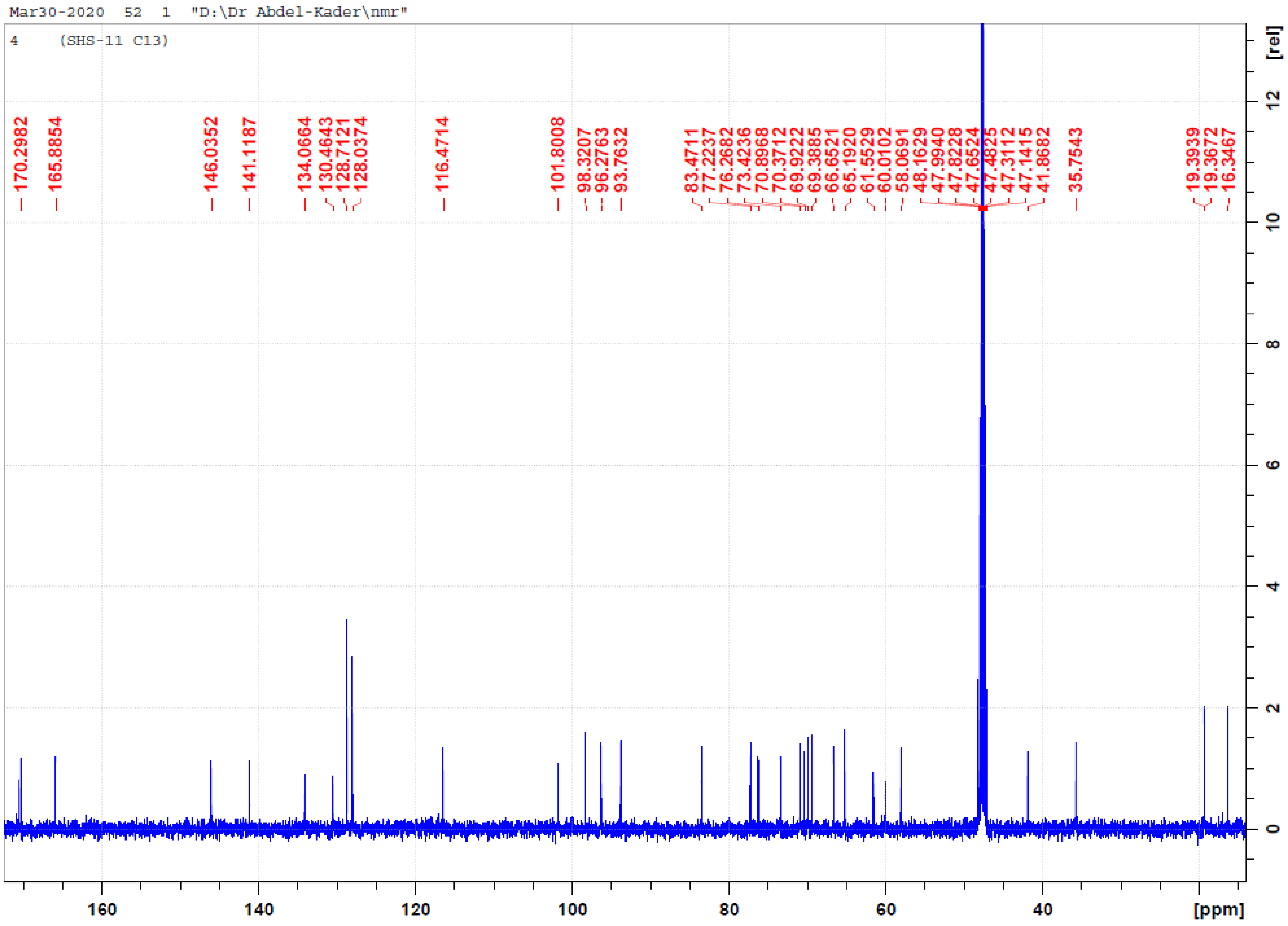


**Figure S86.** $^{13}$CNMR spectrum of compound 4.

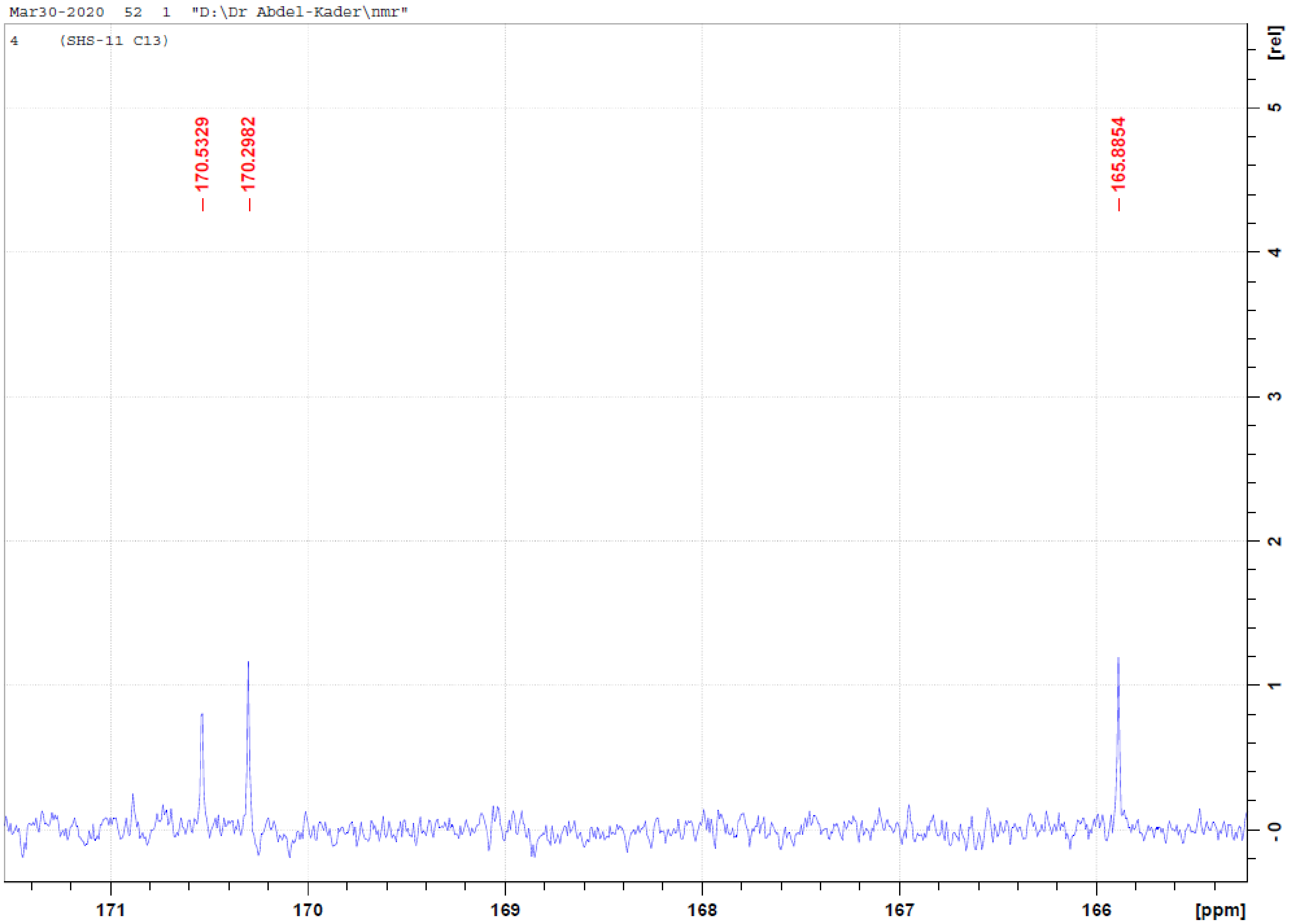


**Figure S87.** $^{13}$CNMR spectrum of compound 4 (expansion).

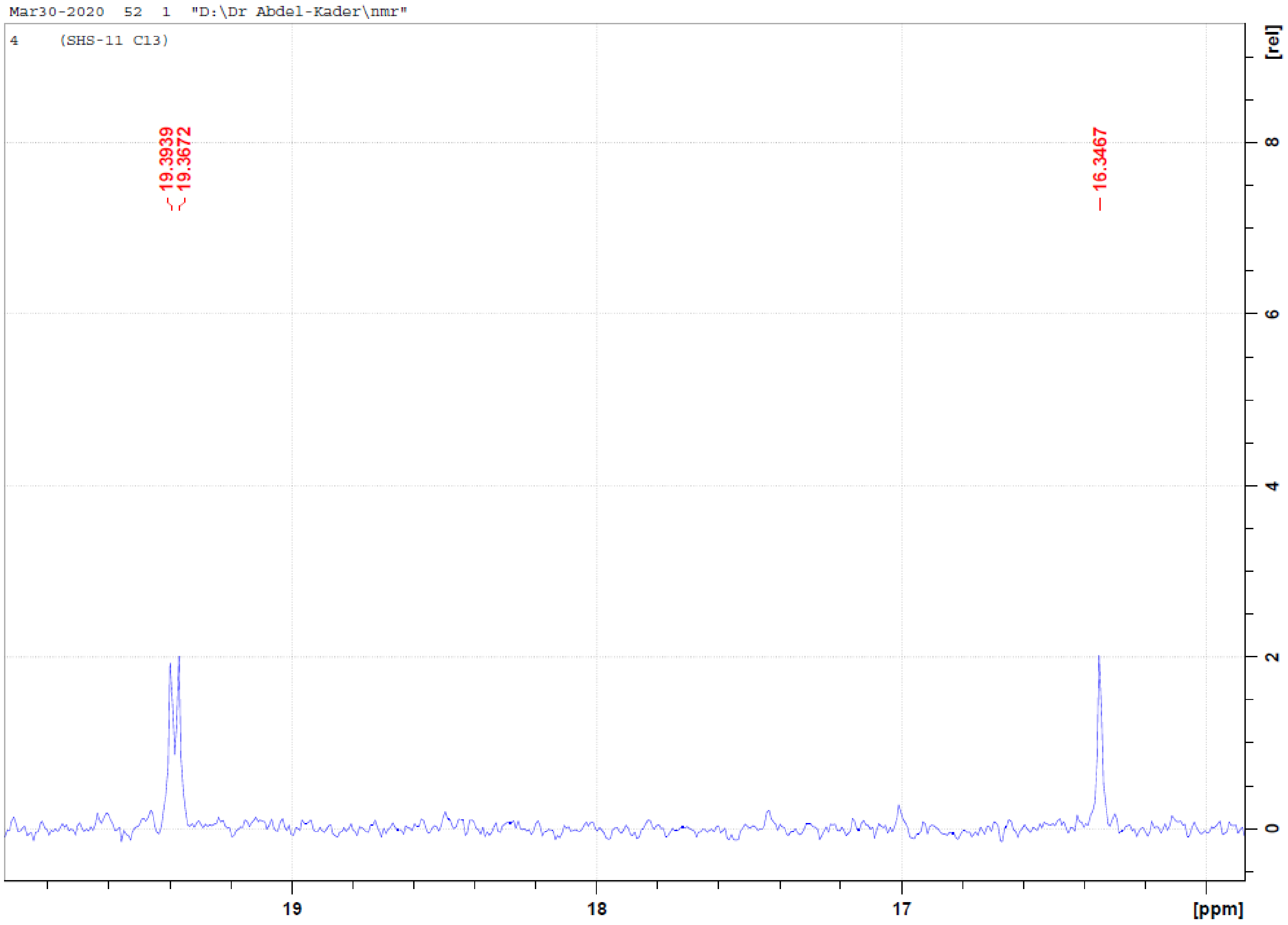


**Figure S88.** $^{13}$CNMR spectrum of compound 4 (expansion).

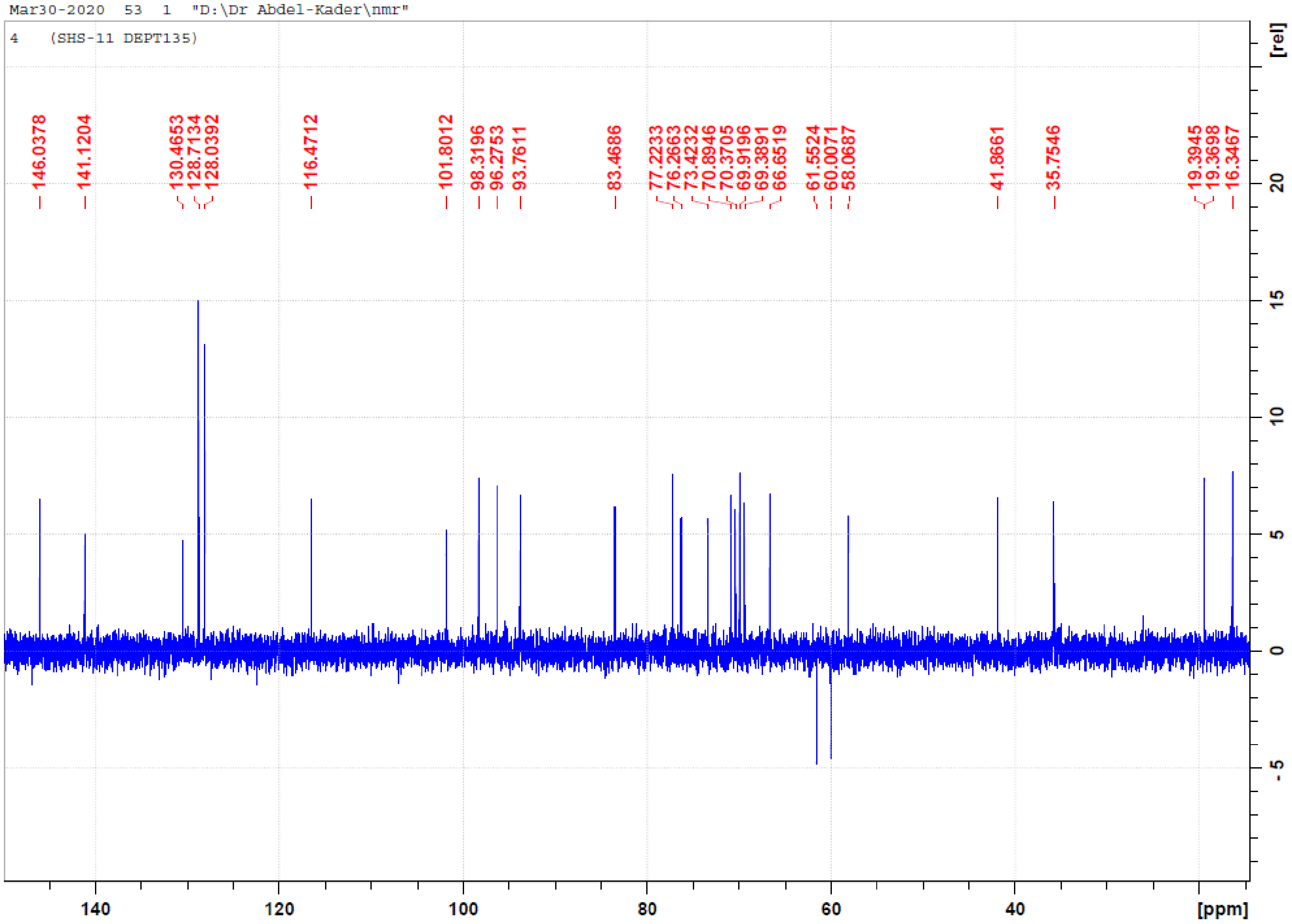


**Figure S89.** DEPT135 spectrum of compound 4.

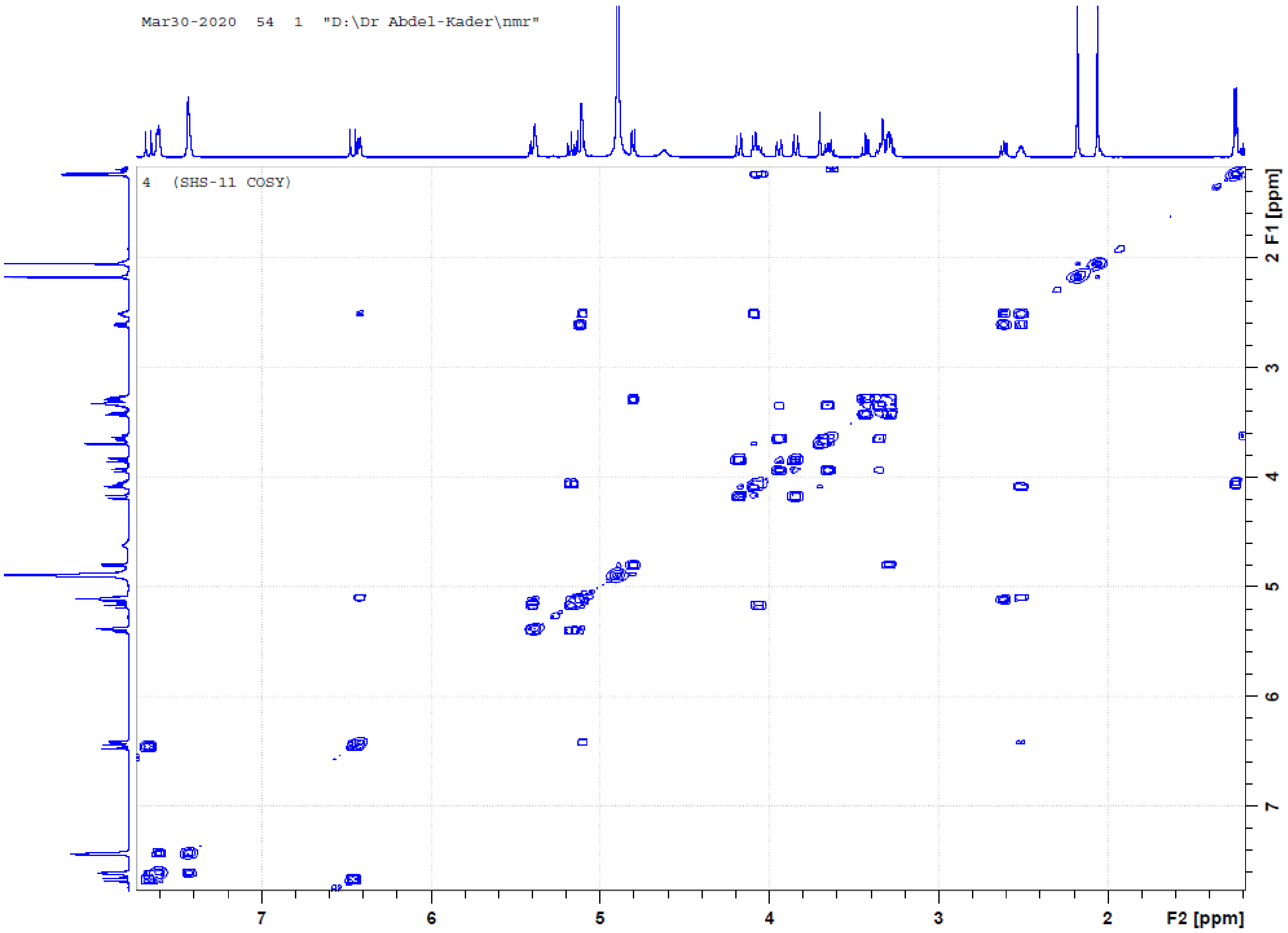


**Figure S90.** COSY spectrum of compound 4.

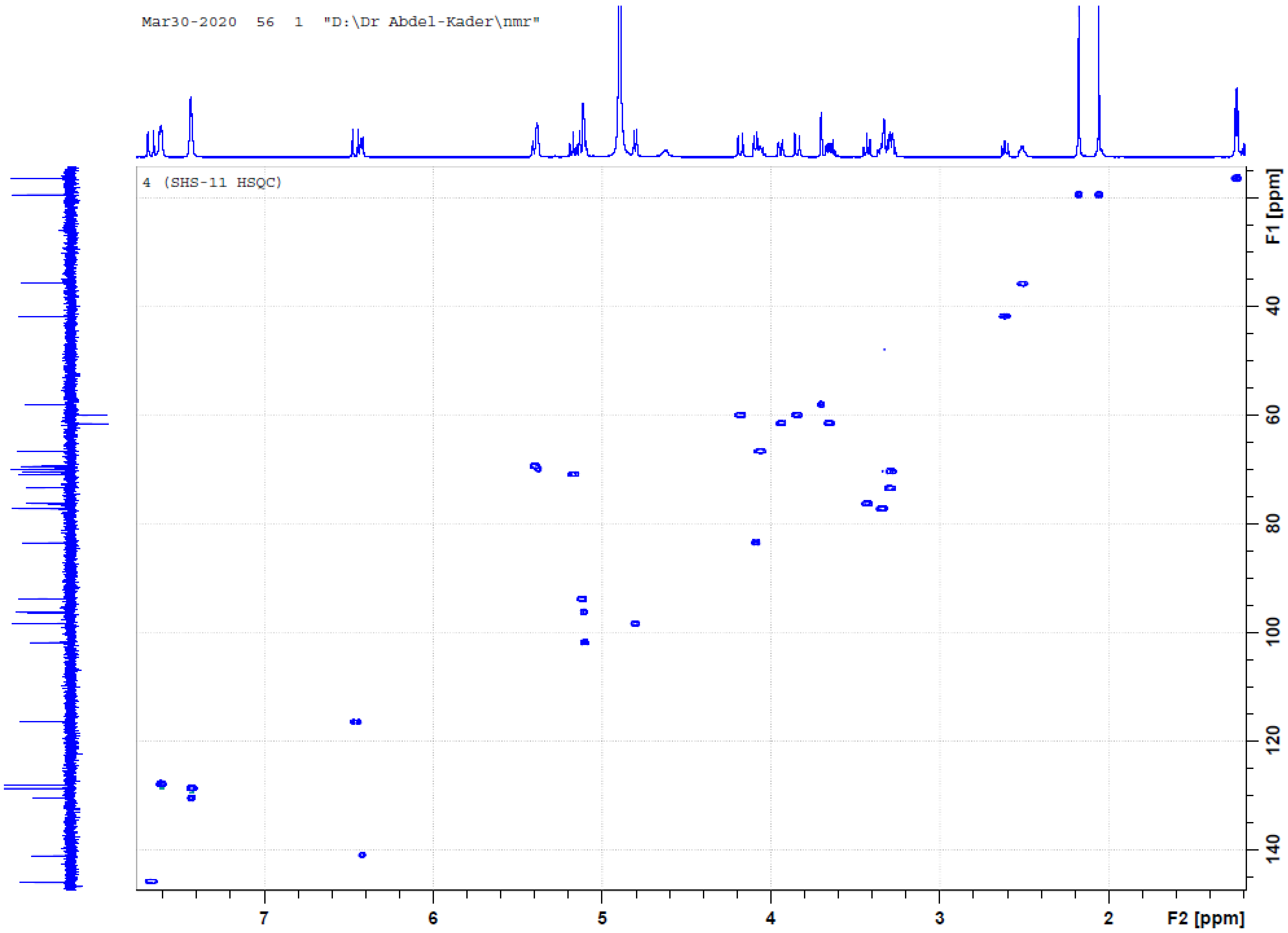


**Figure S91.** HSQC spectrum of compound 4.

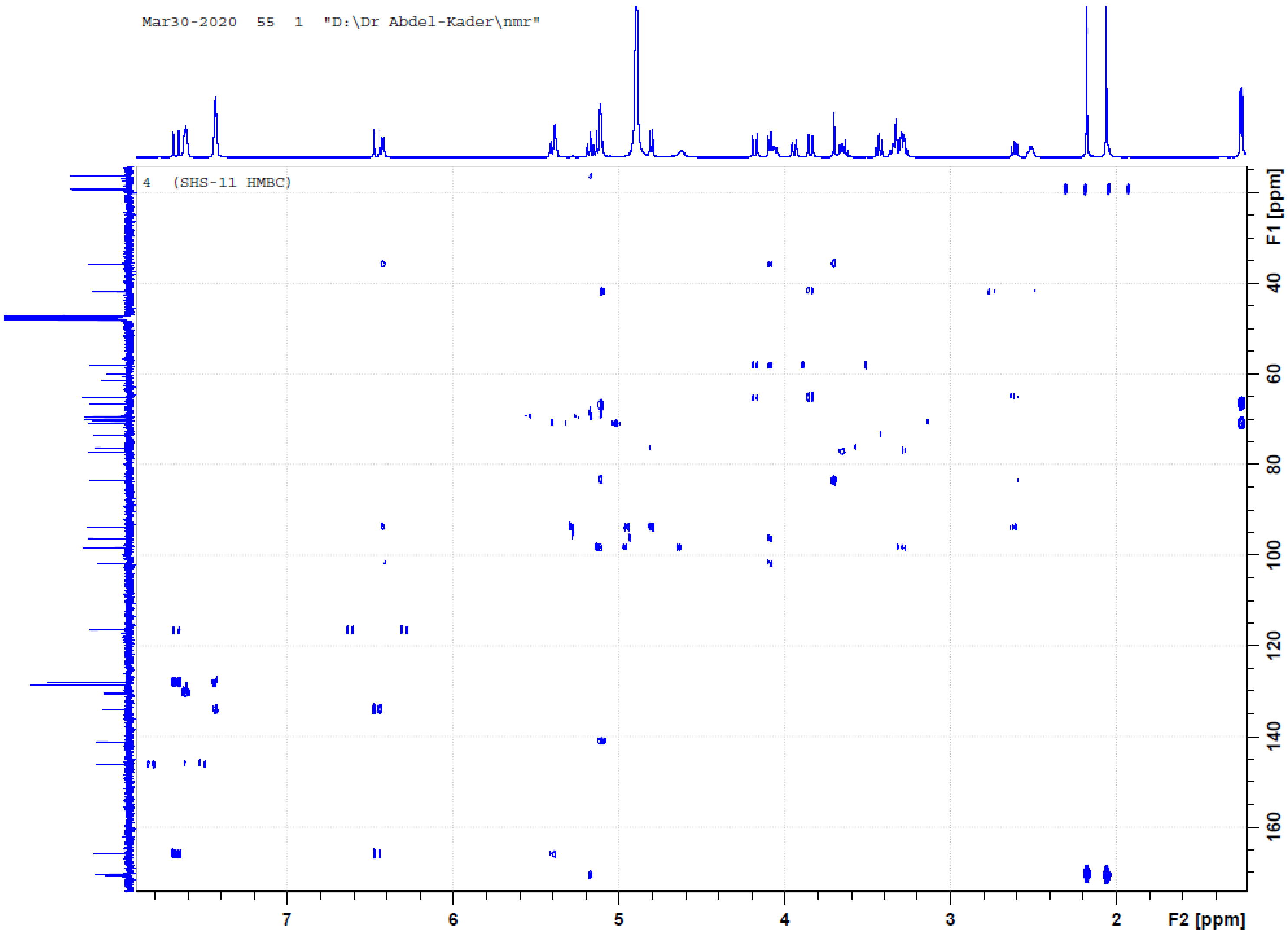


**Figure S92.** HMBC spectrum of compound 4.

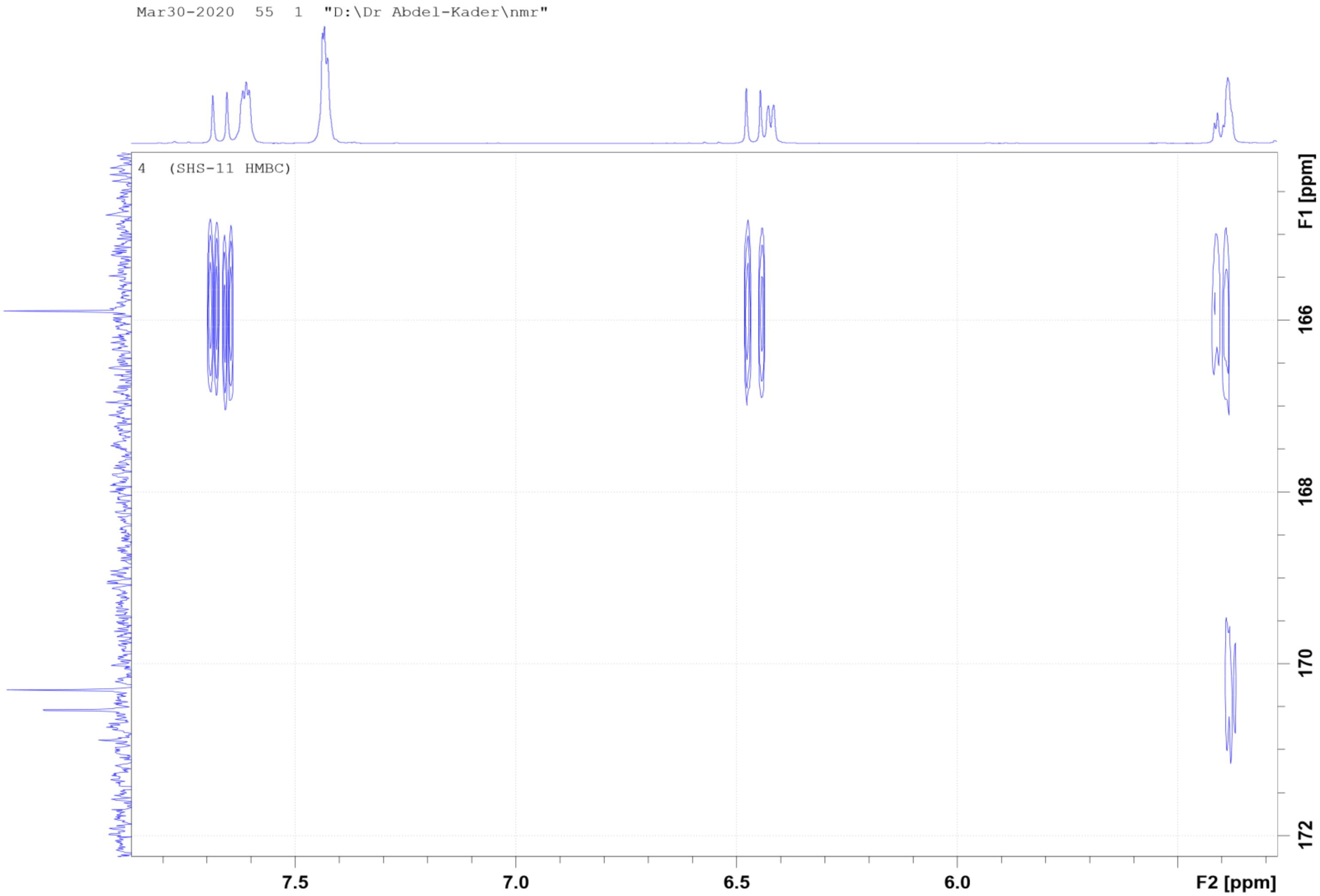


**Figure S93.** HMBC spectrum of compound 4 (expansion).

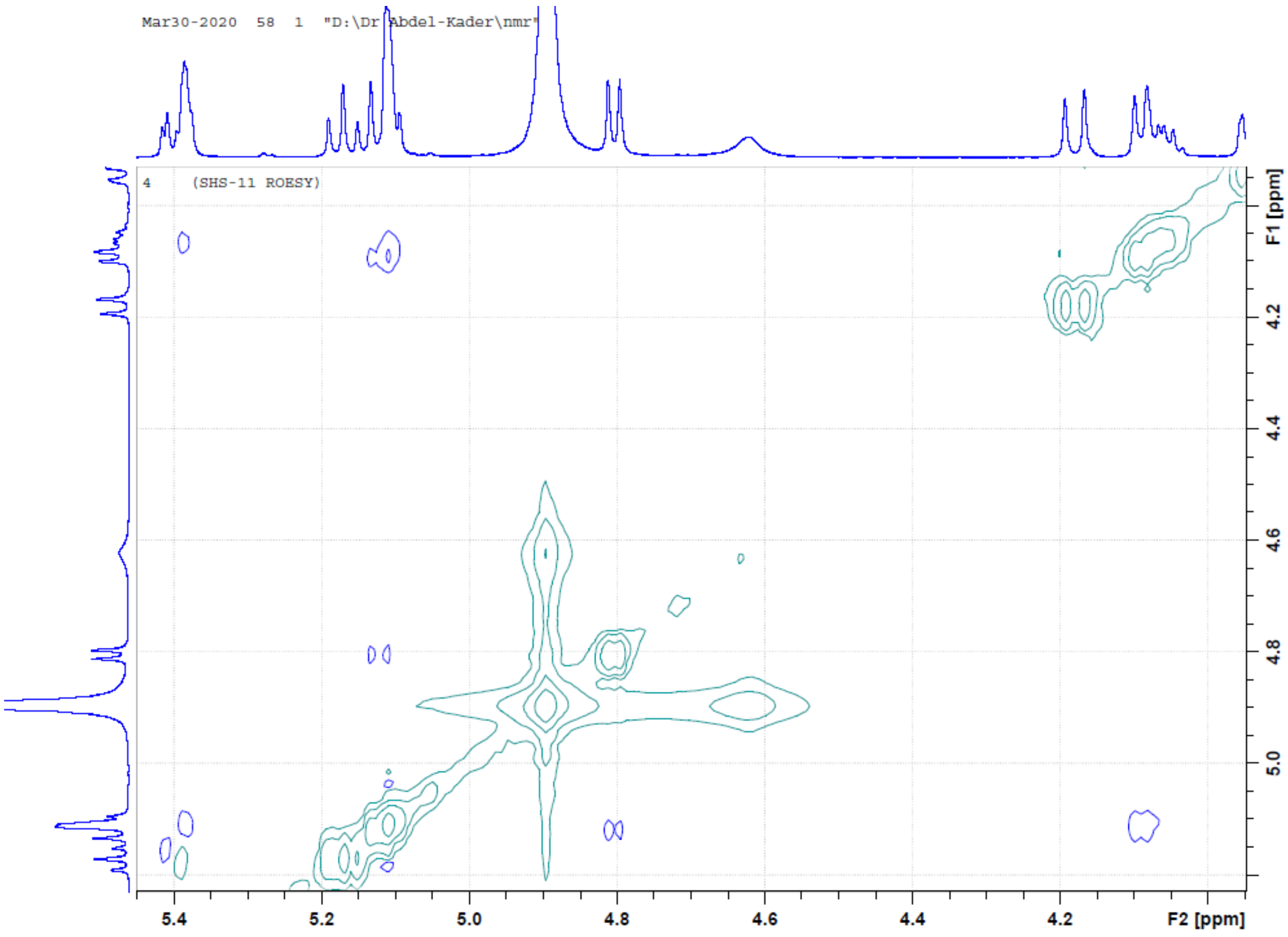


**Figure S94.** ROESY spectrum of compound 4 (expansion).

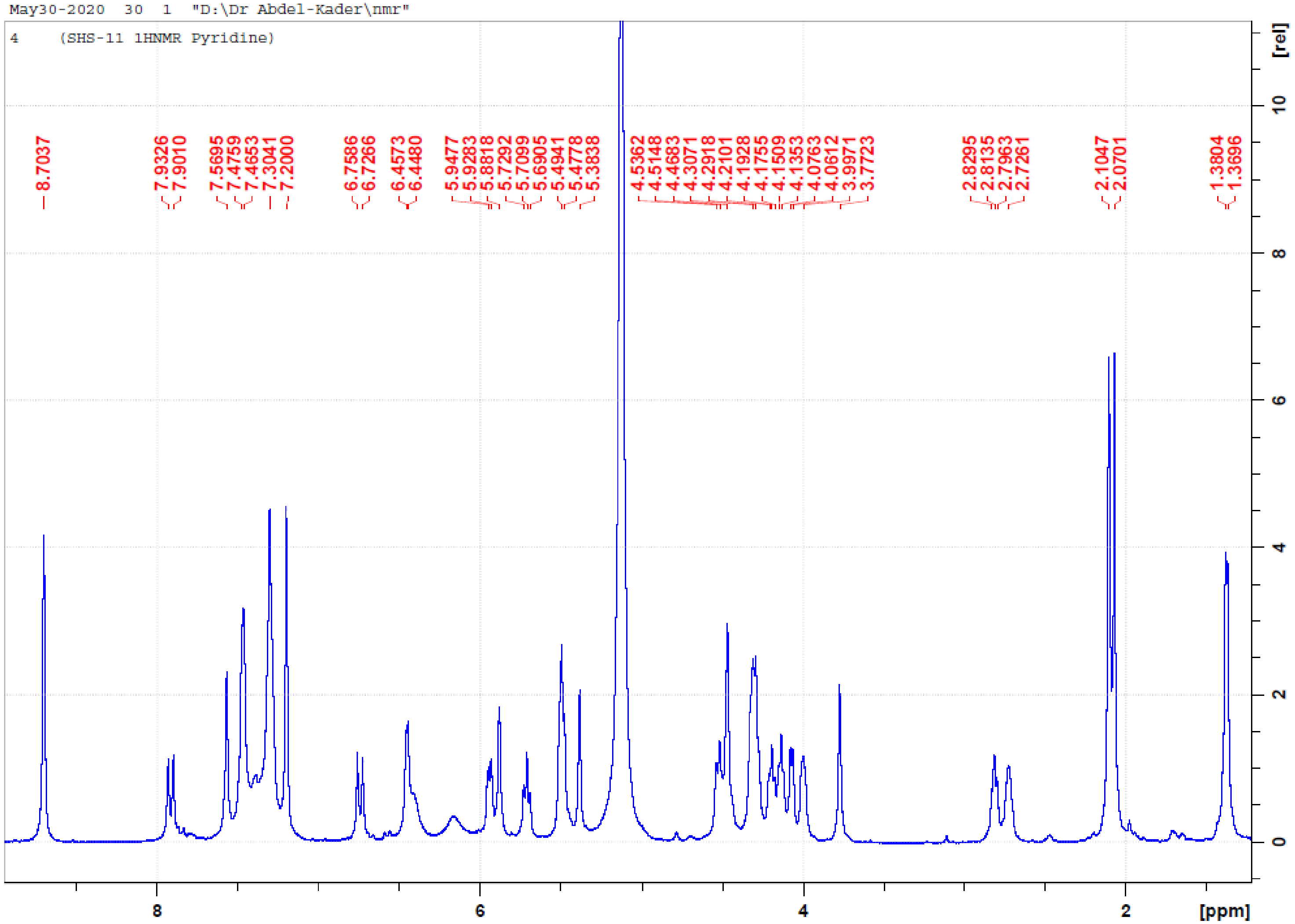


**Figure S95.** $^{1}$HNMR spectrum of compound 4 in Pyridine.

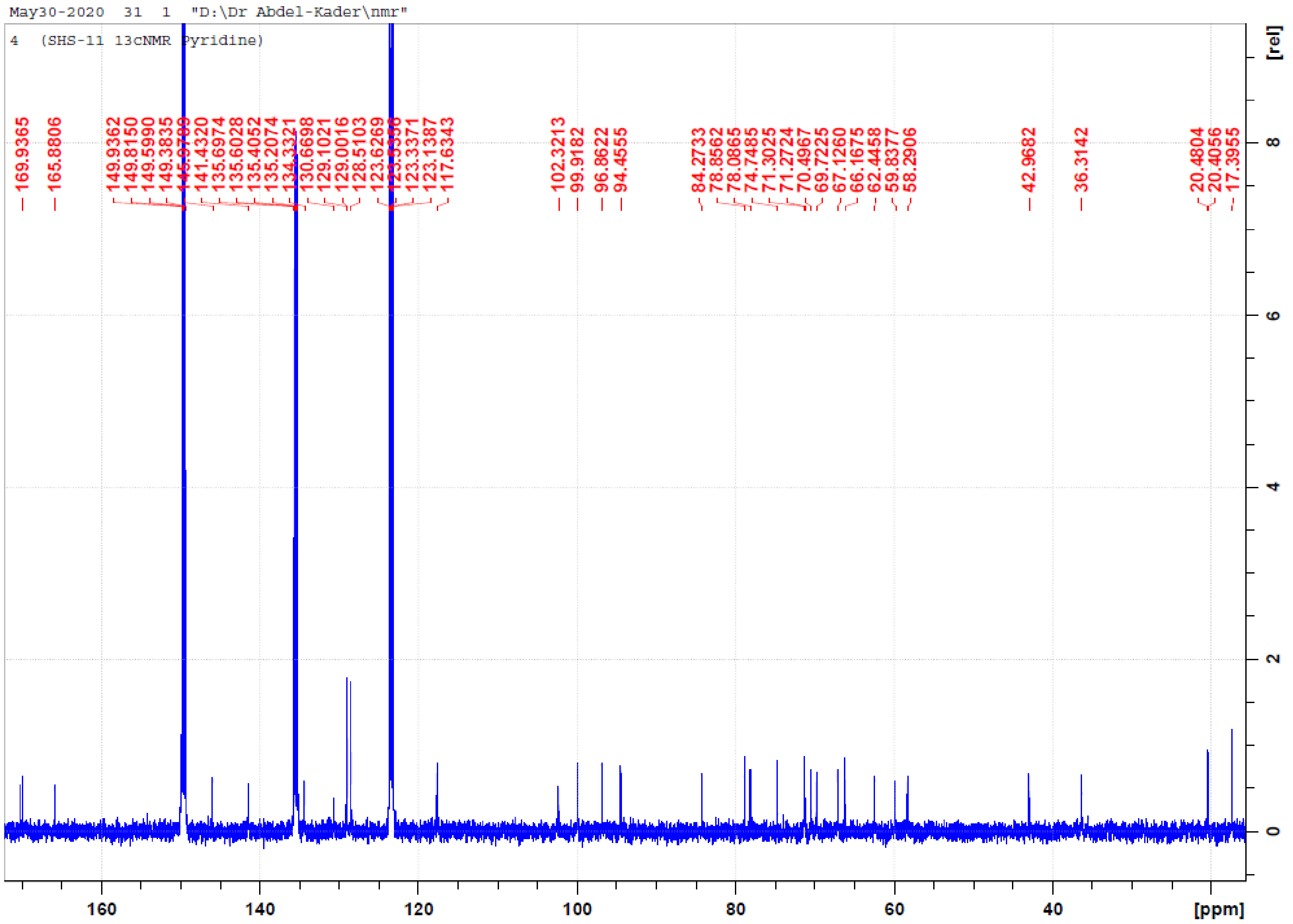


**Figure S96.** $^{13}$CNMR spectrum of compound 4 in Pyridine.

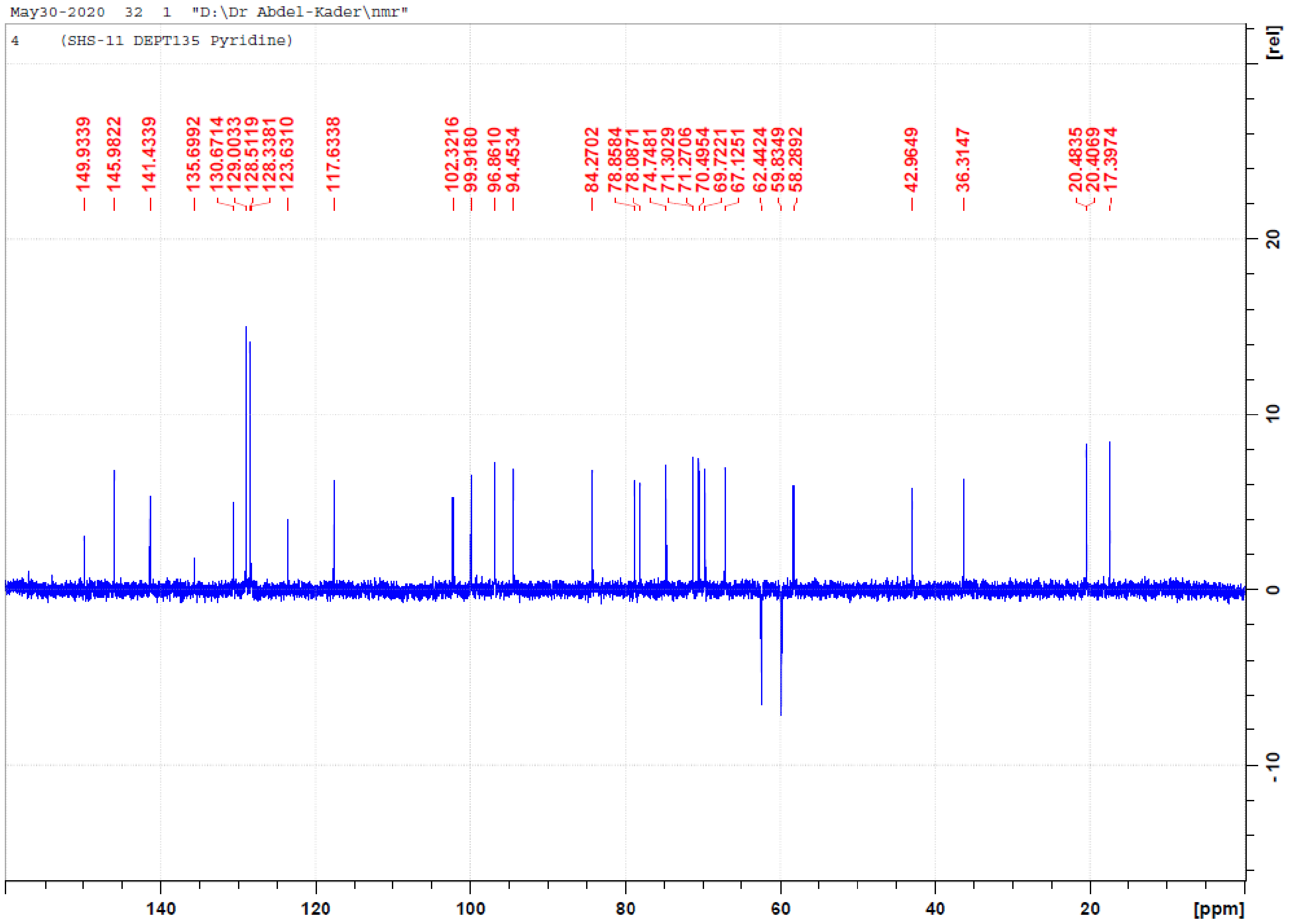


**Figure S97.** DEPT135 spectrum of compound 4 in Pyridine.

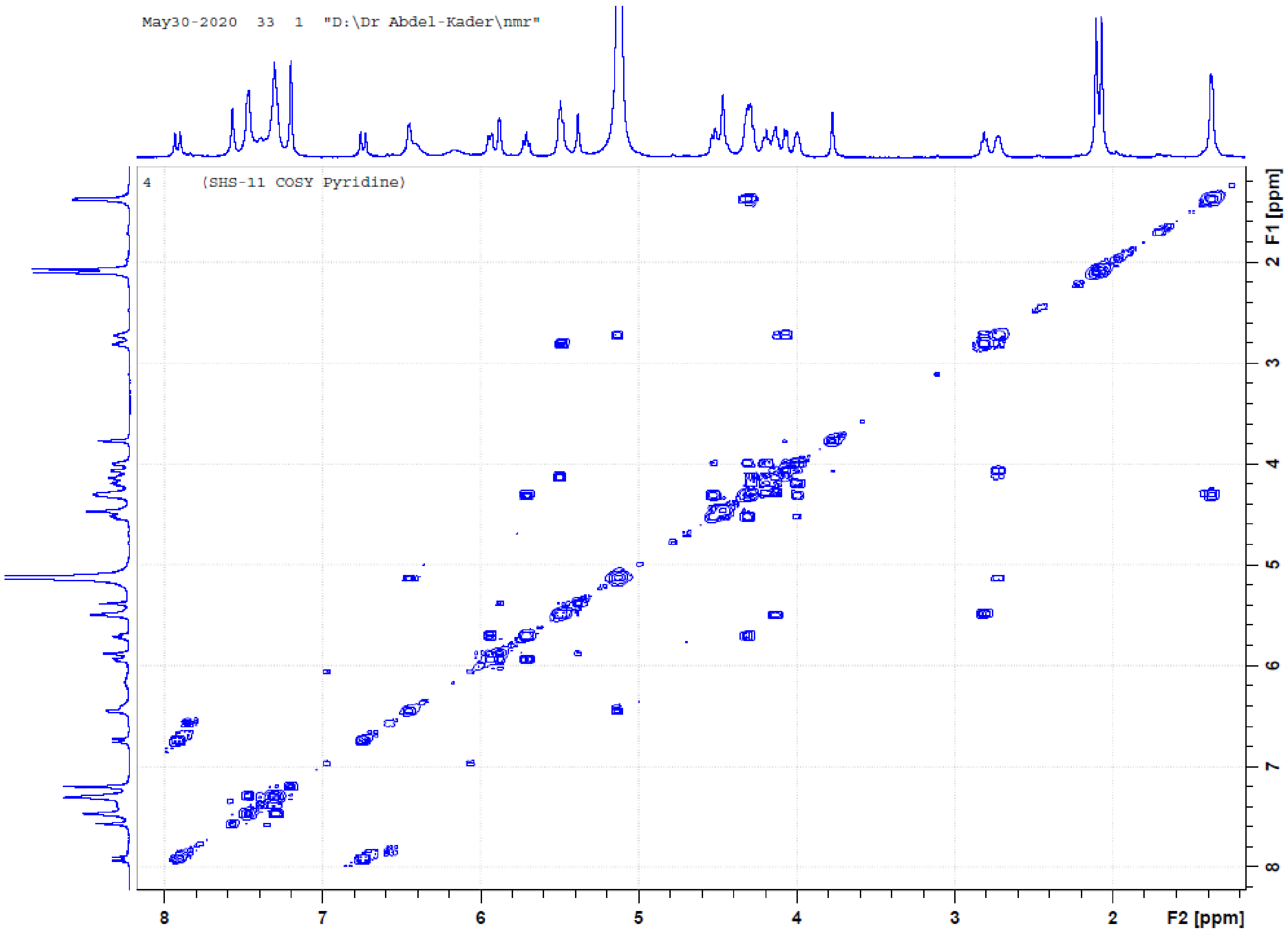


**Figure S98.** COSY spectrum of compound 4 in Pyridine.

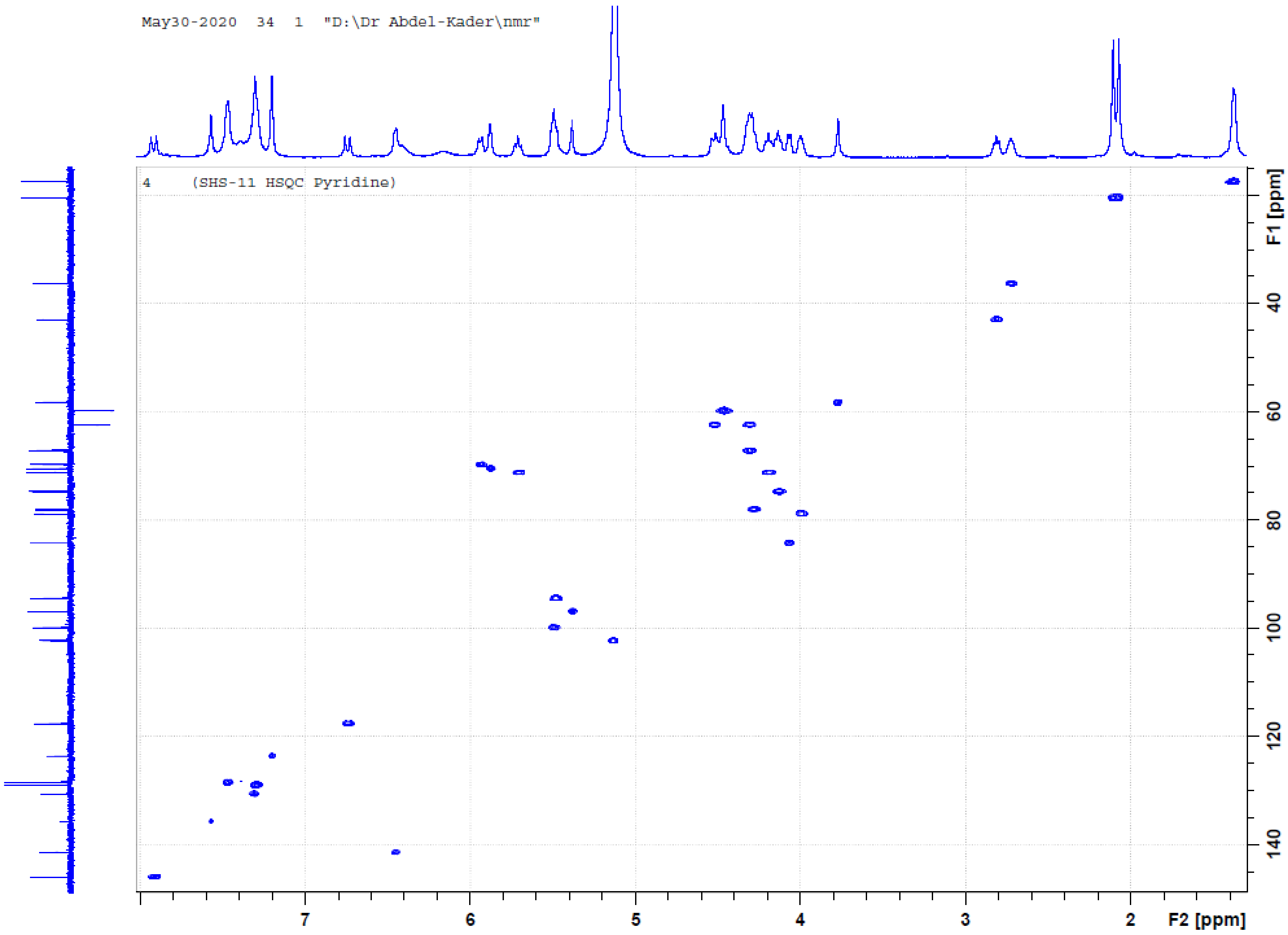


**Figure S99.** HSQC spectrum of compound 4 in Pyridine.

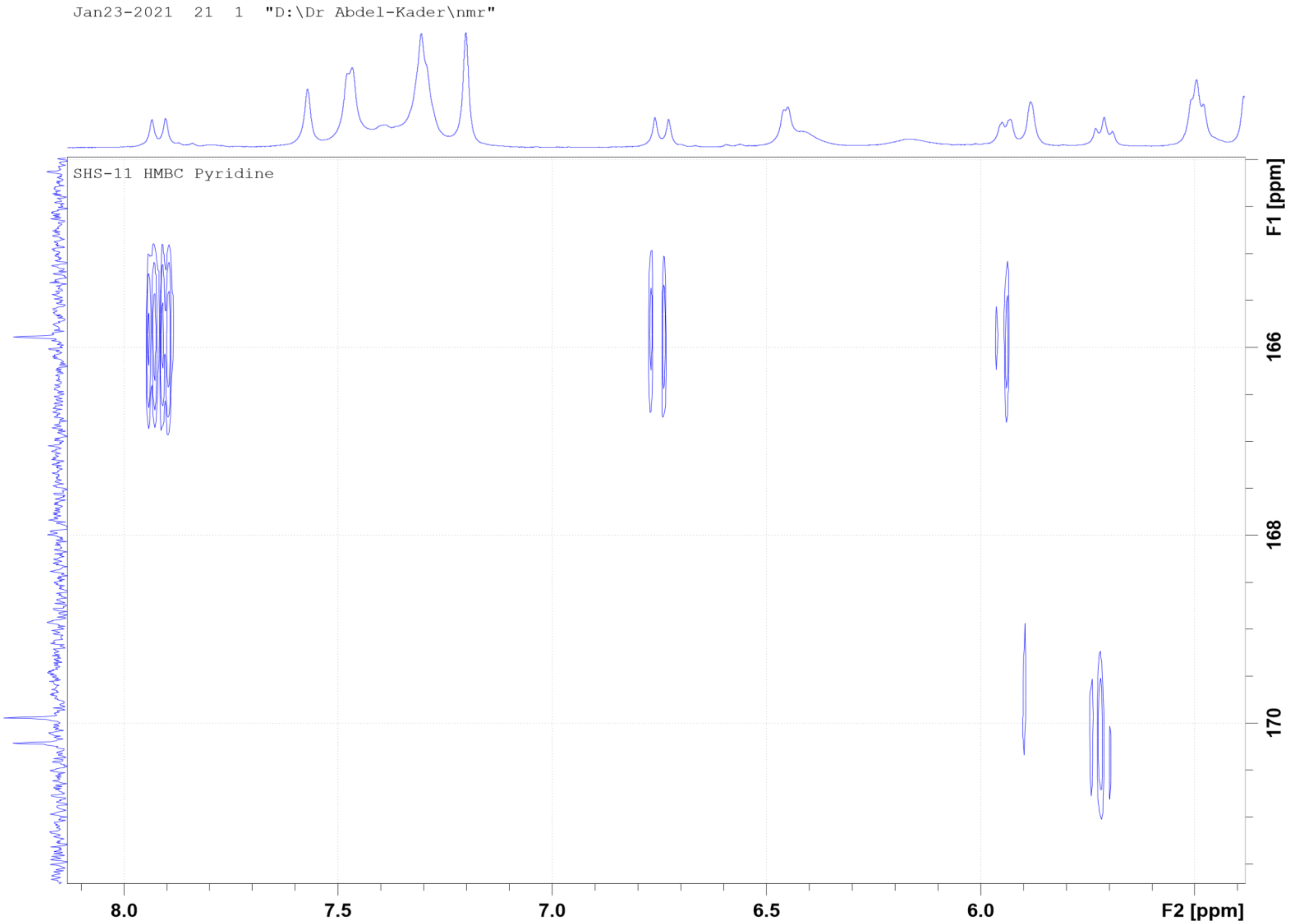


**Figure S100.** HMBC spectrum of compound 4 in Pyridine (expansion).

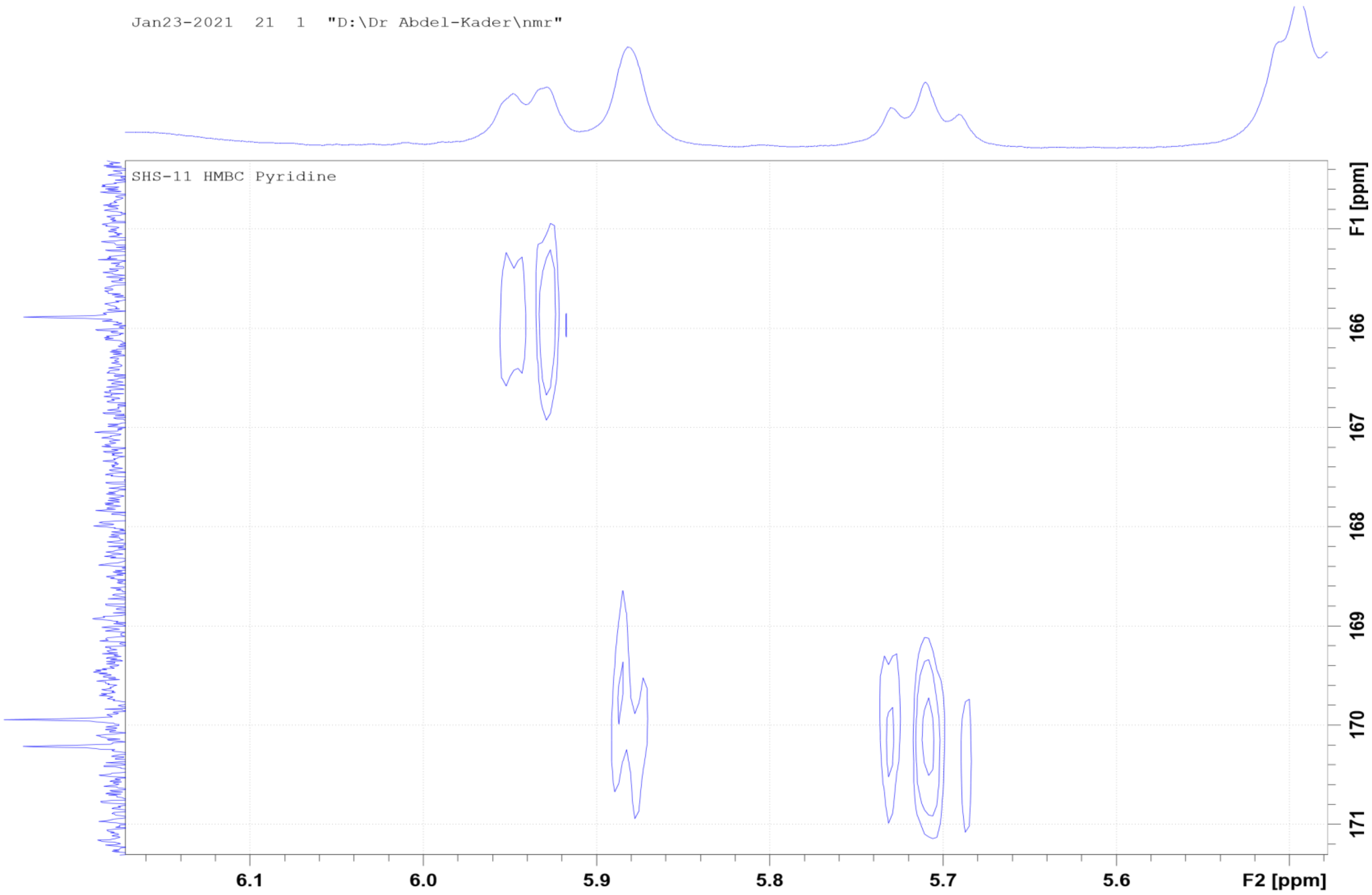


**Figure S101.** HMBC spectrum of compound 4 in Pyridine (expansion).

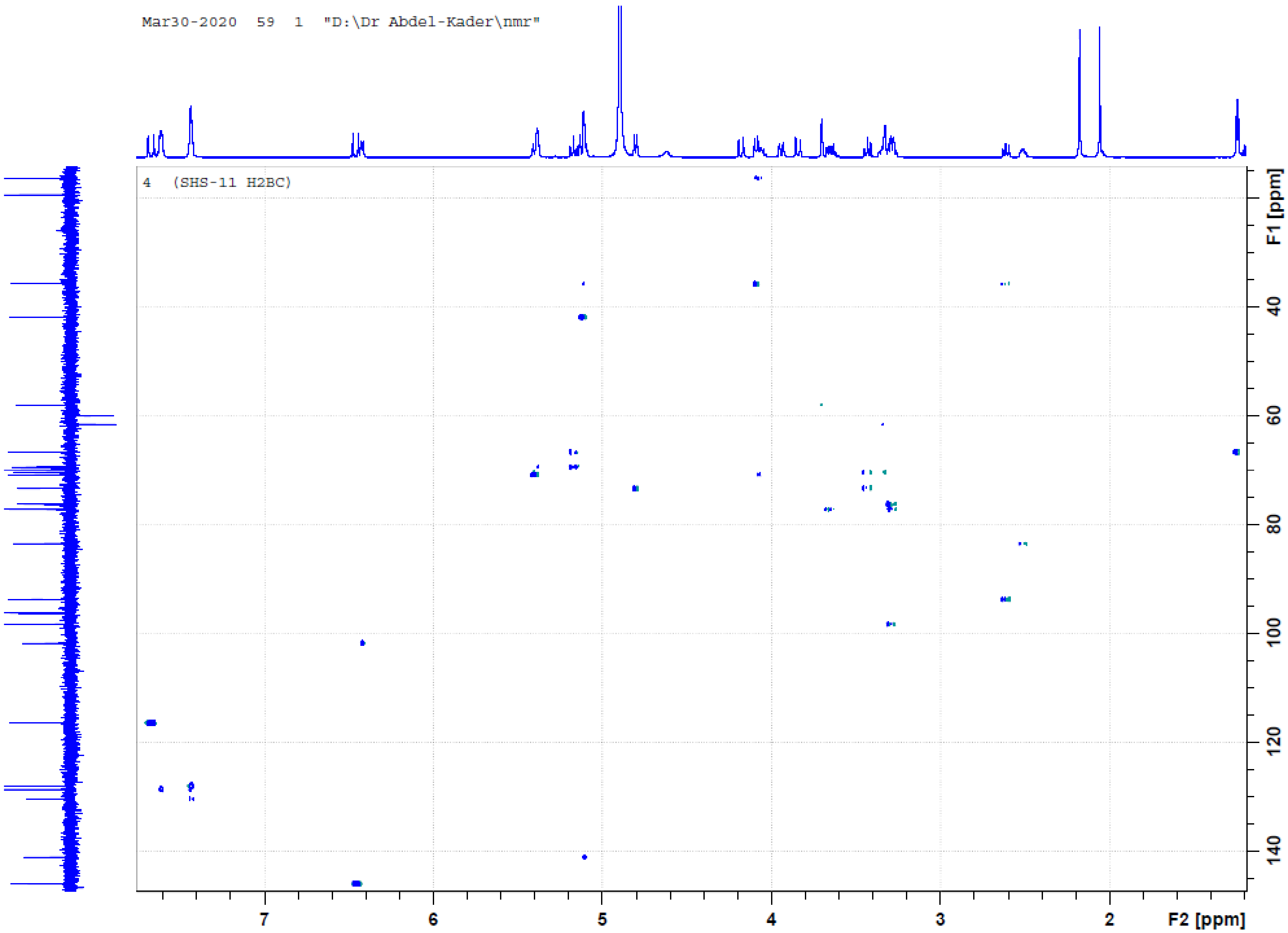


**Figure S102.** H2BC spectrum of compound 4 in Pyridine.

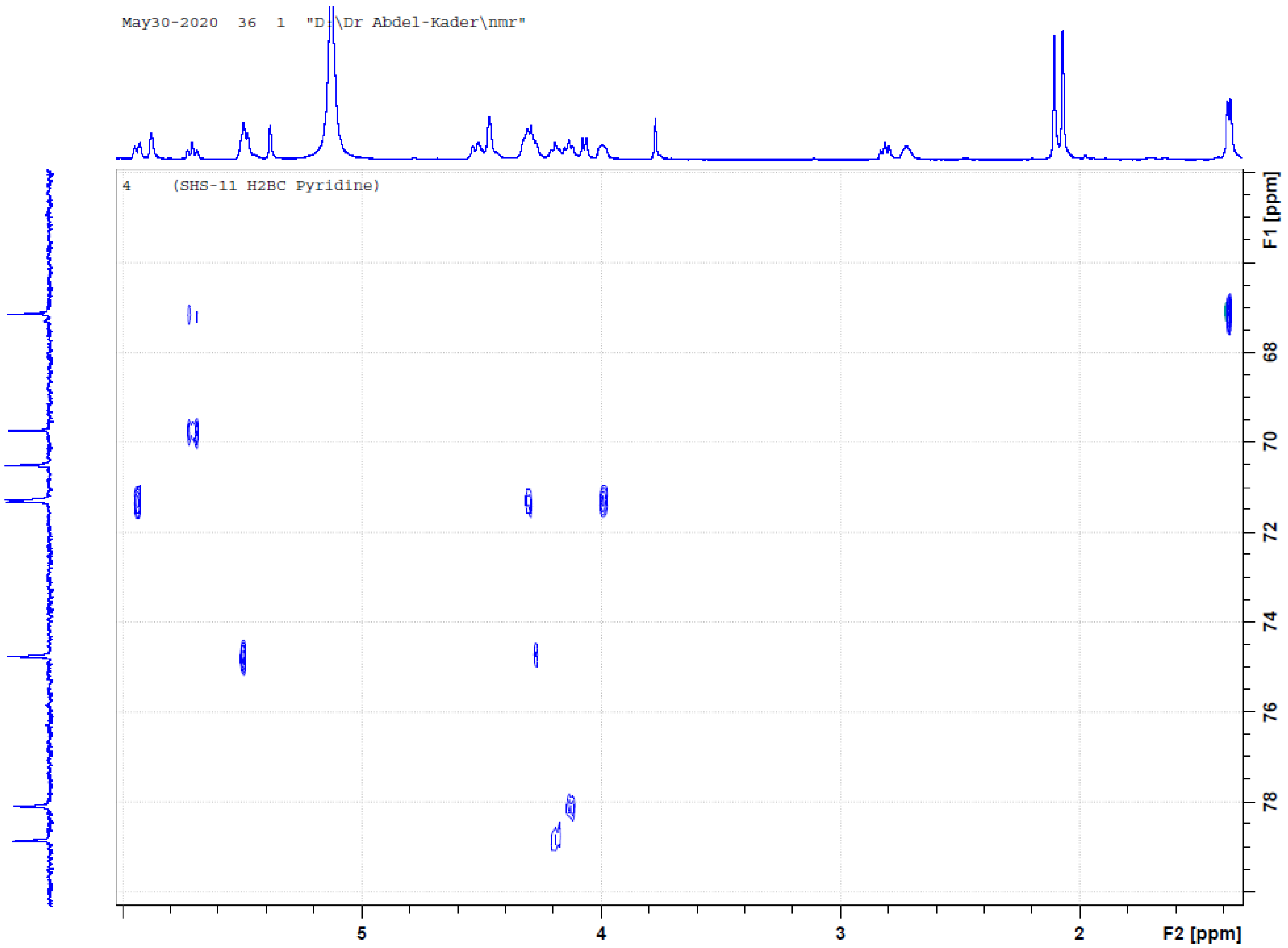


**Figure S103.** H2BC spectrum of compound 4 in Pyridine (expansion).

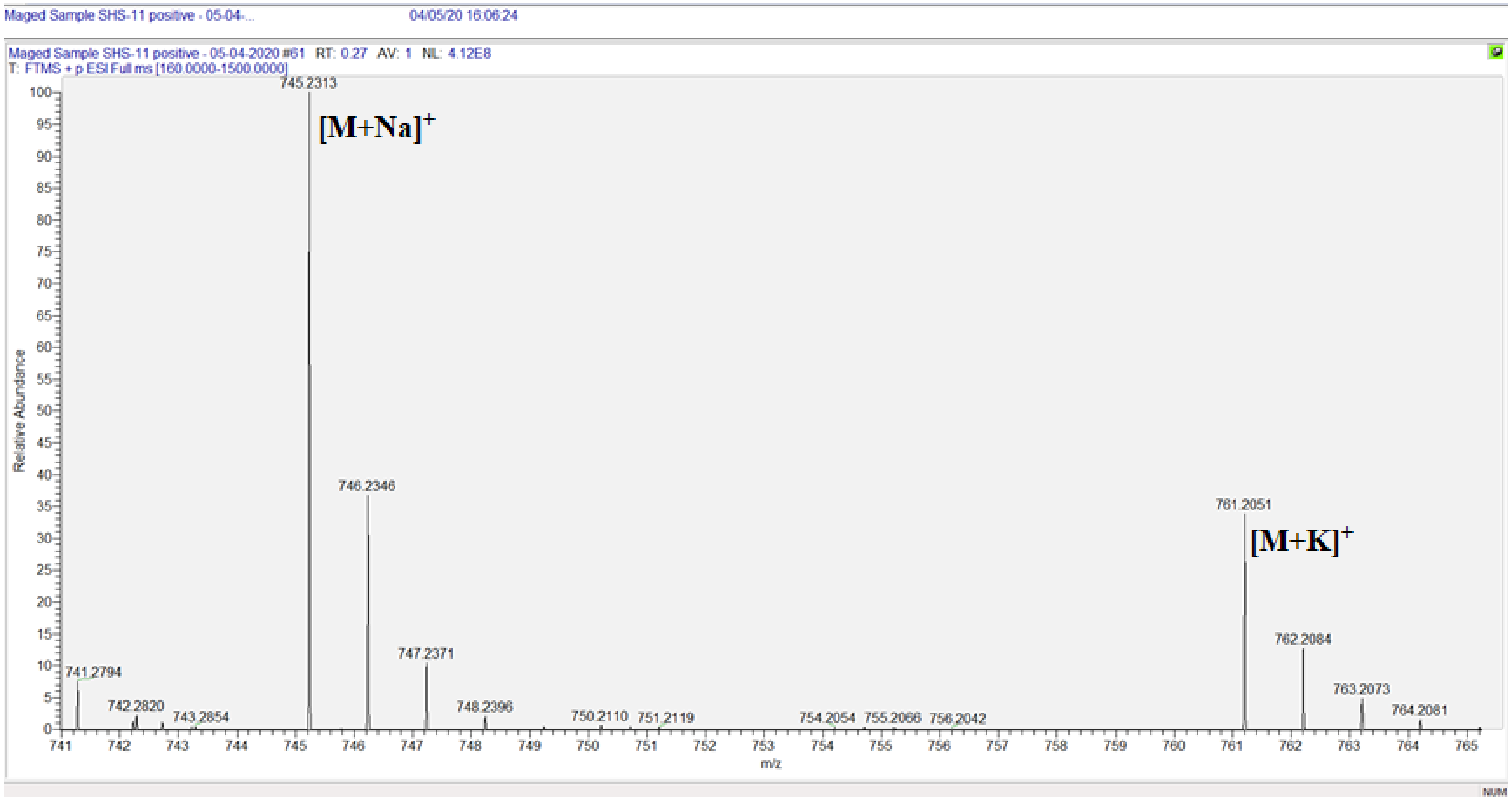


**Figure S104.** HRESIMS of compound 4 (positive mode).

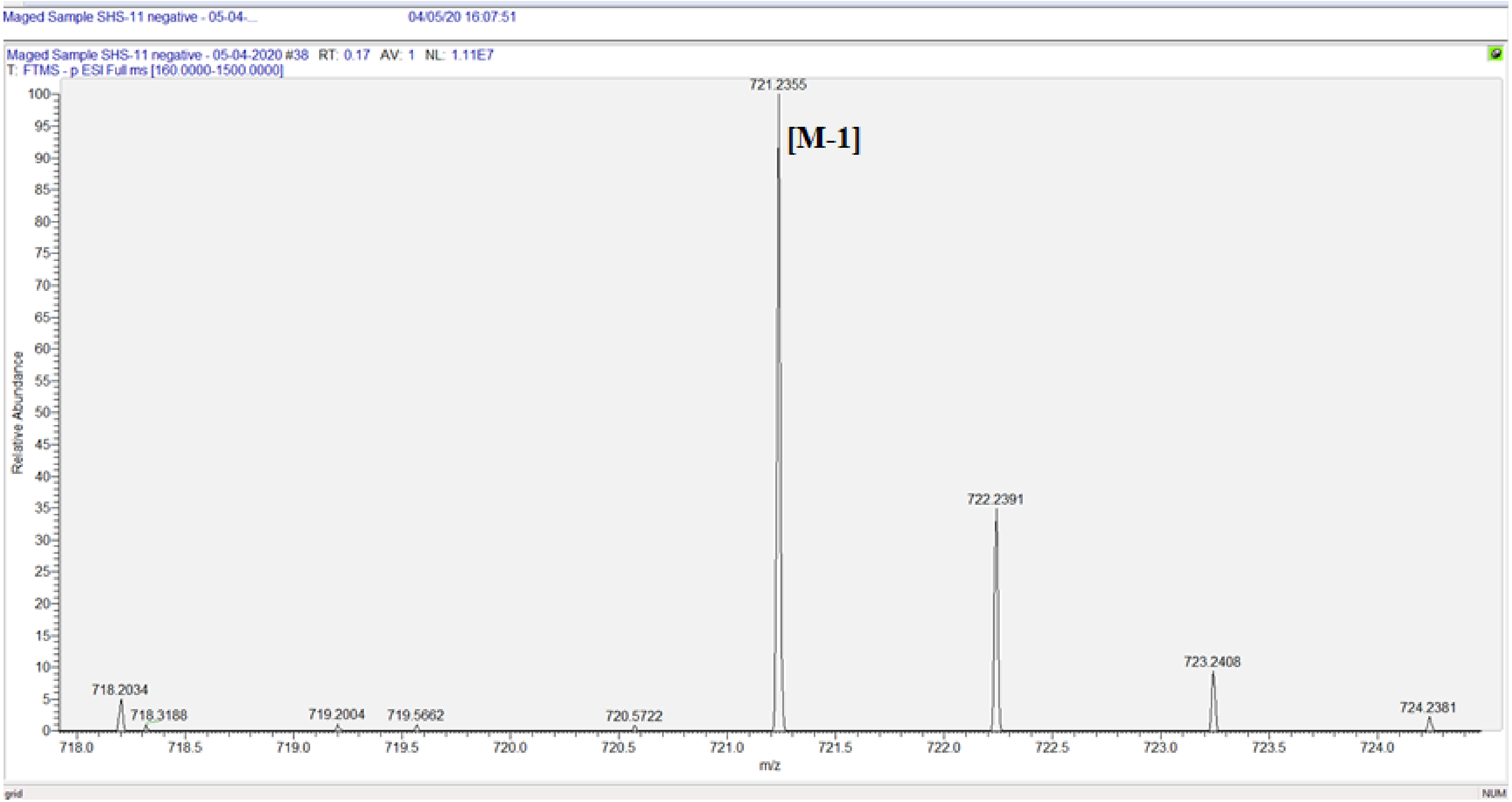


**Figure S105.** HRESIMS of compound 4 (negative mode).

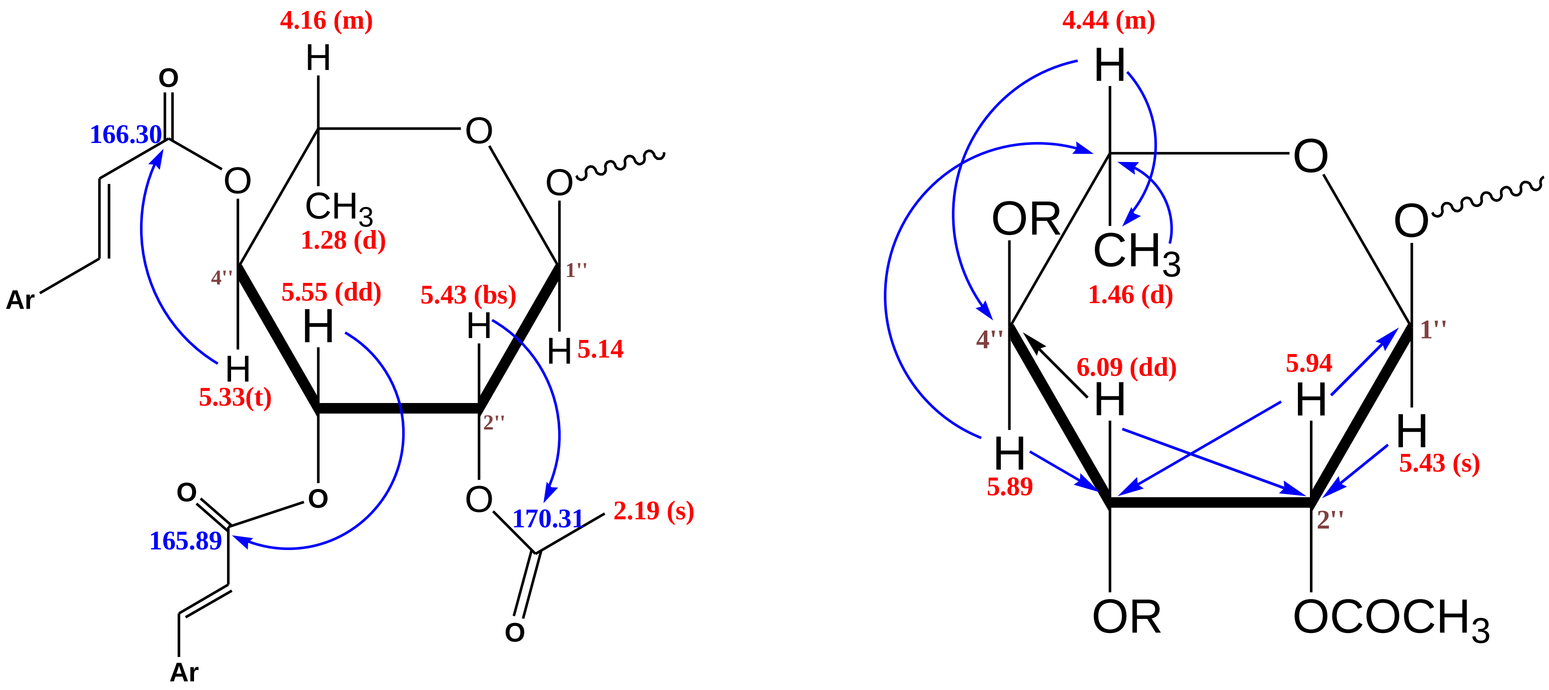


**Copyright:** © 2021 by the authors. Licensee MDPI, Basel, Switzerland. This article is an open access article distributed under the terms and conditions of the Creative Commons Attribution (CC BY) license (http://creativecommons.org/licenses/by/4.0/).

**(A)** **(B)**

**Figure S106.** HMBC in $CD_3OD$ (A) and H2BC in pyridine $d_6$ (B) correlations of the rhamnosyl moiety of **3**.

**Table S1.** Effect of compounds **2-4, 6** on liver Serum Biochemical Parameters.

| Treatment ($n$ = 5) | AST(u/l) | | ALT(u/l) | | GGT(u/l) | | ALP(u/l) | | Bilirubin(mg/dl) | |
|---|---|---|---|---|---|---|---|---|---|---|
| | (Mean ±S.E.) | % change | (Mean ± S.E.)) | % change | (Mean ± S.E.) | % change | (Mean ± S.E.) | % change | (Mean ± S.E.) | % change |
| **Normal** | 84.66±2.76 | | 23.31±1.84 | | 3.75±0.31 | | 326.00±16.23 | | 0.5±0.03 | |
| **Pa**[§] | 242.83±12.56***[a] | | 205.66±15.09*** [a] | | 12.03±0.89*** [a] | | 566.83±11.13*** [a] | | 2.40±0.13*** [a] | |
| **Sil**[§] | 151.16±13.54***[b] | 37.74 | 116.35±8.40*** [b] | 43.29 | 7.51±0.44*** [b] | 37.53 | 412.00±14.01*** [b] | 27.31 | 1.10±0.12*** [b] | 54.15 |
| **2 + Pa**[§] | 212.66±7.27 [b] | 12.42 | 192.33±10.58 [b] | 6.25 | 10.75±0.59 [b] | 10.66 | 526.33±14.75* [b] | 7.14 | 2.31±0.06** [b] | 3.87 |
| **3 + Pa**[§] | 186.33±7.24** [b] | 23.26 | 136.00±7.11** [b] | 33.71 | 7.46±0.30*** [b] | 37.95 | 472.33±11.87*** [b] | 16.67 | 1.74±0.09** [b] | 27.70 |
| **4 + Pa**[§] | 209.16±5.68* [b] | 13.86 | 150.33±6.75** [b] | 26.72 | 7.68±0.19*** [b] | 36.14 | 487.50±9.68*** [b] | 13.99 | 1.80±0.06** [b] | 25.00 |
| **6 + Pa**[§] | 208.33±7.05* [b] | 14.20 | 188.33±11.74 [b] | 7.96 | 9.36±0.27* [b] | 22.16 | 518.33±13.04* [b] | 8.55 | 2.19±0.13 [b] | 8.79 |

* $p < 0.05$, ** $p < 0.01$, *** $p < 0.00$ [a] as compared to control group [b] As compared to Pa grou**p** [§] Doses: Pa (500 mg/kg), Sil, **2-4**, **6** (20.7 µmole/kg).

**Table S2.** Effect of compounds **2-4, 6** on liver and kidney TP and NP-SH.

| Treatment | Liver | | Kidney | |
|---|---|---|---|---|
| ($n$ = 5) | TP (g/l) | NP-SH(nmol/g) | TP (g/l) | NP-SH(nmol/g) |
| **Normal** | 77.93±2.55 | 3.91±0.09 | 91.87±7.95 | 0.81±0.02 |
| **Pa**[§] | 24.31±1.96***[a] | 1.45±0.14***[a] | 26.55±3.40***[a] | 0.26±0.02***[a] |
| **Sil**[§] | 73.04±6.63***[b] | 3.37±0.28***[b] | 60.54±5.17***[b] | 0.74±0.03***[b] |
| **2 + Pa**[§] | 26.15±3.14 [b] | 1.44±0.19 [b] | 30.55±2.30 [b] | 0.36±0.06 [b] |
| **3 + Pa**[§] | 37.36±3.72* [b] | 3.43±0.30*** [b] | 46.25±2.73*** [b] | 0.74±0.06*** [b] |
| **4 + Pa**[§] | 36.82±3.72* [b] | 2.72±0.20**** [b] | 43.04±2.44** [b] | 0.52±0.05* [b] |
| **6 + Pa**[§] | 36.08±2.55** [b] | 2.54±0.28** [b] | 39.80±4.04* [b] | 0.48±0.05 [b] |

* $p < 0.05$, ** $p < 0.01$, *** $p < 0.00$ [a] as compared to control group [b] As compared to Pa group [§] Doses: Pa (500 mg/kg), Sil, **2-4**, **6** (20.7 µmole/kg).

**Table S3.** Effect of compounds **2-4, 6** on kidney Serum Biochemical Parameters.

| **Treatment (*n* = 5)** | **Urea(mg/dl)** | | **Creatinine(mg/dl)** | | **Sodium(mmol/l)** | | **Potassium(mmol/l)** | |
|---|---|---|---|---|---|---|---|---|
| | **(Mean ±)** | **% change** | **(Mean ±)** | **% change** | **(Mean ±)** | **% change** | **(Mean ±)** | **% change** |
| **Normal** | 52.05±3.35 | | 2.48±0.21 | | 63.91±7.51 | | 6.10±0.36 | |
| **Pa**§ | 142.00±5.92*** a | | 13.93±1.00*** a | | 191.83±9.41*** a | | 14.51±0.82*** a | |
| **Sil**§ | 85.38±5.10*** b | 39.87 | 6.98±0.26*** b | 49.88 | 81.60±9.52*** b | 57.46 | 7.83±0.36*** b | 46.03 |
| **2 + Pa**§ | 132.83±5.17 b | 6.45 | 11.31±0.27* b | 18.77 | 185.50±14.02 b | 3.30 | 12.66±0.62 b | 12.47 |
| **3 + Pa**§ | 107.05±5.86** b | 24.61 | 8.91±0.19*** b | 36.00 | 126.83±5.27*** b | 33.88 | 9.36±0.44*** b | 35.47 |
| **4 + Pa**§ | 116.66±5.54* b | 17.84 | 9.31±0.30** b | 33.13 | 152.16±7.24* b | 20.67 | 9.93±0.30*** b | 31.57 |
| **6 + Pa**§ | 121.66±4.12* b | 14.31 | 10.93±0.39* b | 21.53 | 181.16±6.93 b | 5.56 | 10.06±0.46** b | 30.65 |

* $p < 0.05$, ** $p < 0.01$, *** $p < 0.00$ [a] as compared to control group [b] As compared to Pa group § Doses: Pa (500 mg/kg), Sil, **2-4**, **6** (20.7 µmole/kg).
